# Robust single-cell DNA methylome profiling with snmC-seq2

**DOI:** 10.1038/s41467-018-06355-2

**Published:** 2018-09-20

**Authors:** Chongyuan Luo, Angeline Rivkin, Jingtian Zhou, Justin P. Sandoval, Laurie Kurihara, Jacinta Lucero, Rosa Castanon, Joseph R. Nery, António Pinto-Duarte, Brian Bui, Conor Fitzpatrick, Carolyn O’Connor, Seth Ruga, Marc E. Van Eden, David A. Davis, Deborah C. Mash, M. Margarita Behrens, Joseph R. Ecker

**Affiliations:** 1 Genomic Analysis Laboratory, The Salk Institute for Biological Studies, La Jolla, CA 92037 USA; 2 Howard Hughes Medical Institute, The Salk Institute for Biological Studies, La Jolla, CA 92037 USA; 3 Swift Biosciences Inc., 58 Parkland Plaza, Suite 100, Ann Arbor, MI 48103 USA; 4 Computational Neurobiology Laboratory, The Salk Institute for Biological Studies, La Jolla, CA 92037 USA; 5 Flow Cytometry Core Facility, The Salk Institute for Biological Studies, La Jolla, CA 92037 USA; 6 Zymo Research Corporation, Irvine, CA 92614 USA; 7 Department of Neurology, Miller School of Medicine, University of Miami, Miami, FL 33136 USA

## Abstract

Single-cell DNA methylome profiling has enabled the study of epigenomic heterogeneity in complex tissues and during cellular reprogramming. However, broader applications of the method have been impeded by the modest quality of sequencing libraries. Here we report snmC-seq2, which provides improved read mapping, reduced artifactual reads, enhanced throughput, as well as increased library complexity and coverage uniformity compared to snmC-seq. snmC-seq2 is an efficient strategy suited for large-scale single-cell epigenomic studies.

## Abstract (web summary)

Single-cell DNA methylome profiling allows the study of epigenomic heterogeneity in tissues but has been impeded by library quality. Here the authors demonstrate snmC-seq2 which improves mapping, throughput and library complexity.

## Introduction

Several library preparation strategies for single-cell DNA methylome profiling have been developed based upon the post-bisulfite adapter tagging (PBAT) approach and applied to study epigenomic heterogeneity in embryonic stem cells, mouse and human brains, and preimplantation embryos^1–4^. Most library preparation methods for single-cell methylomes use random-primed DNA synthesis to incorporate the upstream sequencing adapter but vary in the strategies that incorporate the downstream adapter sequence. A second round of random-primed synthesis is used by scBS-seq, whereas sc-WGBS and snmC-seq use proprietary 3′-adaptor tagging methods^2,3,5^. A combinatorial indexing strategy has recently been applied to generate single-cell methylome libraries without physically compartmentalizing individual cells^6^. Sequencing libraries generated by existing single-cell methylome methods suffer from one or more of the following problems: high levels of artifactual sequences such as adapter dimers, low mapping rate, or small insert size. We previously developed snmC-seq, which provides improved read mapping^5^. However, compared to traditional MethylC-seq^7^, snmC-seq libraries still contain substantially higher levels of adapter dimer sequences and have lower read mapping rates. We systematically examined experimental factors that can be modified to improve the quality of snmC-seq libraries resulting in the development of snmC-seq2.

## Results

### Efficient read mapping in snmC-seq2

A detailed step-by-step bench protocol is provided in the Supplementary Methods.

**Fig. 1:**
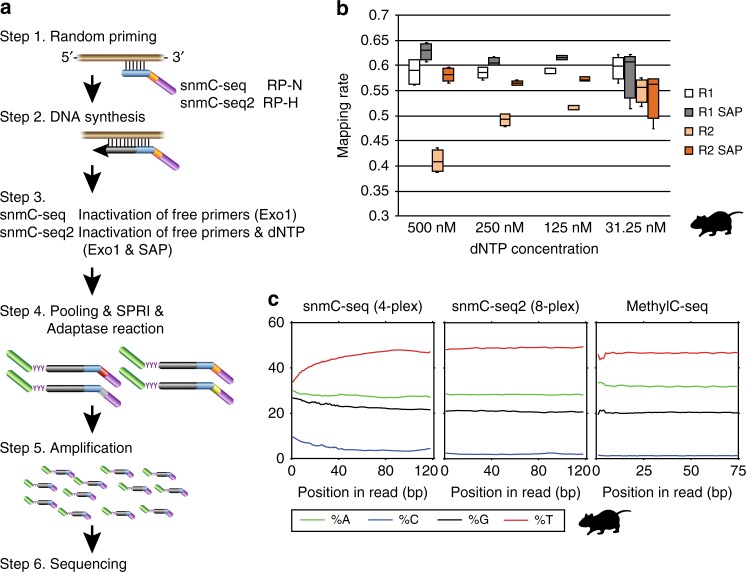
Improvement of single-cell methylome read mapping. **a** Schematics of snmC-seq workflow. Steps modified in snmC-seq2 are highlighted. RP-N and RP-H indicate random primers used in snmC-seq and snmC-seq2, respectively. Exo1 exonuclease 1, SAP shrimp alkaline phosphatase, SPRI solid-phase reversible immobilization. **b** Reverse read (R2) mapping is improved by reducing nucleotide concentration in random-primed DNA synthesis reaction or treatment with shrimp alkaline phosphatase, whereas forward read (R1) mapping is not affected. Each condition contained four biological replicates. **c** snmC-seq2 produces reverse reads with comparable base composition as MethylC-seq reads. The elements of all box-plots are defined as following—center line, median; box limits, first and third quartiles; whiskers, 1.5× interquartile range

We first set out to develop strategies to improve the mapping rate of snmC-seq reads by examining the snmC-seq dataset generated from mouse frontal cortical neurons^5^. We found that the mapping rate of reverse reads (R) generated by paired-end sequencing is significantly lower in multiplexed (4-plex) than non-multiplexed (1-plex) snmC-seq (Supplementary Fig. 1a, *p* = 1.6 × 10^–32^, *t*-test)^5^, whereas the mapping rate of forward reads (F) was comparable between multiplexed and non-multiplexed libraries. We identified an aberrant base composition in the reverse reads of multiplexed snmC-seq data (Supplementary Fig. 1b). After the conversion of >99% unmethylated cytosines (C) to uracils (U) with bisulfite treatment, only 1.2% nucleotides of mouse frontal cortical neuron genomes are expected to be read as C, whereas 48% of nucleotides are expected to be read as T during sequencing^8^. However, multiplexed snmC-seq data showed an elevated frequency of C at the beginning of reverse reads and reached a maximum of 9.6% and a decreased frequency of T with a minimal of 33.7% (Supplementary Fig. 1b). We hypothesized that the aberrant elevation of C and decrease of T frequency was caused by carryover nucleotides triphosphate (dNTP) that were incompletely removed by DNA purification using solid-phase reversible immobilization (SPRI) beads after the random-primed DNA synthesis (Fig. 1a). The change of nucleotide composition is consistent with a transition from the characteristic methylome reads (e.g. 1.2% C and 48% T) to synthetic sequence generated by a tailing reaction containing all four nucleotides. Multiplexed snmC-seq is associated more severe contamination since carryover dNTPs from four random-primed DNA synthesis reactions were combined into one Adaptase reaction. The subsequent 3′-tagging by Adaptase involves simultaneous tailing and adaptor ligation (step 4 in Fig. 1a). While random-primed DNA synthesis requires all four nucleotides (A, T, C, and G), the tailing reaction mediated by Adaptase uses only two nucleotides and generates a synthetic low-complexity, short tail sequence with an average length of 8 bp that is present at the beginning of reverse reads. Contamination of the Adaptase reaction by a dNTP mixture leads to the formation of synthetic tail sequences of high-complexity base composition to the 3′-end of the random-primed DNA synthesis products. Since this tailing reaction can extend to up to 50 bp (Supplementary Fig. 1b), the long synthetic tail sequences were not sufficiently trimmed by snmC-seq trimming parameters (16 bp from both 5′ and 3′) causing a failure in mapping of reverse reads that retained these synthetic tail sequences. As expected, further trimming from the 5′ end of reverse reads increased their mapping rates (Supplementary Fig. 1c), supporting the high-complexity tailing of library molecules as the cause of the low read mapping rates. We tested the nucleotide carryover hypothesis by supplying different amounts of dNTP (31.25–500 nM) to the random-primed DNA synthesis reaction (Fig. 1b). Reducing dNTP concentration effectively alleviated the aberrant elevation of C frequency at the beginning of reverse reads (Supplementary Fig. 1d), and increased the mapping rate of reverse reads (Fig. 1b). To develop a procedure that robustly prevents dNTP contamination, we incorporated into snmC-seq2 a dephosphorylation step before the Adaptase reaction using a temperature-sensitive shrimp alkaline phosphatase (SAP) to inactivate nucleotide triphosphates (Fig. 1a). The SAP treatment completely suppressed the aberrant base composition observed in the reverse reads generated by snmC-seq (Fig. 1c). We incorporated the SAP treatment to snmC-seq library preparation (snmC-seq + SAP) from human frontal cortex (Brodmann area 10, BA10) and validated that the dephosphorylation of dNTP significantly improved the mapping of reverse reads (Supplementary Fig. 1e and Supplementary Table 1, *p* = 2.4 × 10^–58^, *t*-test). We found that the addition of SAP treatment had no impact on the sequencing library complexity (Supplementary Fig. 1f).

### Reduced artifactual reads in snmC-seq2

**Fig. 2:**
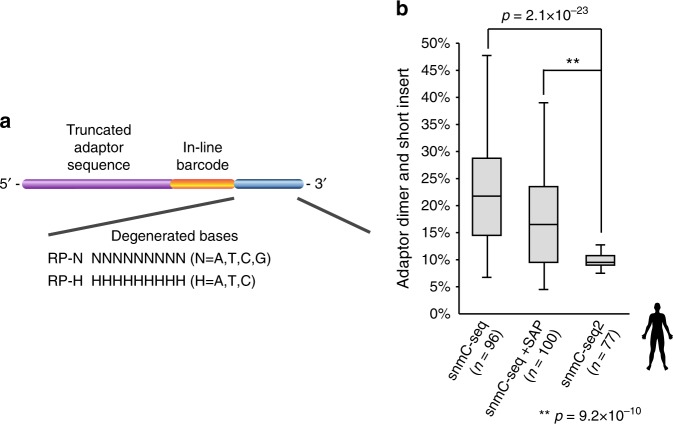
Reduction of adapter dimer and short insert reads by modulating the degenerate 3′ arm of random primers. **a** RP-H and RP-H oligos are used for snmC-seq and snmC-seq2, respectively. **b** Adapter dimer and short insert reads were significantly reduced in snmC-seq2 libraries. The elements of all box-plots are defined as following—center line, median; box limits, first and third quartiles; whiskers, 1.5× interquartile range

Sequencing reads containing adapter dimer sequences are a major type of artifact in snmC-seq libraries^5^. Indeed, libraries generated from human medial frontal gyrus using snmC-seq contained an average of 22.6 ± 9.5% (± indicates standard deviation) adapter dimer sequence or short insert (Fig. 2b). The main source of such artifacts is carryover of unused random primer into the Adaptase reaction. We hypothesized that some unused random primers are present in partially double-stranded form caused by primer-to-primer hybridization and are resistant to exonuclease 1 digestion (step 3 in Fig. 1a). We speculated that adapter dimers could be reduced by removing the guanine (G) base in the degenerated 3′ arm of snmC-seq random primers (Fig. 2a). Random primers used in snmC-seq and snmC-seq2 are hereafter referred to as RP-N (N = A, T, C, G) and RP-H (H = A, T, C), respectively. The use of RP-H was expected to destabilize primer-to-primer hybridization by preventing the formation of more stable G:C pairs. We also expected that the hybridization between RP-H and bisulfite-converted genomic DNA to be minimally affected, since >94% of C is converted to U during bisulfite conversion. To experimentally test the effect of RP-H, we compared snmC-seq libraries generated from medial frontal gyrus as well as snmC-seq + SAP and snmC-seq2 libraries generated from BA10 (Fig. 2b and Supplementary Table 1). Sequencing libraries generated using RP-H contain significantly less adapter dimer reads than those generated with RP-N (*p* = 9.2 × 10^–10^, *t*-test, Fig. 2b).

### Improved throughput for library preparation in snmC-seq2

We have also improved the throughput of single-cell methylome library preparation by developing a 384-well DNA-binding plate for cleaning up bisulfite conversion reactions. Compared to the currently available 96-well silica column plates, our newly designed 384-well DNA-binding plate provided 33% (*p* = 9.2 × 10^–10^, *t*-test) greater library complexity (Supplementary Fig. 2a), suggesting more efficient retention of bisulfite-converted DNA from single cells. An advantage of snmC-seq is the multiplexing of four samples for 3′-adaptor tagging and all subsequent steps (step 4 in Fig. 1a)^5^. Individual samples were indexed using in-line barcodes located upstream of 3′ degenerate arm of random primers (Fig. 2a). Sample multiplexing was enhanced in snmC-seq2 by reducing the reaction volume of random-primed DNA synthesis, which allowed eight reactions to be combined for 3′-adaptor tagging. A fully automated robotic protocol has been developed for snmC-seq2, enabling the preparation of 3072 single-nucleus methylome libraries per experiment. We also developed 768 pairs of library amplification primers containing dual unique indices to avoid index-hopping on patterned flowcells (Supplementary Table 2)^9^. Together with eight in-line barcodes, the primer set allow the multiplexing of 6144 single cells for pooled sequencing. Robotic scripts are provided in Supplementary Software 1.

### snmC-seq2 produces uniformly covered methylome profiles

We tested the snmC-seq2 method by generating single-nucleus methylomes from mouse primary motor cortex (MOp, Supplementary Table 1). Single-nucleus methylome libraries generated for MOp contain markedly reduced (6.1 ± 5.2%) adapter dimer and short insert reads, compared to that of snmC-seq libraries (29.2 ± 16.5%, Table 1 and Supplementary Fig. 2e-g)^5^. Read mapping rate was improved from 52 ± 4% for snmC-seq to 64.7 ± 2.6% for snmC-seq2. In addition, snmC-seq2 shows enhanced library complexity (maximal coverage of 30.8 ± 7.5% of mouse genome) compared to 22.2 ± 5.7% for snmC-seq. Both snmC-seq and snmC-seq2 provided greater library insert size than sc-WGBS, allowing more genomic bases to be sequenced for a given library complexity (Supplementary Fig. 2h). We further examined whether replacing RP-N with RP-H affects the coverage uniformity of snmC-seq2 libraries. Consistent with previous reports^3^, GC-rich regions such as CpG islands (CGI) are enriched in methylomes generated with PBAT-derived strategies, including snmC-seq (Supplementary Fig. 2b). Enrichment of CGI was reduced in snmC-seq2 libraries, which also showed more uniform genome coverage for 1 and 10 kb genomic bins than snmC-seq, comparable to traditional MethylC-seq (Supplementary Fig. 2c).

**Table 1:** Comparison of the quality of methylome libraries generated by snmC-seq, snmC-seq2, and MethylC-seq

Tissue	FC	MOp	Exc
DNA input	1 cell	1 cell	>50 ng
Method	snmC-seq	snmC-seq2	MethylC-seq
Adaptor dimer and short insert	29.2 ± 16.5%	6.1 ± 5.2%	0.45 ± 0.01%
Mapping	52 ± 4%^a^	64.7 ± 2.6^a^	76.4 ± 0.35^b^
Library complexity	22.2 ± 5.7%^c^	30.8 ± 7.5%^c^	N/A

*FC* mouse frontal cortex; *MOp* mouse primary motor cortex; *Exc* Camk2a+-expressing mouse cortical excitatory neurons

^a^Mapping rate computed from both forward and reverse reads

^b^Only forward reads were generated

^c^Coverage of mouse genome

**Fig. 3:**
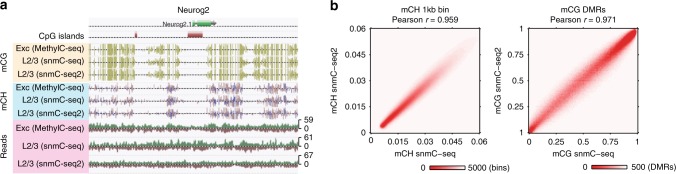
snmC-seq2, snmC-seq, and MethylC-seq generate consistent methylome profiles. **a** Comparison of CG, CH methylation, and read coverage in the region surrounding Neurog2. L2/3 indicates combined methylome profiles of layer 2/3 excitatory neuron clusters. **b** Correlation of CH and CG methylation by snmC-seq2 and snmC-seq were compared for 1 kb genomic bins and CG-DMR regions, respectively. mCG, CG methylation; mCH, CH methylation

To rigorously evaluate the methylome profiles generated by snmC-seq2, we compared mouse frontal cortex profiled with snmC-seq^5^, MOp analyzed with snmC-seq2 along with cortical excitatory neurons analyzed with MethylC-seq^10^. Visual examination revealed consistent CG methylation (mCG) and non-CG or CH methylation (mCH) levels across all three methods, and uniform snmC-seq2 read coverage in the genomic region surrounding *Neurog2* locus (Fig. 3a). Genome-wide coverage analysis showed that CG sites are more uniformly covered in snmC-seq2 (13.4 ± 5.79×) than MethylC-seq (12.4 ± 7.6×) and snmC-seq (15.2 ± 9.0×) (Supplementary Fig. 2d). Further analyses revealed strong correlations between snmC-seq2 and snmC-seq for the quantification of mCH at 1 kb genomic bins (Fig. 3b, Pearson *r* = 0.959, *p* < 2.2 × 10^–308^). For all genomic bin sizes analyzed, correlations between snmC-seq2 and snmC-seq were comparable to those between two biological replicates of excitatory neuron methylomes generated with MethylC-seq (Supplementary Fig. 3). Similarly, mCG levels measured with snmC-seq2 and snmC-seq were strongly correlated at differentially methylated regions (Fig. 3b, Pearson *r* = 0.971, *p* < 2.2 × 10^–308^) identified in our previous study across all mouse cortical neuron types^5^.

## Discussion

In this study, we introduce snmC-seq2, a method for single-nucleus methylome library preparation providing significant improvements in virtually all pertinent aspects, including read mapping, amount of artifactual reads, throughput, library complexity, and coverage uniformity. The development of snmC-seq2 significantly narrows the gap of sequencing library quality between single-cell and bulk methylomes and provides a compelling strategy to generate large-scale single-cell methylome datasets for the survey of epigenomic diversity across human body cell types and dynamic cellular processes.

## Methods

A detailed bench protocol is provided in the Supplementary Methods.

### Animal procedures and fluorescence-activated cell sorting isolation of single nuclei

Male C57Bl/6J mice were purchased from Jackson laboratories at 8 weeks of age and maintained in our facility for 48 h before dissection. Animals were maintained in the Salk animal barrier facility on 12 h dark-light cycles with food ad-libitum. Brains were extracted and sliced coronally at 600 µm from the frontal pole across the whole brain in ice-cold dissection buffer containing 2.5 mM KCl, 0.5 mM CaCl_2_, 7 mM MgCl_2_, 1.25 mM NaH_2_PO_4_, 110 mM sucrose, 10 mM glucose, and 25 mM NaHCO_3_. The solution was kept ice-cold and bubbled with 95%O_2_/5%CO_2_ for at least 15 min before starting the slicing procedure. Slices were kept in 12-well plates containing ice-cold dissection buffer until dissection (normally for approximately 20 min) using a SZX16 Olympus microscope equipped with an SDF PLAPO 1XPF objective. The MOp region was manually dissected from two 600 µm consecutive coronal sections at the following bregma coordinates: AP 2.36–1.16 mm; L 1.00–2.75 mm. Only cortical tissue was dissected and snap-frozen in dry ice. Tissue from 15 males for each of two biological replicas was processed for nuclei preparation as previously described^5,11^. Single nuclei were sorted into 384-well PCR plates containing 2 µL digestion buffer per well. A volume of 20 mL digestion buffer contains 10 mL M-digestion buffer (2×, Zymo D5021-9), 1 ml Proteinase K (20 mg, Zymo D3001-2-20), 9 mL water, and 10 µL unmethylated lambda DNA (100 pg/µL, Promega, D1521). All protocols were approved by the Salk Institute’s Institutional Animal Care and Use Committee.

### Human brain tissues

Postmortem human brain biospecimens were obtained from brain donors of the University of Miami Brain Endowment Bank. snmC-seq with SAP treatment (snmC-seq + SAP) was applied to prefrontal cortex (BA10) tissues obtained from a 58-year-old Caucasian male with a postmortem interval (PMI) = 23.4 h. snmC-seq2 was applied to BA10 cortical tissue of a 25-year-old Caucasian male with a PMI = 20.8 h. Both donors were confirmed to have no known neuropathological changes by an neuropathologist.

### Bisulfite conversion

Fifteen microliters of CT conversion reagent (Zymo D5003-1) was added to each well of the 384-well plate. The plates were incubated for 98 °C for 8 min, then 64 °C for 3.5 h followed by 4 °C. All subsequent centrifugation steps were performed for 5 min at 5000 × *g*. Eighty microliters of M-Binding Buffer (Zymo D5006-3) was added to Zymo-Spin 384 Well Plate (Zymo C2012), and bisulfite-converted samples were transferred to these plates and mixed by pipetting, and centrifuged. One hundred microliters of M-Wash Buffer (Zymo D5040-4) was added and the plates were centrifuged. Fifty microliters of M-Desulphonation Buffer (Zymo D5040-5) was added and the plates were incubated at room temperature for 15 min, then centrifuged. The plates were washed twice with 100 µL M-Wash Buffer, then eluted into a clean 384-well plate with 7 µL EB Buffer (Qiagen 19086) containing 500 nM RP-N or RP-H primers (primer sequences provided in Supplementary Methods).

### snmC-seq2 library preparation

Bisulfite-converted samples were denatured at 95 °C for 3 min, then placed on ice for 2 min. Five microliters of Random Priming master mix [1 µL 10× Blue Buffer (Enzymatics B0110), 0.25 µL Klenow Exo- (50U/µL, Enzymatics, P7010-HC-L), 0.5 µL dNTP (10 mM each, NEB N0447L), 3.25 µL water] was added and incubated at 4 °C for 5 min, 25 °C for 5 min, and 37 °C for 60 min followed by 4 °C. A volume of 1.5 µL enzyme mix containing 1 µL Exonuclease 1 (20 U/µL, Enzymatics X8010L) and 0.5 µL rSAP (1 U/µL, NEB M0371L) was added, then incubated at 37 °C for 30 min followed by 4 °C. 0.8× SPRI beads were added, mixed, and incubated for 5 min at room temperature to allow DNA to bind. The beads were washed three times with 80% ethanol and eluted in 10 µL EB buffer (Qiagen 19086). The samples were denatured again at 95 °C for 3 min, and 10.5 µL Adaptase master mix [2 µL G1, 2 µL G2, 1.25 µL G3, 0.5 µL G4, and 0.5 µL G5 (Swift Biosciences 33096)] was added and incubated at 37 °C for 30 min, then 95 °C for 2 min. Twenty-five microliters of 2× KAPA HiFi HotStart ReadyMix (Kapa, KK2602) and 5 µL custom indexing primer mix (6 µM P5 and 10 µM P7) were added (primer sequences provided in Supplementary Table 2). The PCR was programmed as follows: (1) 95 °C for 2 min; (2) 98 °C for 30 s; (3) 98 °C for 15 s; (4) 64 °C for 30 s, (5) 72 °C for 2 min, (6) 72 °C for 5 min; and (7) 4 °C hold. Repeat steps 3–5 for 15 total cycles. PCR reactions were cleaned with 0.8× SPRI beads for three rounds. Library concentration was determined with Qubit™ dsDNA BR Assay Kit (ThermoFisher Q32853). Libraries were sequenced using Illumina Novaseq instrument.

### Bioinformatics

Sequencing read mapping, quality filtering and the summary of DNA methylation level for each cytosine was performed as previously described with minor modifications^5^. Non-clonal mapped reads were filtered for MAPQ > 10 using samtools view -bq10 option. MethylC-seq data generated from pan-excitatory mouse cortical neurons were used for the comparison with snmC-seq and snmC-seq2 datasets (GSM1541958, GSM1541959)^10^. Methylome profiles generated by snmC-seq or snmC-seq2 for Layer 2/3 excitatory neurons were aggregated for the comparison with MethylC-seq data. Layer 2/3 excitatory neurons in the snmC-seq dataset was annotated in our previous study^5^.

To identify layer 2/3 excitatory neurons in snmC-seq2 dataset generated for mouse primary motor cortex, we computed CH methylation ratio of each non-overlapping 100 kb bins across the genome, defined as the number of methylated basecalls divided by the number of total basecalls in the bin with CH context. We only included 18 893 bins with >100 total basecalls in more than 97.5% cells in the downstream analysis. Bin-level methylation ratios were divided by the global mCH level of each cell analyzed. We performed principal component analysis on the cell-by-bin methylation ratio matrix and use the top 150 principal components for hierarchical clustering. The expression level of a gene is known to show anti-correlation with gene body mCH level^10,11^, we therefore assigned the clusters to putative cell types based on the gene body mCH level of the established marker genes. Layer 2/3 excitatory neurons were identified as the cell cluster with low mCH level at *Cux1* (marker of layer 2/3 and L4) and Satb2 (marker of excitatory neurons) and high mCH level at *Rorb* (marker of L4 and L5a). The identified L23 neurons are robust to different number of principal components used for clustering.

Preseq was used to estimate library complexity using forward reads with Preseq gc_extrap function with options -e 5e + 09 -s 1e + 07^12^. Library complexity values shown in this study were estimated for the sequencing depth of 50 million read pairs.

### Code availability

Computer codes used for processing snmC-seq/snmC-seq2 data can be downloaded from https://github.com/zhoujt1994/snmC-seq2.

## Electronic supplementary material

Supplementary Information

Description of Additional Supplementary Files

Supplementary Software 1

## Data Availability

Raw data and processed data are available from NCBI GEO accession GSE112471. The comparison of DNA methylome generated from mouse cortical excitatory neurons MethylC-seq, snmC-seq, and snmC-seq2 can be visualized at http://neomorph.salk.edu/snmC-seq2.php. All other data available upon reasonable request.

## References

[1] Miura F, Enomoto Y, Dairiki R, Ito T (2012). Amplification-free whole-genome bisulfite sequencing by post-bisulfite adaptor tagging. Nucleic Acids Res..

[2] Smallwood SA (2014). Single-cell genome-wide bisulfite sequencing for assessing epigenetic heterogeneity. Nat. Methods.

[3] Farlik M (2015). Single-cell DNA methylome sequencing and bioinformatic inference of epigenomic cell-state dynamics. Cell Rep..

[4] Zhu P (2018). Single-cell DNA methylome sequencing of human preimplantation embryos. Nat. Genet..

[5] Luo C (2017). Single-cell methylomes identify neuronal subtypes and regulatory elements in mammalian cortex. Science.

[6] Mulqueen RM (2018). Highly scalable generation of DNA methylation profiles in single cells. Nat. Biotechnol..

[7] Lister R (2008). Highly integrated single-base resolution maps of the epigenome in Arabidopsis. Cell.

[8] Lister R (2009). Human DNA methylomes at base resolution show widespread epigenomic differences. Nature.

[9] Sinha R, Stanley G, Gulati GS, Ezran C, Travaglini KJ (2017). Index switching causes ‘spreading-of-signal’ among multiplexed samples in Illumina HiSeq 4000 DNA sequencing. BioRxiv.

[10] Mo A (2015). Epigenomic signatures of neuronal diversity in the mammalian brain. Neuron.

[11] Lister R (2013). Global epigenomic reconfiguration during mammalian brain development. Science.

[12] Daley T, Smith AD (2013). Predicting the molecular complexity of sequencing libraries. Nat. Methods.

