# Supplementary material for: Robust single-cell DNA methylome profiling with snmC-seq2
Source: Nat Commun. 2018 Sep 20;9:3824. doi: 10.1038/s41467-018-06355-2 (PMC6147798; doi:10.1038/s41467-018-06355-2)
Supplement: Supplementary file 1 — Supplementary Information [file 41467_2018_6355_MOESM1_ESM.pdf]

Supplementary Information for

Robust single-cell DNA methylome profiling with snmC-seq2

Luo et al.

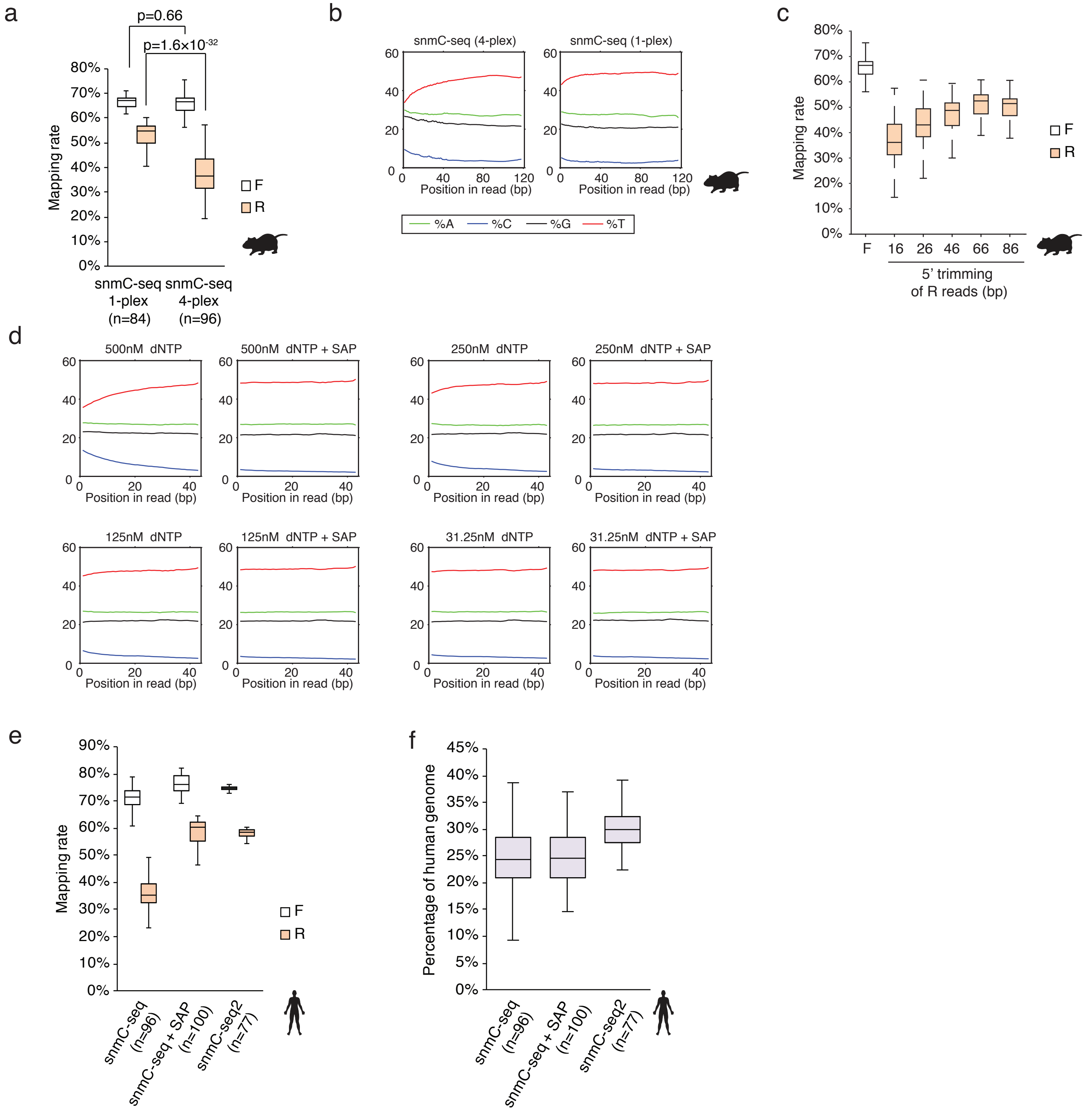

Supplementary Figure 1. (a) Multiplexed (4-plex) snmC-seq shows reduced reverse reads (R) mapping compared to non-multiplexed snmC-seq (1-plex). (b) Multiplexed snmC-seq shows aberrant reverse reads base composition. (c) Trimming of 5' end increases the mapping rate of reverse reads (n=94). (d) Reducing dNTP concentration in random-primed DNA synthesis or treatment with Shrimp Alkaline Phosphatase (SAP) suppresses the aberrant reverse reads base composition. (e) SAP treatment increases the mapping rate of reverse reads for single-cell methylomes generated from human cortical tissues. (f) Comparison of library complexity for libraries generated with snmC-seq, snmC-seq with SAP treatment (snmC-seq + SAP) and snmC-seq2 using single nuclei isolated from human cortical tissues. The elements of all box-plots are defined as following - center line, median; box limits, first and third quartiles; whiskers, 1.5x interquartile range.

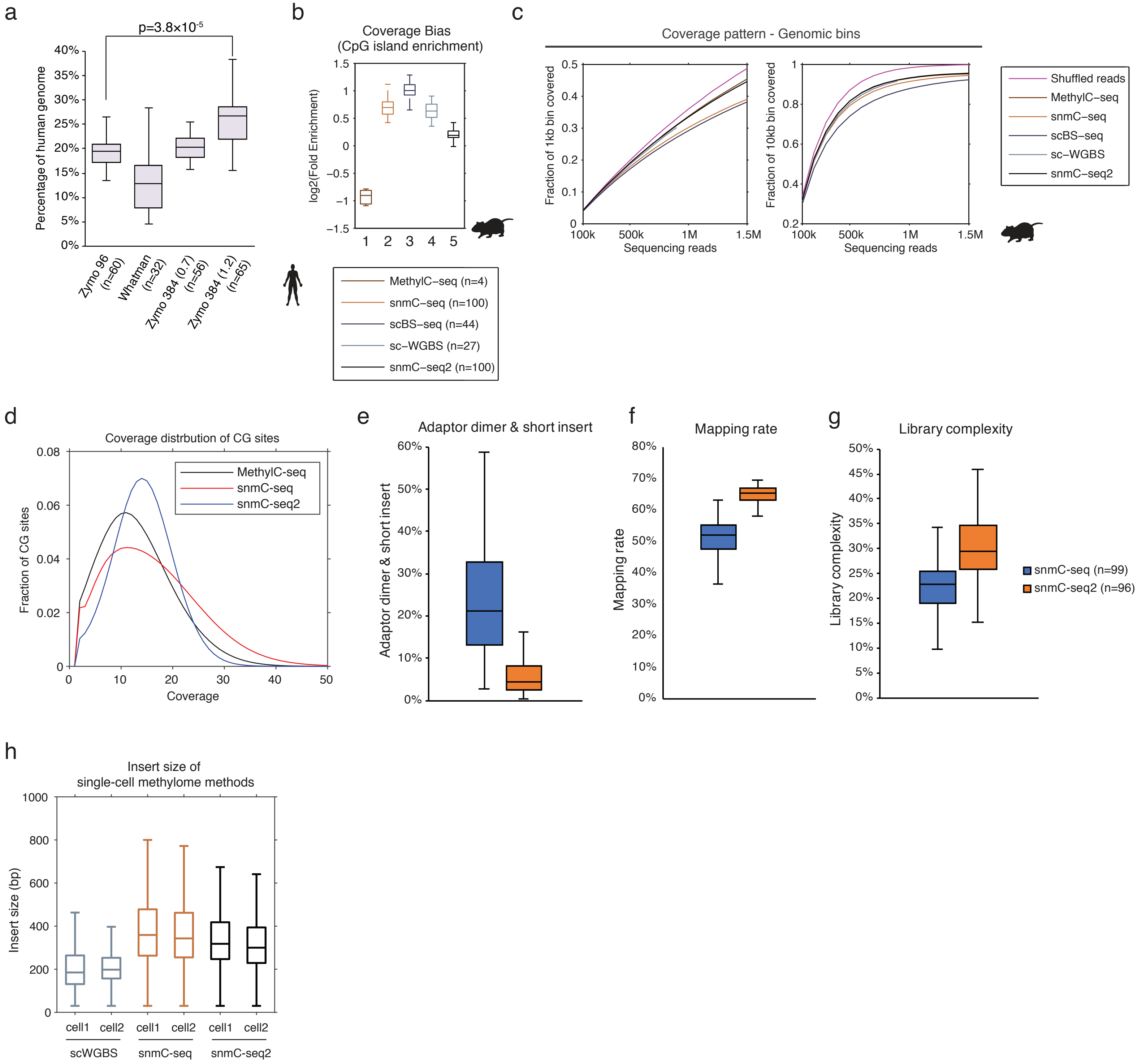

Supplementary Figure 2. (a) Library complexity for single cell methylomes generated using different DNA binding column plates. Zymo 96 - Zymo-Spin I-96 Binding Plates (Zymo C2004). Whatman - 384 Wells Unifilter Microplate (Whatman 7700-2110). Zymo 384 (0.7) - 384-well DNA binding column plates with glass fiber pore size 0.7 μm. Zymo 384 (1.2) - 384-well DNA binding column plates with glass fiber pore size 1.2 μm.
(b) Enrichment of CpG islands in DNA methylome generated by traditional MethylC-seq1, snmC-seq2, scBS-seq3, sc-WGBS4 and snmC-seq2. (c) Fraction of 1kb and 10kb non-overlapping bins covered by single cell methylome reads as a function of the sequencing depth. (d) Coverage distribution of genome-wide CG sites shows snmC-seq2 has more uniform coverage than MethylC-seq and snmC-seq. (e-g) Comparison of the quality of methylome libraries generated by snmC-seq, snmC-seq2. (h) Library insert size distribution for scWGBS, snmC-seq and snmC-seq2. The elements of all box-plots are defined as following - center line, median; box limits, first and third quartiles; whiskers, 1.5x interquartile range.

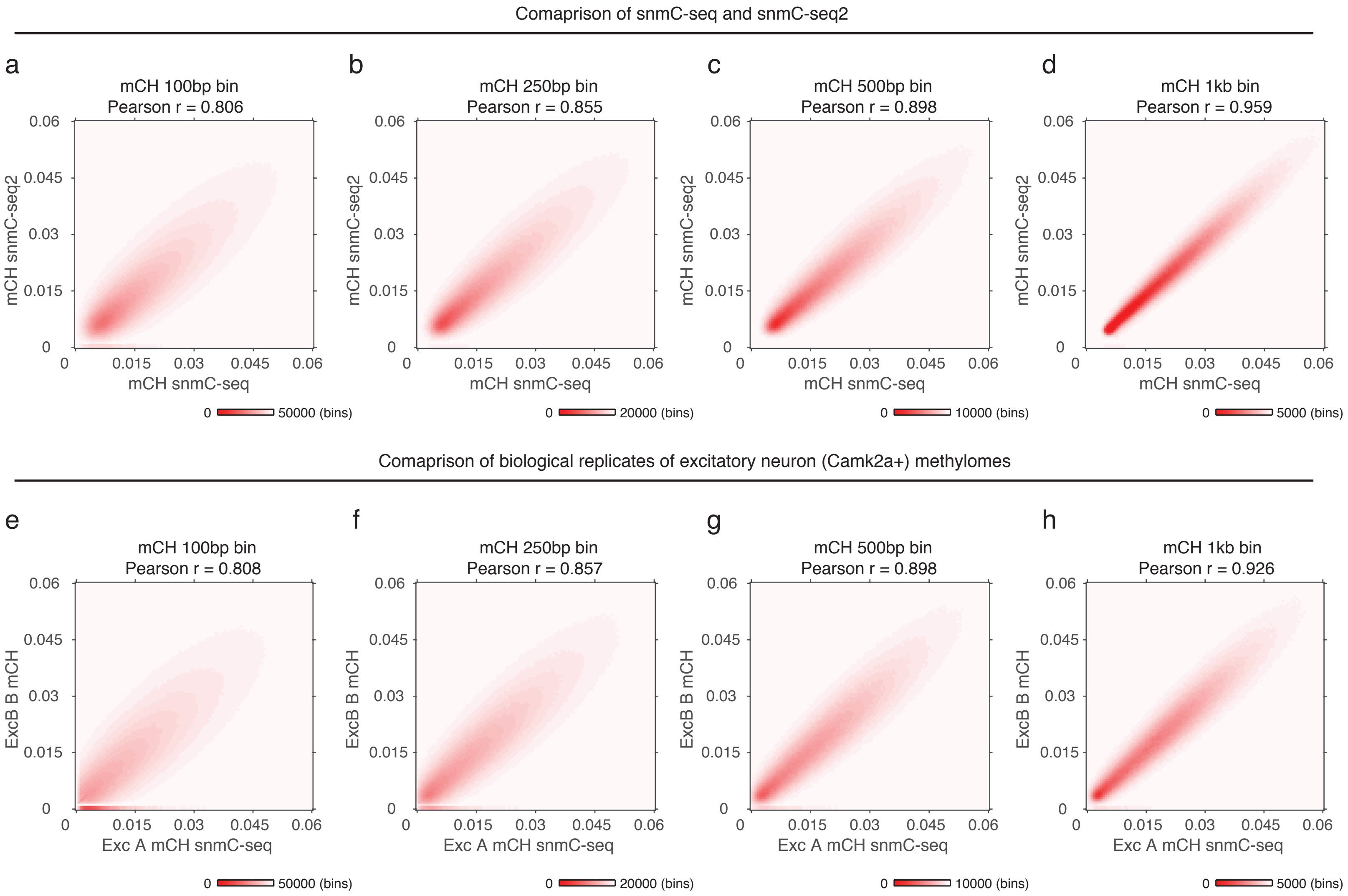

Supplementary Figure 3. (a-d) Correlation of CH methylation by snmC-seq2 and snmC-seq were compared for 100bp, 250bp, 500bp and 1kb genomic bins. (e-h) Correlation of biological replicates of excitatory neuron methylomes (Exc) were compared for 100bp, 250bp, 500bp and 1kb genomic bins.

Supplementary Methods - snmC-seq2 protocol

Protocol to bisulfite convert and generate 3,072 single-cell methylome libraries in one experiment (8x 384-well plates) from mouse or human frozen brain tissue.

Materials

General reagents

- FACS sorted single-nuclei into 384 well PCR plate
- HyClone™ HyPure™ Molecular Biology Grade (MB) Water (GE Life Sci. cat. no. SH30538.03)
- 200-Proof (100%) Ethanol (Koptec cat. no. V1001)

Collection of single nuclei by Fluorescence-activated Cell Sorting (FACS)

- M-Digestion Buffer (Zymo cat. no. D5021-9)
- Proteinase K (Zymo cat. no. D3001-2-D)
- Proteinase K Storage Buffer (Zymo cat. no. D3001-2-B)
- Unmethylated Lambda DNA (Promega cat. no. D1521, 100pg/μL)

Bisulfite conversion

- CT Conversion Reagent (Zymo cat. no. D5003-1)
- M-Solubilization Buffer (Zymo cat. no. D5021-7)
- M-Dilution Buffer-Gold (Zymo cat. no. D5006-2)
- M-Reaction Buffer (Zymo cat. no. D5021-8)
- M-Binding Buffer (Zymo cat. no. D5021-7)
- M-Wash Buffer (Zymo cat. no. D5040-4)
- M-Desulphonation Buffer (Zymo cat. no. D5040-5)
- M-Elution Buffer (Zymo cat. no. D5007-6)

Random primers

- HPLC purified Random primers were ordered from Integrated DNA Technologies (IDT)
  P5L_AD002_H
  /5SpC3/TTCCCTACACGACGCTCTTCCGATCTCGATGT(H1:33340033)(H1)(H1)(H1)(H1)(H1)(H1)(H1)(H1)
  P5L_AD006_H
  /5SpC3/TTCCCTACACGACGCTCTTCCGATCTGCCAAT(H1:33340033)(H1)(H1)(H1)(H1)(H1)(H1)(H1)(H1)
  P5L_AD008_H
  /5SpC3/TTCCCTACACGACGCTCTTCCGATCTACTTGA(H1:33340033)(H1)(H1)(H1)(H1)(H1)(H1)(H1)(H1)

P5L_AD010_H
/5SpC3/TTCCCTACACGACGCTCTTCCGATCTTAGCTT(H1:33340033)(H1)(H1)(H1)(H1)(H1)(H1)(H1)(H1)
P5L_AD001_H
/5SpC3/TTCCCTACACGACGCTCTTCCGATCTATCACG(H1:33340033)(H1)(H1)(H1)(H1)(H1)(H1)(H1)(H1)
P5L_AD004_H
/5SpC3/TTCCCTACACGACGCTCTTCCGATCTTGACCA(H1:33340033)(H1)(H1)(H1)(H1)(H1)(H1)(H1)(H1)
P5L_AD007_H
/5SpC3/TTCCCTACACGACGCTCTTCCGATCTCAGATC(H1:33340033)(H1)(H1)(H1)(H1)(H1)(H1)(H1)(H1)
P5L_AD012_H
/5SpC3/TTCCCTACACGACGCTCTTCCGATCTCTTGTA(H1:33340033)(H1)(H1)(H1)(H1)(H1)(H1)(H1)(H1)

Sera-Mag Solid Phase Reversible Immobilization (SPRI) beads

- Sera-Mag SpeedBeads Magnetic Carboxylate Modified (GE Healthcare cat. no. 45152105050250)
- Poly(ethylene glycol) PEG 8000 (Sigma cat no. 89510-250G-F)
- TE buffer pH=8.0 (Ambion cat. no. AM9858)
- 5M NaCl
- 1M Tris-HCl pH=8.0
- 0.5M EDTA pH=8.0
- AMPure XP beads (Beckman Coulter cat no. A63881)
- 100 bp DNA ladder (New England Biolabs cat no. N3231L)

snmC-seq2 library preparation

- Blue Buffer (10x) (Enzymatics cat. no. P7010-HC-L)
- Klenow Exo- (50U/µL) (Enzymatics cat. no. P7010-HC-L)
- Deoxynucleotide Solution Mix (10mM each dNTP) (NEB cat. no. N0447L)
- Exonuclease I (20U/µL) (Enzymatics cat. no. X8010L)
- Shrimp Alkaline Phosphatase (rSAP) (1U/µL) (NEB cat. no. M0371L)
- Sera-Mag SPRI beads
- M-Elution Buffer (Zymo cat. no. 5007-6)
- EB buffer (Qiagen cat. no. 19086)
- Accel-NGS® Adaptase™ Module (Swift Bio cat. no. 330384)
- P5 Indexing Primer (IDT custom DNA oligo, standard desalted)
- P7 Indexing Primer (IDT custom DNA oligo, standard desalted)

- KAPA HiFi HS RM (Kapa cat. no. KK2602)
- Qubit dsDNA BR Assay Kit (Thermo Fisher cat. no. Q32850)

Equipment

- 384-Well Hardshell PCR Plate Clear, 20 Pcs/pk (Thermo Fisher cat. no. 4483285)
- 96-Well Hardshell PCR Plate GPLE, 20 Pcs/pk (Thermo Fisher cat. no. 4483348)
- Zymo-Spin 384 Well Plate, 2 pack (Zymo cat. no. C2012)
- 2.0mL 96-well Deep Well Polypropylene Plate, Sterilized (USA-SCI. cat. no. 1896-2110)
- Reservoir Single Well 96 Bottom High Profile, Clear (Axygen cat. no. RES-SW96-HP)
- Reservoir Single Well 96 Bottom Low Profile, Clear (Axygen cat. no. RES-SW96-LP)
- 15mL Centrifuge Tubes (Olympus cat. no. 28-103)
- 50mL Centrifuge Tubes (Olympus cat. no. 28-106)
- 1.5µL Eppendorf Tubes (Thermo Fisher cat. no. 02-681-320)
- 300µL 8-Strip Tubes
- Microamp Clear Adhesive Film, 100pc (Thermo Fisher cat. no. 4306311)
- Microporous Film, -20C to 80C, 50p, sterile (USA-SCI. cat. no. 2920-1010)
- Speedball Deluxe Soft Rubber Brayer, 4 inches (Statesville N.C.)
- 37°C Incubator
- 384-well and 96-well Compatible Thermocycler
- DynaMag™-96 Side Magnet (Thermo Fisher cat. no. 12331D)
- DynaMag™-2 Magnet (Thermo Fisher cat. no. 12321D)
- Allegra 25R Centrifuge with S5700 2x96 Swinging Bucket Rotor (Beckman Coulter cat. no. 368954)
    - All centrifuge steps at 5000x*g* for 5 minutes at room temperature unless otherwise specified
- (Optional) Axygen DITI 50µL STE.FIL. Robotics Tips (Thermo Fisher cat. no. EVF-50-RS)
- (Optional) Axygen DITI 180µL STE.FIL. Robotics Tips (Thermo Fisher cat. no. EVF-180-RS)
- (Optional) TECAN Freedom Evo 100 Base Unit with MultiChannel™ Arm MCA 96
- (Optional/Required for TECAN use) Collection Spacer/Adapter, Type 2, 26.5mm, Te-VacS (Tecan US Inc. cat. no. 10760662)

Reagent Setup

Ethanol, 80%

To 40mL of 200-proof Ethanol, add 10µL MB water in a 50mL tube. Keep solution sealed when not in use. Prepare ~6 tubes fresh before each library preparation.

Digestion Buffer Plates (for FACS sorting)

In a 50mL tube, combine 15mL M-Digestion Buffer (incubate for 10 minutes at 37°C to dissolve precipitate) with 14mL MB water. Resuspend one tube Proteinase K with 1mL Proteinase K Resuspension Buffer and add 1mL to the digestion buffer mix. Add 10µL λ DNA to aid determination of bisulfite conversion efficiency. Vortex to mix. Aliquot 2µL per well to 20 384-well plates to use for FACS sorting of stained nuclei (TECAN script). Plates can be stored at 4C.

(Optional) This step can be automated using TECAN Freedom Evo 100 with script - *2uL_Digestion_Buffer.*

Sera-Mag Solid Phase Reversible Immobilization (SPRI) Beads

1. Mix Sera-Mag SpeedBeads and transfer 1mL to a 1.5ml tube.
2. Place SpeedBeads on a magnetic stand until clears and carefully remove the supernatant. Wash the beads twice with 1ml of TE. For each wash, remove the tube from the magnet and mix by inversions. Resuspend the washed beads in 1ml of TE.
3. Add 9g of PEG 8000 to a new 50ml sterile conical tube.
4. Add 10ml of 5M NaCl to the 50ml tube.
5. Add 500µl of 1M Tris-HCl pH=8.0 and 100ul of 0.5M EDTA pH=8.0 to the 50ml tube.
6. Mix until all dissolves into solution.
7. Add 1ml of resuspended SpeedBeads to the 50ml tube and fill the volume with MB water.
8. Test against AMPure XP beads using 100bp DNA ladder.

CT Conversion Reagent

Add 7.9 mL M-Solubilization Buffer and 3 mL M-Dilution Buffer to a bottle of CT Conversion Reagent. Shake vigorously at room temperature to fully dissolve before adding 1.6 mL M-Reaction Buffer.

M-Wash Buffer

Add 288mL 200-proof Ethanol to four bottles of M-Wash buffer. Invert to mix. Make fresh each time. Extra buffer can be stored at room temperature.

Random Primer solution

35μL random primer stock (100μM) in 7mL M-Elution buffer (500nM final primer concentration). Aliquot to eight 96-well plates.

Random Priming Master Mix

To have sufficient reagents for robotic preparation of 3,072 reactions, prepare 3600 reactions of Random Priming Master Mix in a 15mL tube and vortex to mix. Aliquot 180μL of master mix into each well of one 96-well plate. Keep sealed on ice until use. Do not store.

Reagent	Vol. per reaction	Vol. for 3,600 reactions
Blue Buffer (10x)	1 μL	3600 μL
Klenow exo- (50U/μL)	0.25 μL	900 μL
dNTP (10mM each)	0.5 μL	1800 μL
MB water	3.25 μL	11700 μL

Exo/rSAP Master Mix

To have sufficient reagents for robotic preparation of 3,072 reactions, prepare 3600 reactions of Exo/SAP Master Mix in a 15mL tube and vortex to mix. Aliquot 53μL master mix to each well of one 96-well plate. Keep sealed on ice until use. Do not store.

Reagent	Vol. per reaction	Vol. for 3,600 reactions
Exonuclease 1 (20U/uL)	1 uL	3600 uL
rSAP (1U/uL)	0.5 uL	1800 uL

Adaptase Master Mix (using Accel-NGS® Adaptase™ Module)

To have sufficient reagents for robotic preparation of 3,072 reactions, prepare 3600 reactions of Adaptase Master Mix in a 15mL tube and vortex to mix. Aliquot 48 uL master mix to each well of on 96-well plate. Keep sealed on ice until use. Do not store.

Reagent	Vol. per reaction	Vol. for 3,600 reactions
EB	4.25 uL	1912.5 uL
Buffer G1	2 uL	900 uL
Reagent G2	2 uL	900 uL
Reagent G3	1.25 uL	562.5 uL
Enzyme G4	0.5 uL	225 uL
Enzyme G5	0.5 uL	225 uL

PCR Primer Mix

Sequences of indexing primers with unique dual barcodes are provided in Supplementary Table 2. Each PCR Primer Mix contains a P5L indexing primer (600 nM) and a P7L indexing primer (1 μM).

Procedure

Bisulfite Conversion

Timing ~ 5h

1. Add 15μL CT conversion reagent to each well of 384-well plate. Pipette up and down for 8 times to mix the sample.

   (Optional) This step can be automated using TECAN Freedom Evo 100 with script - *15uL_Conversion_Buffer*

2. Seal the plate with adhesive film and quick spin for 10s at 2,000x*g* at room temperature. Place the plate in a thermocycler and run the following program:

98°C	8 min
64°C	3.5hrs
4°C	Hold

3. Load 80μL M-Binding buffer to each well of 384-Well DNA Binding Plate.

   (Optional) This step can be automated using TECAN Freedom Evo 100 with script - *80uL_Binding Buffer*

4. Transfer bisulfite conversion reactions to Zymo-Spin 384 Well DNA Binding Plate. Pipette up and down for 8 times to mix the sample. Place the 384-Well DNA Binding Plate on a 2.0mL 96-well Deep Well Plate and centrifuge for 5 min at 5,000g. Discard the flow through by decanting.

   (Optional) This step can be automated using TECAN Freedom Evo 100 with script - *17uL_Conversion_Rxn_To_Binding_Buffer.*

5. Add 100μL M-Wash Buffer to each well of 384-Well DNA Binding Plate. Centrifuge for 5 min at 5,000g and discard the flow through by decanting.

   (Optional) This step can be automated using TECAN Freedom Evo 100 with script - *100uL_M_Wash_Buffer.*

6. Add 50µL M-Desulphonation Buffer to each well of 384-Well DNA Binding Plate. Incubate at room temperature for 15 min. Centrifuge for 5 min at 5,000g and discard the flow through by decanting.

(Optional) This step can be automated using TECAN Freedom Evo 100 with script - *50uL_Desulphonation_Buffer.*

7. Add 100µL M-Wash Buffer to each well of 384-Well DNA Binding Plate. Centrifuge for 5 min at 5,000g and discard the flow through by decanting.

(Optional) This step can be automated using TECAN Freedom Evo 100 with script - *100uL_M_Wash_Buffer.*

8. Repeat step 7.

9. Place 384-Well DNA Binding Plate on new 384-well PCR plate. Add 7µL Random Primer Solution. Each quadrant of 384-well plate is barcoded with a distinct indexed random primer (Fig. 1). Every two 384-well plates receive a complete set of all eight indexed random primers. Incubate for 5 min at room temperature. Centrifuge for 5 min at 5,000g and discard the 384-Well DNA Binding Plate.

(Optional) This step can be automated using TECAN Freedom Evo 100 with script - *7uL_Primer_Elution.*

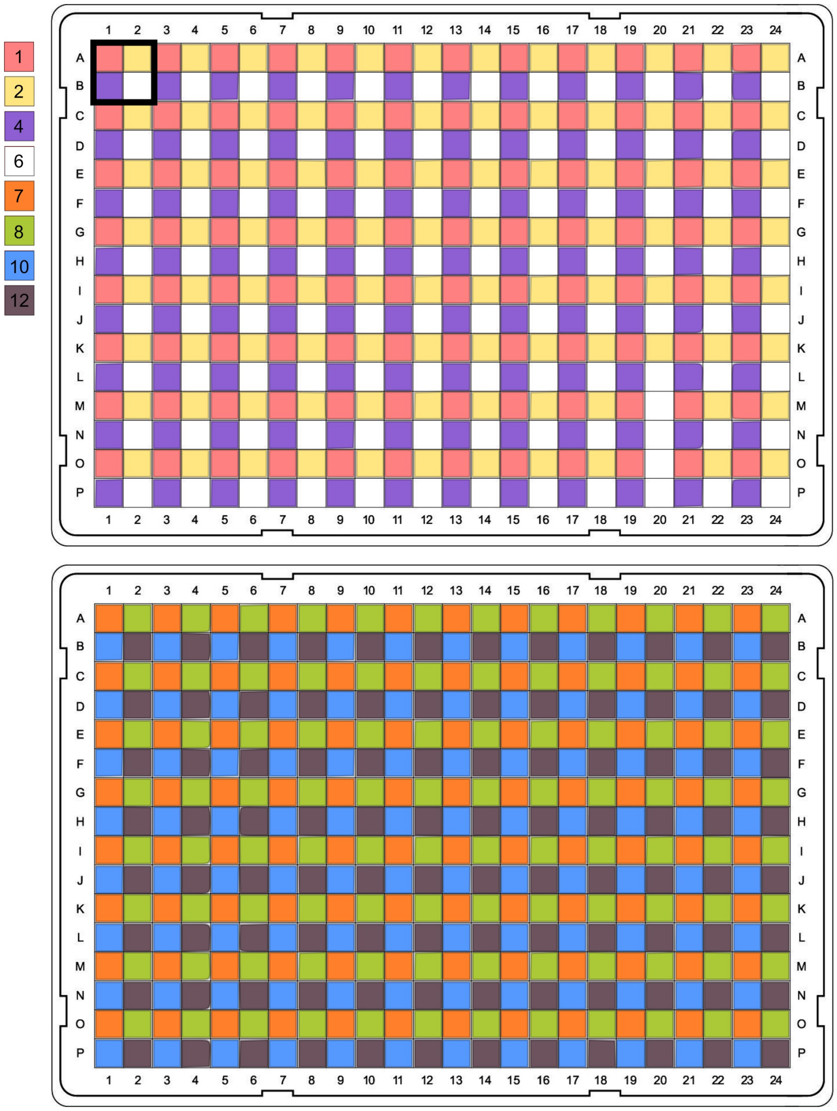

Figure 1. Schematics of single-cell barcoding with indexed random primers. Using a TECAN Freedom Evo 100 with MultiChannel Arm MCA 96, each indexed random primer is added into a quadrant of 384-well DNA Binding Plate. The color of each quadrant indicates the random primer barcode.

10. Seal the plate with adhesive film and store at -20°C for up to 1 week.

Random-primed DNA synthesis

Timing ~2h

1. Denature the samples by placing 384-well PCR plate on a thermocycler and run the following program.

   95°C 3 min

Immediately place the plate on ice for 2 minutes.

2. Add 5µL Random Priming Master Mix to each well of the 384-well PCR plate. Vortex and quick spin for 10s at 2,000x*g*.

   (Optional) This step can be automated using TECAN Freedom Evo 100 with script - *5uL Random_Priming_Mix.*

3. Place the plate in a thermocycler and run the following program:

4°C	5 min
25°C	5 min
37°C	60 min
4°C	Hold

Inactivation of free primers & dNTP

4. Add 1.5µL Exo/rSAP Master Mix to each well of the 384-well PCR plate. Vortex to mix the samples and quick spin for 10s at 2,000x*g*.

   (Optional) This step can be automated using TECAN Freedom Evo 100 with script - *1uL_Exo_rSAP_MM.*

5. Place the plate in a thermocycler and run the following program

37°C	30 min
4°C	Hold

Sample clean-up

6. Add 73.6µL (0.8x) SPRI Beads to each well of four clean 96-well PCR plates.

   (Optional) This step can be automated using TECAN Freedom Evo 100 with script - *BP_Add_To_Clean_96well.*

7. Transfer samples from two 384-well plates to each 96-well plate. The eight quadrants (from two 384-well plates) barcoded with distinct indexed random primers are combined. Mix the samples by vortexing and incubate for 5 minutes at room temperature, then quick spin for 10s at 2,000x*g.*

   (Optional) This step can be automated using TECAN Freedom Evo 100 with script - *BP_Transfer_Lib_384well_To_96well.*

8. Place 96-well plates on DynaMag™-96 Side Magnet, let stand until solution in wells is clear of beads (~5 minutes). Remove supernatant and wash beads 3x with 150µL fresh 80% EtOH. Remove all EtOH, remove plates from magnet, and let beads dry at room temperature. DO NOT overdry beads.

   (Optional) This step can be automated using TECAN Freedom Evo 100 with script - *BP_2Plate_150uL_EtOH_Wash.*

9. Add 10µL EB buffer and resuspend beads by pipet. Vortex and incubate for 5 minutes at room temperature, then quick spin for 10s at 2,000x*g.* Place back on magnet and let stand until solution is clear of beads (~5 minutes).

10. Remove 10uL supernatant to a clean 96-well PCR plate.

    (Optional) This step can be automated using TECAN Freedom Evo 100 with script - *10uL_96Well_Elution.*

Adaptase reaction

11. Denature the samples by placing 96-well plates on a thermocycler and run the following program.

    95°C 3 min

    Immediately place the plate on ice for 2 minutes.

12. Add 10.5µL Adaptase Master Mix to each well of the 96-well PCR plate. Vortex and quick spin for 10s at 2,000x*g*.

    (Optional) This step can be automated using TECAN Freedom Evo 100 with script - *10uL_25uL_Adaptase_KAPA. Enter 10.5uL for the volume transferred.*

13. Place the plate in a thermocycler and run the following program:

    37°C 30 min
    95°C 2 min
    4°C Hold

Library amplification

14. Add 5uL PCR Primer Mix.

    (Optional) This step can be automated using TECAN Freedom Evo 100 with script - *5uL_PCR_Primer.*

15. Add 25µL 2x KAPA HiFi Mix. Vortex and quick spin for 10s at 2,000x*g*.

    (Optional) This step can be automated using TECAN Freedom Evo 100 with script - *10uL_25uL_Adaptase_KAPA. Enter 25uL for the volume transferred.*

16. Place the plate in a thermocycler and run the following program:

    a. 95°C 2 min
    b. 98°C 30s
    c. 98°C 15s
    d. 64°C 30s
    e. 72°C 2 min
       Go to step c 14 times
    f. 72°C 5 min
    g. 4°C Hold

<u>*Library clean-up*</u>

17. *Sample cleanup.* Add 40µL (0.8x) SPRI Beads to each well of four 96-well PCR plates. Transfer contents of one 96-well plate to another to combine four 96-well plates to two 96-well plates. Vortex and incubate for 5 minutes at room temperature, then quick spin for 10s at 2,000x*g.*

    (Optional) This step can be automated using TECAN Freedom Evo 100 with script - *BP_Add_Beads_And_Combine_96well.*

18. Place 96-well plates on DynaMag™-96 Side Magnet, let stand until solution in wells is clear of beads (~5 minutes). Remove supernatant and wash beads 2x with 150µL fresh 80% EtOH. Remove all EtOH, remove plate from magnet and let beads dry at room temperature. DO NOT overdry beads.

    (Optional) This step can be automated using TECAN Freedom Evo 100 with script - *BP_2Plate_150uL_EtOH_Wash (stop script after two wash steps).*

19. Add 25µL EB and resuspend beads by pipet. Vortex and incubate for 5 minutes at room temperature, then quick spin for 10s at 2,000x*g.* Place back on magnet and let stand until solution is clear of beads (~5 minutes). Remove 25uL supernatant to a clean 96-well PCR plate.

(Optional) This step can be automated using TECAN Freedom Evo 100 with script - *20uL_96Well_Elution.*

20. Add 20µL (0.8x) SPRI Beads to each well of two 96-well PCR plates. Transfer contents of one 96-well plate to another to combine two 96-well plates to one 96-well plate. Vortex and incubate for 5 minutes at room temperature, then quick spin for 10s at 2,000x*g.*

    (Optional) This step can be automated using TECAN Freedom Evo 100 with script - *BP_Add_Beads_And_Combine_96well.*

21. Place 96-well plate on DynaMag™-96 Side Magnet, let stand until solution in wells is clear of beads (~5 minutes). Remove supernatant and wash beads 2x with 150µL fresh 80% EtOH. Remove all EtOH, remove plate from magnet, and let beads dry at room temperature. DO NOT overdry beads.

    (Optional) This step can be automated using TECAN Freedom Evo 100 with script - *BP_1Plate_150uL_EtOH_Wash.*

22. Add 20µL EB buffer and resuspend beads by pipet. Vortex and incubate for 5 minutes at room temperature, then quick spin for 10s at 2,000x*g.* Place back on magnet and let stand until solution is clear of beads (~5 minutes). Combine 20µL eluent from all wells in each column of the 96-well plate (8 wells per column, 12 columns) into 12 1.5µL Eppendorf tubes.
23. Add 128µL (0.8x) SPRI Beads to each 1.5µL Eppendorf tube. Pipette to mix and incubate for 5 minutes at room temperature.
24. Place 1.5µL tubes on DynaMag™-2 Magnet, let stand until solution in tubes in clear of beads (~5 minutes). Remove supernatant and wash beads 2x with 200uL fresh 80% EtOH. Remove all EtOH, remove tubes from magnet, and let beads dry at room temperature. DO NOT overdry beads.
25. Add 40µL EB and resuspend beads by pipet. Incubate for 5 minutes at room temperature. Place tubes back on magnet and let stand until solution is clear of beads (~5 minutes). Remove 40uL supernatant to 12 clean 1.5µL Eppendorf tubes.
26. Measure concentration of each 1.5µL Eppendorf tube with Qubit dsDNA BR Assay Kit. Normalize library concentrations and pool for sequencing.

Supplementary Table 1. Metadata for single-cell methylomes generated with snmC-seq + SAP and snmC-seq2

Sample	Species	Brain area	Labeling	Bisulfite conversion method	Library type	Sequencer	Total reads	Mapped reads	% Mapped reads	% Mapped forward reads	% Mapped reverse reads	Non-clonal reads	% Non-clonal reads	Filtered reads (MAPQ > 10)	% Filtered reads (MAPQ > 10)	mCCC/CCC	mCG/CG	mCH/CH	Estimated mCG/CG	Estimated mCH/CH	Coverage (%)
Pool_1681_AD010_indexed	Homo sapiens	BA10	NeuN+	Zymo EZ-96 direct	snmC-seq + SAP	Illumina HiSeq 4000	2919272	1858394	63.7%	71.5%	55.8%	1383602	74.5%	1124120	81.2%	0.00653	0.76234	0.02085	0.76078	0.01441	3.73
Pool_1686_AD006_indexed	Homo sapiens	BA10	NeuN+	Zymo EZ-96 direct	snmC-seq + SAP	Illumina HiSeq 4000	3931354	2441886	62.1%	68.2%	56.0%	1654010	67.7%	1341801	81.1%	0.01461	0.80288	0.06231	0.79996	0.04841	4.29
Pool_1693_AD008_indexed	Homo sapiens	BA10	NeuN+	Zymo EZ-96 direct	snmC-seq + SAP	Illumina HiSeq 4000	5763326	3878686	67.3%	75.0%	59.6%	2481130	64.0%	2057507	82.9%	0.00596	0.76821	0.02088	0.76682	0.01501	6.57
Pool_1693_AD010_indexed	Homo sapiens	BA10	NeuN+	Zymo EZ-96 direct	snmC-seq + SAP	Illumina HiSeq 4000	6074344	4122641	67.9%	75.0%	60.8%	2589777	62.8%	2156456	83.3%	0.00863	0.79141	0.04167	0.78959	0.03333	6.9
Pool_1696_AD002_indexed	Homo sapiens	BA10	NeuN+	Zymo EZ-96 direct	snmC-seq + SAP	Illumina HiSeq 4000	5296394	3540255	66.8%	74.8%	58.9%	2140229	60.5%	1747731	81.7%	0.02239	0.81634	0.09219	0.81213	0.0714	5.59
Pool_1699_AD008_indexed	Homo sapiens	BA10	NeuN+	Zymo EZ-96 direct	snmC-seq + SAP	Illumina HiSeq 4000	5028476	3297281	65.6%	74.5%	56.7%	2307725	70.0%	1893974	82.1%	0.00903	0.78597	0.04279	0.78402	0.03407	6.28
Pool_1710_AD008_indexed	Homo sapiens	BA10	NeuN+	Zymo EZ-96 direct	snmC-seq + SAP	Illumina HiSeq 4000	5911880	3915349	66.2%	74.4%	58.0%	2659019	67.9%	2173473	81.7%	0.00544	0.75675	0.00991	0.75542	0.00449	7
Pool_1718_AD006_indexed	Homo sapiens	BA10	NeuN+	Zymo EZ-96 direct	snmC-seq + SAP	Illumina HiSeq 4000	5132844	3322606	64.7%	74.0%	55.5%	2462086	74.1%	2000775	81.3%	0.00744	0.76399	0.01861	0.76222	0.01125	6.73
Pool_1724_AD010_indexed	Homo sapiens	BA10	NeuN+	Zymo EZ-96 direct	snmC-seq + SAP	Illumina HiSeq 4000	3758418	2366395	63.0%	73.7%	52.2%	1793430	75.8%	1446430	80.7%	0.00544	0.75286	0.00932	0.75151	0.0039	4.93
Pool_1729_AD010_indexed	Homo sapiens	BA10	NeuN+	Zymo EZ-96 direct	snmC-seq + SAP	Illumina HiSeq 4000	5423468	3347614	61.7%	70.3%	53.2%	2380172	71.1%	1928258	81.0%	0.00661	0.74892	0.02477	0.74725	0.01828	6.31
Pool_1732_AD010_indexed	Homo sapiens	BA10	NeuN+	Zymo EZ-96 direct	snmC-seq + SAP	Illumina HiSeq 4000	4034282	2520120	62.5%	73.5%	51.5%	1724093	68.4%	1400285	81.2%	0.01355	0.78558	0.06347	0.78263	0.05061	4.61
Pool_1747_AD006_indexed	Homo sapiens	BA10	NeuN+	Zymo EZ-96 direct	snmC-seq + SAP	Illumina HiSeq 4000	3901478	2055924	52.7%	60.0%	45.4%	1567188	76.2%	1261458	80.5%	0.01637	0.79266	0.05645	0.78921	0.04075	4.13
Pool_1750_AD006_indexed	Homo sapiens	BA10	NeuN+	Zymo EZ-96 direct	snmC-seq + SAP	Illumina HiSeq 4000	4850370	3064384	63.2%	72.7%	53.6%	2355150	76.9%	1903913	80.8%	0.01784	0.76767	0.04892	0.76345	0.03164	6.29
Pool_1751_AD006_indexed	Homo sapiens	BA10	NeuN+	Zymo EZ-96 direct	snmC-seq + SAP	Illumina HiSeq 4000	9738938	6331561	65.0%	74.5%	55.5%	3954400	62.5%	3222990	81.5%	0.01158	0.82473	0.04398	0.82268	0.03278	9.51
Pool_1758_AD010_indexed	Homo sapiens	BA10	NeuN+	Zymo EZ-96 direct	snmC-seq + SAP	Illumina HiSeq 4000	6244840	3880882	62.1%	72.4%	51.9%	2520234	64.9%	2048955	81.3%	0.00535	0.73736	0.00886	0.73595	0.00353	6.4
Pool_1769_AD006_indexed	Homo sapiens	BA10	NeuN+	Zymo EZ-96 direct	snmC-seq + SAP	Illumina HiSeq 4000	7195966	4621405	64.2%	70.8%	57.7%	3043671	65.9%	2524257	82.9%	0.00851	0.76633	0.02197	0.76432	0.01358	7.98
Pool_1775_AD010_indexed	Homo sapiens	BA10	NeuN+	Zymo EZ-96 direct	snmC-seq + SAP	Illumina HiSeq 4000	6479958	4366150	67.4%	76.2%	58.5%	2862208	65.6%	2321124	81.1%	0.01137	0.79872	0.054	0.79641	0.04312	7.51
Pool_1787_AD006_indexed	Homo sapiens	BA10	NeuN+	Zymo EZ-96 direct	snmC-seq + SAP	Illumina HiSeq 4000	10421900	7210031	69.2%	76.8%	61.6%	4244437	58.9%	3483859	82.1%	0.00384	0.75915	0.00685	0.75822	0.00302	11.46
Pool_1788_AD010_indexed	Homo sapiens	BA10	NeuN+	Zymo EZ-96 direct	snmC-seq + SAP	Illumina HiSeq 4000	7627894	5318148	69.7%	77.8%	61.6%	2985413	56.1%	2453968	82.2%	0.00406	0.7041	0.0063	0.70289	0.00225	8.16
Pool_1795_AD006_indexed	Homo sapiens	BA10	NeuN+	Zymo EZ-96 direct	snmC-seq + SAP	Illumina HiSeq 4000	6774890	4667455	68.9%	76.6%	61.1%	2810134	60.2%	2306094	82.1%	0.0074	0.76565	0.03343	0.7639	0.02622	7.86
Pool_1818_AD002_indexed	Homo sapiens	BA10	NeuN+	Zymo EZ-96 direct	snmC-seq + SAP	Illumina HiSeq 4000	6535912	2034995	31.1%	34.7%	27.6%	1407758	69.2%	1147622	81.5%	0.00636	0.76022	0.01841	0.75869	0.01213	4.1
Pool_1819_AD006_indexed	Homo sapiens	BA10	NeuN+	Zymo EZ-96 direct	snmC-seq + SAP	Illumina HiSeq 4000	2640564	1796216	68.0%	75.8%	60.3%	1309182	72.9%	1061298	81.1%	0.0077	0.80619	0.03464	0.80469	0.02715	3.87
Pool_1822_AD010_indexed	Homo sapiens	BA10	NeuN+	Zymo EZ-96 direct	snmC-seq + SAP	Illumina HiSeq 4000	4030150	2744194	68.1%	75.6%	60.6%	1837180	66.9%	1502084	81.8%	0.00776	0.80339	0.03372	0.80185	0.02616	5.33
Pool_1842_AD006_indexed	Homo sapiens	BA10	NeuN+	Zymo EZ-96 direct	snmC-seq + SAP	Illumina HiSeq 4000	8629638	5800083	67.2%	76.1%	58.4%	3318085	57.2%	2654476	80.0%	0.01235	0.8168	0.06121	0.81451	0.04947	8.36
Pool_1856_AD006_indexed	Homo sapiens	BA10	NeuN+	Zymo EZ-96 direct	snmC-seq + SAP	Illumina HiSeq 4000	13867330	9367485	67.6%	74.8%	60.3%	4463713	47.7%	3607138	80.8%	0.00468	0.759	0.0074	0.75787	0.00273	11.19
Pool_1859_AD006_indexed	Homo sapiens	BA10	NeuN+	Zymo EZ-96 direct	snmC-seq + SAP	Illumina HiSeq 4000	8148338	5627615	69.1%	76.6%	61.5%	3002908	53.4%	2464581	82.1%	0.00417	0.72648	0.00657	0.72533	0.00241	7.99
Pool_1872_AD002_indexed	Homo sapiens	BA10	NeuN+	Zymo EZ-96 direct	snmC-seq + SAP	Illumina HiSeq 4000	14745958	10344147	70.1%	79.2%	61.1%	5218386	50.4%	4251183	81.5%	0.00609	0.76445	0.01665	0.76301	0.01062	12.73
Pool_1872_AD010_indexed	Homo sapiens	BA10	NeuN+	Zymo EZ-96 direct	snmC-seq + SAP	Illumina HiSeq 4000	12327716	7619061	61.8%	69.2%	54.4%	3668562	48.1%	3002704	81.8%	0.00571	0.71433	0.00825	0.71269	0.00255	9.32
Pool_1985_AD006_indexed	Homo sapiens	BA10	NeuN+	Zymo EZ-96 direct	snmC-seq + SAP	Illumina HiSeq 4000	6182442	4073081	65.9%	73.4%	58.3%	2868469	70.4%	2372522	82.7%	0.01112	0.821	0.04981	0.81899	0.03913	8.26
Pool_1992_AD006_indexed	Homo sapiens	BA10	NeuN+	Zymo EZ-96 direct	snmC-seq + SAP	Illumina HiSeq 4000	7349248	4827412	65.7%	73.4%	58.0%	3061702	63.4%	2554529	83.4%	0.00789	0.76033	0.03464	0.75842	0.02696	8.65
Pool_2015_AD006_indexed	Homo sapiens	BA10	NeuN+	Zymo EZ-96 direct	snmC-seq + SAP	Illumina HiSeq 4000	9234172	6197916	67.1%	75.9%	58.3%	3677825	59.3%	3071084	83.5%	0.00549	0.72815	0.00764	0.72665	0.00216	10.05
Pool_2020_AD010_indexed	Homo sapiens	BA10	NeuN+	Zymo EZ-96 direct	snmC-seq + SAP	Illumina HiSeq 4000	16383962	11119953	67.9%	77.8%	58.0%	6265181	56.3%	5208446	83.1%	0.01503	0.72744	0.01927	0.72328	0.0043	15.93
Pool_2026_AD010_indexed	Homo sapiens	BA10	NeuN+	Zymo EZ-96 direct	snmC-seq + SAP	Illumina HiSeq 4000	12659460	8495495	67.1%	76.8%	57.4%	4349983	51.2%	3619609	83.2%	0.00964	0.76903	0.02054	0.76678	0.01101	11.2
Pool_2035_AD002_indexed	Homo sapiens	BA10	NeuN+	Zymo EZ-96 direct	snmC-seq + SAP	Illumina HiSeq 4000	9784064	6235090	63.7%	72.0%	55.4%	3547132	56.9%	2904550	81.9%	0.01022	0.79716	0.05161	0.79507	0.04182	8.98
Pool_2043_AD002_indexed	Homo sapiens	BA10	NeuN+	Zymo EZ-96 direct	snmC-seq + SAP	Illumina HiSeq 4000	9637976	6128252	63.6%	77.1%	50.1%	3317654	54.1%	2714460	81.8%	0.00801	0.80106	0.03702	0.79945	0.02924	8.58
Pool_2047_AD010_indexed	Homo sapiens	BA10	NeuN+	Zymo EZ-96 direct	snmC-seq + SAP	Illumina HiSeq 4000	13363514	8879417	66.4%	76.5%	56.4%	4483749	50.5%	3714252	82.8%	0.0096	0.77361	0.02187	0.77142	0.01239	11.38
Pool_2051_AD002_indexed	Homo sapiens	BA10	NeuN+	Zymo EZ-96 direct	snmC-seq + SAP	Illumina HiSeq 4000	11514236	7882377	68.5%	77.1%	59.8%	4226806	53.6%	3461560	81.9%	0.00855	0.78659	0.04276	0.78475	0.03451	10.93
Pool_2065_AD008_indexed	Homo sapiens	BA10	NeuN+	Zymo EZ-96 direct	snmC-seq + SAP	Illumina HiSeq 4000	24448374	15923465	65.1%	76.3%	54.0%	9871679	62.0%	7771966	78.7%	0.00563	0.77808	0.01822	0.77682	0.01266	21.57
Pool_2066_AD002_indexed	Homo sapiens	BA10	NeuN+	Zymo EZ-96 direct	snmC-seq + SAP	Illumina HiSeq 4000	5936786	3735578	62.9%	75.2%	50.6%	2429791	65.0%	1893359	77.9%	0.00628	0.77085	0.02336	0.7694	0.01719	5.94
Pool_2068_AD010_indexed	Homo sapiens	BA10	NeuN+	Zymo EZ-96 direct	snmC-seq + SAP	Illumina HiSeq 4000	8322790	4922449	59.1%	70.0%	48.3%	2919054	59.3%	2288241	78.4%	0.0195	0.8279	0.06654	0.82448	0.04798	6.99
Pool_2082_AD010_indexed	Homo sapiens	BA10	NeuN+	Zymo EZ-96 direct	snmC-seq + SAP	Illumina HiSeq 4000	9033406	6054132	67.0%	75.5%	58.6%	3122684	51.6%	2542058	81.4%	0.01589	0.82971	0.06022	0.82696	0.04505	7.98
Pool_2084_AD002_indexed	Homo sapiens	BA10	NeuN+	Zymo EZ-96 direct	snmC-seq + SAP	Illumina HiSeq 4000	5066372	3241253	64.0%	72.7%	55.3%	2041435	63.0%	1653169	81.0%	0.00909	0.75584	0.0372	0.7536	0.02837	5.47
Pool_2089_AD002_indexed	Homo sapiens	BA10	NeuN+	Zymo EZ-96 direct	snmC-seq + SAP	Illumina HiSeq 4000	6516974	4142289	63.6%	73.7%	53.4%	2443313	59.0%	1928819	78.9%	0.00977	0.7762	0.04691	0.77399	0.03751	6.1
Pool_2099_AD010_indexed	Homo sapiens	BA10	NeuN+	Zymo EZ-96 direct	snmC-seq + SAP	Illumina HiSeq 4000	10159174	6215596	61.2%	71.1%	51.3%	3347901	53.9%	2678160	80.0%	0.00626	0.76295	0.01691	0.76146	0.01072	8.31
Pool_2106_AD002_indexed	Homo sapiens	BA10	NeuN+	Zymo EZ-96 direct	snmC-seq + SAP	Illumina HiSeq 4000	9706306	6555983	67.5%	77.4%	57.7%	3255122	49.7%	2639992	81.1%	0.00723	0.795	0.033	0.79351	0.02596	7.97
Pool_2106_AD008_indexed	Homo sapiens	BA10	NeuN+	Zymo EZ-96 direct	snmC-seq + SAP	Illumina HiSeq 4000	13341190	8902236	66.7%	76.1%	57.4%	4208846	47.3%	3440019	81.7%	0.00979	0.76931	0.05099	0.76703	0.04161	10.31
Pool_2114_AD010_indexed	Homo sapiens	BA10	NeuN+	Zymo EZ-96 direct	snmC-seq + SAP	Illumina HiSeq 4000	19552614	11327860	57.9%	67.3%	48.6%	4673241	41.3%	3738698	80.0%	0.01157	0.78265	0.05388	0.78011	0.04281	10.51
Pool_2125_AD006_indexed	Homo sapiens	BA10	NeuN+	Zymo EZ-96 direct	snmC-seq + SAP	Illumina HiSeq 4000	15830968	10264321	64.8%	74.4%	55.2%	4594053	44.8%	3733936	81.3%	0.00445	0.69637	0.00672	0.69501	0.00228	10.48
Pool_2126_AD002_indexed	Homo sapiens	BA10	NeuN+	Zymo EZ-96 direct	snmC-seq + SAP	Illumina HiSeq 4000	7071962	4655994	65.8%	75.5%	56.2%	2424319	52.1%	1964475	81.0%	0.0069	0.76828	0.01939	0.76667	0.01258	6.05
Pool_2126_AD006_indexed	Homo sapiens	BA10	NeuN+	Zymo EZ-96 direct	snmC-seq + SAP	Illumina HiSeq 4000	10687000	7053833	66.0%	74.4%	57.6%	3503145	49.7%	2859743	81.6%	0.00959	0.81454	0.04705	0.81274	0.03782	8.76
Pool_2183_AD002_indexed	Homo sapiens	BA10	NeuN+	Zymo EZ-96 direct	snmC-seq + SAP	Illumina HiSeq 4000	1830614	1193416	65.2%	77.2%	53.2%	902588	75.6%	713670	79.1%	0.00479	0.73745	0.0093	0.73619	0.00453	2.41
Pool_2186_AD002_indexed	Homo sapiens	BA10	NeuN+	Zymo EZ-96 direct	snmC-seq + SAP	Illumina HiSeq 4000	3447788	2195297	63.7%	75.0%	52.3%	1693286	77.1%	1320484	78.0%	0.0084	0.7719	0.02868	0.76997	0.02045	4.4
Pool_2186_AD010_indexed	Homo sapiens	BA10	NeuN+	Zymo EZ-96 direct	snmC-seq + SAP	Illumina HiSeq 4000	3249604	1976276	60.8%	71.2%	50.5%	1494905	75.6%	1174652	78.6%	0.00741	0.73913	0.03	0.73718	0.02276	3.94
Pool_2196_AD008_indexed	Homo sapiens	BA10	NeuN+	Zymo EZ-96 direct	snmC-seq + SAP	Illumina HiSeq 4000	3054284	1900346	62.2%	73.9%	50.5%	1446555	76.1%	1151076	79.6%	0.00887	0.78797	0.03985	0.78607	0.03126	3.93
Pool_2199_AD010_indexed	Homo sapiens	BA10	NeuN+	Zymo EZ-96 direct	snmC-seq + SAP	Illumina HiSeq 4000	3965944	2543164	64.1%	74.9%	53.4%	1778904	69.9%	1423440	80.0%	0.00802	0.75056	0.04045	0.74854	0.03269	4.72
Pool_2211_AD002_indexed	Homo sapiens	BA10	NeuN+	Zymo EZ-96 direct	snmC-seq + SAP	Illumina HiSeq 4000	4938510	3235311	65.5%	77.9%	53.1%	2136158	66.0%	1697364	79.5%	0.00853	0.76516	0.04286	0.76314	0.03463	5.3
Pool_2214_AD002_indexed	Homo sapiens	BA10	NeuN+	Zymo EZ-96 direct	snmC-seq + SAP	Illumina HiSeq 4000	8393798	5572987	66.4%	78.6%	54.2%	3247438	58.3%	2595038	79.9%	0.00859	0.77167	0.04547	0.76969	0.0372	7.65
Pool_2219_AD006_indexed	Homo sapiens	BA10	NeuN+	Zymo EZ-96 direct	snmC-seq + SAP	Illumina HiSeq 4000	6552356	4345686	66.3%	76.7%	56.0%	2905208	66.9%	2339752	80.5%	0.0071	0.75301	0.03369	0.75124	0.02678	7.55
Pool_2220_AD002_indexed	Homo sapiens	BA10	NeuN+	Zymo EZ-96 direct	snmC-seq + SAP	Illumina HiSeq 4000	7015788	4512992	64.3%	76.4%	52.3%	2836561	62.9%	2253382	79.4%	0.00875	0.76039	0.04251	0.75827	0.03406	6.86
Pool_2224_AD006_indexed	Homo sapiens	BA10	NeuN+	Zymo EZ-96 direct	snmC-seq + SAP	Illumina HiSeq 4000	7286492	4840570	66.4%	76.7%	56.1%	3022216	62.4%	2429444	80.4%	0.00622	0.74426	0.02944	0.74266	0.02337	7.65
Pool_2228_AD010_indexed	Homo sapiens	BA10	NeuN+	Zymo EZ-96 direct	snmC-seq + SAP	Illumina HiSeq 4000	3519556	2126401	60.4%	70.2%	50.6%	1501527	70.6%	1158774	77.2%	0.00764	0.76781	0.03754	0.76602	0.03013	3.58
Pool_2231_AD010_indexed	Homo sapiens	BA10	NeuN+	Zymo EZ-96 direct	snmC-seq + SAP	Illumina HiSeq 4000	5288586	3314550	62.7%	74.3%	51.1%	2322292	70.1%	1789524	77.1%	0.00815	0.7684	0.04167	0.7665	0.0338	5.5
Pool_2240_AD002_indexed	Homo sapiens	BA10	NeuN+	Zymo EZ-96 direct	snmC-seq + SAP	Illumina HiSeq 4000	5344556	3160594	59.1%	70.0%	48.3%	2177200	68.9%	1717394	78.9%	0.02244	0.77339	0.05907	0.76819	0.03747	5.51
Pool_2241_AD002_indexed	Homo sapiens	BA10	NeuN+	Zymo EZ-96 direct	snmC-seq + SAP	Illumina HiSeq 4000	3792580	2501219	66.0%	76.8%	55.1%	1756366	70.2%	1392521	79.3%	0.00946	0.75659	0.04331	0.75427	0.03417	4.52
Pool_2244_AD002_indexed	Homo sapiens	BA10	NeuN+	Zymo EZ-96 direct	snmC-seq + SAP	Illumina HiSeq 4000	5950060	3912448	65.8%	78.0%	53.5%	2549638	65.2%	2017342	79.1%	0.00533	0.76008	0.01631	0.75879	0.01104	6.21
Pool_2245_AD010_indexed	Homo sapiens	BA10	NeuN+	Zymo EZ-96 direct	snmC-seq + SAP	Illumina HiSeq 4000	6048670	3918395	64.8%	75.1%	54.5%	2423254	61.8%	1930766	79.7%	0.01037	0.79608	0.05323	0.79394	0.04331	6.08
Pool_2368_AD008_indexed	Homo sapiens	BA10	NeuN+	Zymo EZ-96 direct	snmC-seq + SAP	Illumina HiSeq 4000	8203358	5822825	71.0%	79.7%	62.3%	2847158	48.9%	2427385	85.3%	0.01763	0.79656	0.08271	0.79291	0.06625	7.98
Pool_2371_AD002_indexed	Homo sapiens	BA10	NeuN+	Zymo EZ-96 direct	snmC-seq + SAP	Illumina HiSeq 4000	6342608	4529062	71.4%	80.2%	62.6%	2470476	54.5%	2101289	85.1%	0.01214	0.79695	0.06	0.79445	0.04845	7.07
Pool_2372_AD008_indexed	Homo sapiens	BA10	NeuN+	Zymo EZ-96 direct	snmC-seq + SAP	Illumina HiSeq 4000	7867900	5574691	70.9%	79.9%	61.8%	3004561	53.9%	2560599	85.2%	0.00713	0.80619	0.02739	0.8048	0.02041	8.46
Pool_2421_AD006_indexed	Homo sapiens	BA10	NeuN+	Zymo EZ-96 direct	snmC-seq + SAP	Illumina HiSeq 4000	11669878	8263062	70.8%	79.6%	62.0%	3519753	42.6%	3000910	85.3%	0.02108	0.82021	0.0967	0.81634	0.07725	8.93
Pool_2433_AD002_indexed	Homo sapiens	BA10	NeuN+	Zymo EZ-96 direct	snmC-seq + SAP	Illumina HiSeq 4000	7601306	5422629	71.3%	81.0%	61.7%	2482836	45.8%	2106894	84.9%	0.00496	0.77832	0.01468	0.77721	0.00977	6.62
Pool_2435_AD002_indexed	Homo sapiens	BA10	NeuN+	Zymo EZ-96 direct	snmC-seq + SAP	Illumina HiSeq 4000	7715878	5542819	71.8%	81.0%	62.7%	2702200	48.8%	2293950	84.9%	0.01006	0.81582	0.04151	0.81395	0.03177	7.44
Pool_2440_AD002_indexed	Homo sapiens	BA10	NeuN+	Zymo EZ-96 direct	snmC-seq + SAP	Illumina HiSeq 4000	9439296	6734816	71.3%	80.4%	62.3%	3220436	47.8%	2733163	84.9%	0.01002	0.80276	0.04072	0.80076	0.03101	8.71
Pool_2441_AD002_indexed	Homo sapiens	BA10	NeuN+	Zymo EZ-96 direct	snmC-seq + SAP	Illumina HiSeq 4000	12363650	8815790	71.3%	80.4%	62.2%	3286844	37.3%	2805728	85.4%	0.03832	0.81304	0.09108	0.80559	0.05486	8.23
Pool_2446_AD008_indexed	Homo sapiens	BA10	NeuN+	Zymo EZ-96 direct	snmC-seq + SAP	Illumina HiSeq 4000	8263426	5776871	69.9%	79.0%	60.8%	2418871	41.9%	2071481	85.6%	0.00523	0.69644	0.00761	0.69484	0.00239	6.28
Pool_2447_AD006_indexed	Homo sapiens	BA10	NeuN+	Zymo EZ-96 direct	snmC-seq + SAP	Illumina HiSeq 4000	11383066	7877188	69.2%	78.3%	60.1%	3605580	45.8%	3081609	85.5%	0.00831	0.81519	0.03292	0.81364	0.02482	9.6
171130_MB_EB_Hs_25yr_BA10_A6_AD012_indexed	Homo sapiens	BA10	NeuN+	Zymo-Spin 384 Well Plate	snmC-seq2	Illumina HiSeq 4000	5099872	3447162	67.6%	75.8%	59.3%	2806279	81.4%	2194578	78.2%	0.01774	0.83101	0.05402	0.82796	0.03694	7.76
171130_MB_EB_Hs_25yr_BA10_B1_AD001_indexed	Homo sapiens	BA10	NeuN+	Zymo-Spin 384 Well Plate	snmC-seq2	Illumina HiSeq 4000	5618476	3741894	66.6%	74.6%	58.6%	3027030	80.9%	2418488	79.9%	0.0119	0.85356	0.05311	0.8518	0.04171	8.49
171130_MB_EB_Hs_25yr_BA10_B1_AD010_indexed	Homo sapiens	BA10	NeuN+	Zymo-Spin 384 Well Plate	snmC-seq2	Illumina HiSeq 4000	4714498	3139161	66.6%	74.6%	58.6%	2542687	81.0%	1991122	78.3%	0.0396	0.86468	0.09083	0.8591	0.05334	7.03
171130_MB_EB_Hs_25yr_BA10_B3_AD010_indexed	Homo sapiens	BA10	NeuN+	Zymo-Spin 384 Well Plate	snmC-seq2	Illumina HiSeq 4000	4399844	2941793	66.9%	74.8%	58.9%	2401437	81.6%	1867246	77.8%	0.02141	0.80959	0.05787	0.80542	0.03726	6.66
171130_MB_EB_Hs_25yr_BA10_B7_AD001_indexed	Homo sapiens	BA10	NeuN+	Zymo-Spin 384 Well Plate	snmC-seq2	Illumina HiSeq 4000	5274098	3516079	66.7%	74.6%	58.8%	2737625	77.9%	2202953	80.5%	0.00729	0.83681	0.02394	0.83561	0.01677	7.7
171130_MB_EB_Hs_25yr_BA10_B7_AD002_indexed	Homo sapiens	BA10	NeuN+	Zymo-Spin 384 Well Plate	snmC-seq2	Illumina HiSeq 4000	4508470	2953522	65.5%	74.2%	56.8%	2324531	78.7%	1837913	79.1%	0.00597	0.82327	0.01	0.82221	0.00405	6.44
171130_MB_EB_Hs_25yr_BA10_B9_AD010_indexed	Homo sapiens	BA10	NeuN+	Zymo-Spin 384 Well Plate	snmC-seq2	Illumina HiSeq 4000	7787178	5440138	69.9%	79.3%	60.4%	4368983	80.3%	3435975	78.6%	0.38368	0.88204	0.46138	0.80861	0.12607	11.7
171130_MB_EB_Hs_25yr_BA10_C1_AD012_indexed	Homo sapiens	BA10	NeuN+	Zymo-Spin 384 Well Plate	snmC-seq2	Illumina HiSeq 4000	4215542	2825101	67.0%	74.6%	59.5%	2250407	79.7%	1761465	78.3%	0.01298	0.86034	0.0555	0.8585	0.04308	6.26
171130_MB_EB_Hs_25yr_BA10_D5_AD012_indexed	Homo sapiens	BA10	NeuN+	Zymo-Spin 384 Well Plate	snmC-seq2	Illumina HiSeq 4000	6116722	4115513	67.3%	74.7%	59.9%	3163742	76.9%	2444292	77.3%	0.01515	0.86584	0.06849	0.86378	0.05416	8.55
171130_MB_EB_Hs_25yr_BA10_E1_AD010_indexed	Homo sapiens	BA10	NeuN+	Zymo-Spin 384 Well Plate	snmC-seq2	Illumina HiSeq 4000	5405856	3674379	68.0%	75.4%	60.5%	2870313	78.1%	2263541	78.9%	0.02802	0.86166	0.07923	0.85767	0.05269	7.96
171130_MB_EB_Hs_25yr_BA10_E5_AD002_indexed	Homo sapiens	BA10	NeuN+	Zymo-Spin 384 Well Plate	snmC-seq2	Illumina HiSeq 4000	6611644	4411290	66.7%	75.7%	57.7%	3338597	75.7%	2657522	79.6%	0.01314	0.85091	0.06105	0.84892	0.04855	9.17
171130_MB_EB_Hs_25yr_BA10_F11_AD010_indexed	Homo sapiens	BA10	NeuN+	Zymo-Spin 384 Well Plate	snmC-seq2	Illumina HiSeq 4000	2440122	1598815	65.5%	73.2%	57.9%	1293354	80.9%	982745	76.0%	0.03103	0.81042	0.05975	0.80435	0.02964	3.51
171130_MB_EB_Hs_25yr_BA10_F5_AD001_indexed	Homo sapiens	BA10	NeuN+	Zymo-Spin 384 Well Plate	snmC-seq2	Illumina HiSeq 4000	5445456	3581677	65.8%	73.9%	57.7%	2797432	78.1%	2238063	80.0%	0.00969	0.81075	0.03993	0.8089	0.03054	7.78
171130_MB_EB_Hs_25yr_BA10_F6_AD002_indexed	Homo sapiens	BA10	NeuN+	Zymo-Spin 384 Well Plate	snmC-seq2	Illumina HiSeq 4000	5485112	3647374	66.5%	75.4%	57.6%	2792785	76.6%	2223688	79.6%	0.0126	0.86135	0.05553	0.85958	0.04348	7.74
171130_MB_EB_Hs_25yr_BA10_F7_AD001_indexed	Homo sapiens	BA10	NeuN+	Zymo-Spin 384 Well Plate	snmC-seq2	Illumina HiSeq 4000	5612630	3724044	66.4%	73.8%	58.9%	2798119	75.1%	2264182	80.9%	0.02184	0.8212	0.05543	0.81721	0.03434	7.84
171130_MB_EB_Hs_25yr_BA10_G10_AD001_indexed	Homo sapiens	BA10	NeuN+	Zymo-Spin 384 Well Plate	snmC-seq2	Illumina HiSeq 4000	4816972	3232421	67.1%	74.7%	59.5%	2637308	81.6%	2107987	79.9%	0.01456	0.85975	0.06528	0.85768	0.05147	7.51
171130_MB_EB_Hs_25yr_BA10_G2_AD012_indexed	Homo sapiens	BA10	NeuN+	Zymo-Spin 384 Well Plate	snmC-seq2	Illumina HiSeq 4000	3610266	2346221	65.0%	72.9%	57.1%	1971316	84.0%	1504084	76.3%	0.01	0.80852	0.03534	0.80659	0.0256	5.43
171130_MB_EB_Hs_25yr_BA10_G5_AD012_indexed	Homo sapiens	BA10	NeuN+	Zymo-Spin 384 Well Plate	snmC-seq2	Illumina HiSeq 4000	5655416	3813135	67.4%	74.8%	60.1%	2878098	75.5%	2221977	77.2%	0.0099	0.80329	0.04059	0.80132	0.031	7.71
171130_MB_EB_Hs_25yr_BA10_G8_AD012_indexed	Homo sapiens	BA10	NeuN+	Zymo-Spin 384 Well Plate	snmC-seq2	Illumina HiSeq 4000	5082560	3371865	66.3%	74.1%	58.6%	2709813	80.4%	2067239	76.3%	0.01041	0.81061	0.04073	0.80862	0.03064	7.31
171130_MB_EB_Hs_25yr_BA10_H9_AD001_indexed	Homo sapiens	BA10	NeuN+	Zymo-Spin 384 Well Plate	snmC-seq2	Illumina HiSeq 4000	2997794	1942037	64.8%	73.2%	56.4%	1618279	83.3%	1288554	79.6%	0.01418	0.8157	0.02054	0.81305	0.00645	4.6
171130_MB_EB_Hs_25yr_BA10_B12_AD002_indexed	Homo sapiens	BA10	NeuN+	Zymo-Spin 384 Well Plate	snmC-seq2	Illumina HiSeq 4000	4217488	2727796	64.7%	74.4%	54.9%	2339756	85.8%	1828894	78.2%	0.00903	0.80319	0.03536	0.8014	0.02657	6.47
171130_MB_EB_Hs_25yr_BA10_C12_AD002_indexed	Homo sapiens	BA10	NeuN+	Zymo-Spin 384 Well Plate	snmC-seq2	Illumina HiSeq 4000	4815106	3223206	66.9%	76.0%	57.9%	2516180	78.1%	2009329	79.9%	0.01336	0.85868	0.05877	0.85677	0.04602	7.04
171130_MB_EB_Hs_25yr_BA10_C7_AD010_indexed	Homo sapiens	BA10	NeuN+	Zymo-Spin 384 Well Plate	snmC-seq2	Illumina HiSeq 4000	6024380	4003543	66.5%	74.4%	58.5%	3173361	79.3%	2440296	76.9%	0.01709	0.83728	0.03382	0.83445	0.01702	8.52
171130_MB_EB_Hs_25yr_BA10_D12_AD002_indexed	Homo sapiens	BA10	NeuN+	Zymo-Spin 384 Well Plate	snmC-seq2	Illumina HiSeq 4000	4474586	2938212	65.7%	75.1%	56.3%	2386888	81.2%	1895269	79.4%	0.01717	0.86425	0.07892	0.86188	0.06283	6.75
171130_MB_EB_Hs_25yr_BA10_D1_AD010_indexed	Homo sapiens	BA10	NeuN+	Zymo-Spin 384 Well Plate	snmC-seq2	Illumina HiSeq 4000	4595044	3086119	67.2%	74.4%	59.9%	2442399	79.1%	1919840	78.6%	0.0271	0.81078	0.06524	0.80551	0.0392	6.79
171130_MB_EB_Hs_25yr_BA10_D3_AD012_indexed	Homo sapiens	BA10	NeuN+	Zymo-Spin 384 Well Plate	snmC-seq2	Illumina HiSeq 4000	5063256	3385053	66.9%	74.4%	59.3%	2731784	80.7%	2088683	76.5%	0.01215	0.85662	0.05463	0.85486	0.043	7.42
171130_MB_EB_Hs_25yr_BA10_D6_AD012_indexed	Homo sapiens	BA10	NeuN+	Zymo-Spin 384 Well Plate	snmC-seq2	Illumina HiSeq 4000	5184998	3514958	67.8%	75.6%	59.9%	2811793	80.0%	2165601	77.0%	0.00941	0.81071	0.03847	0.80891	0.02934	7.66
171130_MB_EB_Hs_25yr_BA10_D8_AD002_indexed	Homo sapiens	BA10	NeuN+	Zymo-Spin 384 Well Plate	snmC-seq2	Illumina HiSeq 4000	4162172	2712229	65.2%	75.1%	55.3%	2208223	81.4%	1724333	78.1%	0.01311	0.85766	0.06002	0.85577	0.04753	6.07
171130_MB_EB_Hs_25yr_BA10_E2_AD002_indexed	Homo sapiens	BA10	NeuN+	Zymo-Spin 384 Well Plate	snmC-seq2	Illumina HiSeq 4000	3393674	2265376	66.8%	75.6%	57.9%	1929015	85.2%	1538308	79.7%	0.00628	0.8161	0.00899	0.81494	0.00273	5.55
171130_MB_EB_Hs_25yr_BA10_E4_AD002_indexed	Homo sapiens	BA10	NeuN+	Zymo-Spin 384 Well Plate	snmC-seq2	Illumina HiSeq 4000	5098852	3296399	64.6%	74.1%	55.2%	2699491	81.9%	2084431	77.2%	0.01875	0.86224	0.08505	0.85961	0.06757	7.26
171130_MB_EB_Hs_25yr_BA10_F1_AD001_indexed	Homo sapiens	BA10	NeuN+	Zymo-Spin 384 Well Plate	snmC-seq2	Illumina HiSeq 4000	5602618	3720481	66.4%	74.4%	58.4%	2984690	80.2%	2388743	80.0%	0.01106	0.85399	0.04396	0.85236	0.03327	8.37
171130_MB_EB_Hs_25yr_BA10_F4_AD001_indexed	Homo sapiens	BA10	NeuN+	Zymo-Spin 384 Well Plate	snmC-seq2	Illumina HiSeq 4000	1619198	1067202	65.9%	73.5%	58.3%	843842	79.1%	675373	80.0%	0.00979	0.84792	0.03963	0.84642	0.03014	2.42
171130_MB_EB_Hs_25yr_BA10_G10_AD010_indexed	Homo sapiens	BA10	NeuN+	Zymo-Spin 384 Well Plate	snmC-seq2	Illumina HiSeq 4000	5305798	3563719	67.2%	74.3%	60.0%	2912753	81.7%	2235837	76.8%	0.02013	0.85777	0.0556	0.85485	0.0362	7.95
171130_MB_EB_Hs_25yr_BA10_G5_AD001_indexed	Homo sapiens	BA10	NeuN+	Zymo-Spin 384 Well Plate	snmC-seq2	Illumina HiSeq 4000	7255772	4879350	67.2%	74.7%	59.8%	3682039	75.5%	2944440	80.0%	0.01544	0.8586	0.05361	0.85638	0.03877	10.09
171130_MB_EB_Hs_25yr_BA10_G5_AD010_indexed	Homo sapiens	BA10	NeuN+	Zymo-Spin 384 Well Plate	snmC-seq2	Illumina HiSeq 4000	6496458	4393961	67.6%	74.8%	60.4%	3320268	75.6%	2575955	77.6%	0.02068	0.81189	0.05358	0.80792	0.03359	8.89
171130_MB_EB_Hs_25yr_BA10_G7_AD002_indexed	Homo sapiens	BA10	NeuN+	Zymo-Spin 384 Well Plate	snmC-seq2	Illumina HiSeq 4000	3713774	2431618	65.5%	74.9%	56.0%	2057604	84.6%	1617767	78.6%	0.00677	0.82352	0.01774	0.82232	0.01104	5.78
171130_MB_EB_Hs_25yr_BA10_G8_AD002_indexed	Homo sapiens	BA10	NeuN+	Zymo-Spin 384 Well Plate	snmC-seq2	Illumina HiSeq 4000	4373450	2853405	65.2%	74.5%	56.0%	2318883	81.3%	1824952	78.7%	0.01165	0.85834	0.0513	0.85667	0.04012	6.46
171130_MB_EB_Hs_25yr_BA10_H3_AD012_indexed	Homo sapiens	BA10	NeuN+	Zymo-Spin 384 Well Plate	snmC-seq2	Illumina HiSeq 4000	2617434	1710408	65.3%	73.2%	57.5%	1447024	84.6%	1090230	75.3%	0.01185	0.86129	0.05136	0.85963	0.03998	3.95
171130_MB_EB_Hs_25yr_BA10_H8_AD012_indexed	Homo sapiens	BA10	NeuN+	Zymo-Spin 384 Well Plate	snmC-seq2	Illumina HiSeq 4000	5372804	3594341	66.9%	74.6%	59.2%	2868537	79.8%	2202597	76.8%	0.02021	0.82203	0.03674	0.81836	0.01687	7.72
171130_MB_EB_Hs_25yr_BA10_A3_AD012_indexed	Homo sapiens	BA10	NeuN+	Zymo-Spin 384 Well Plate	snmC-seq2	Illumina HiSeq 4000	5308608	3402301	64.1%	71.5%	56.7%	2792183	82.1%	2174260	77.9%	0.00625	0.81826	0.00888	0.81712	0.00265	7.71
171130_MB_EB_Hs_25yr_BA10_A5_AD012_indexed	Homo sapiens	BA10	NeuN+	Zymo-Spin 384 Well Plate	snmC-seq2	Illumina HiSeq 4000	6837150	4619241	67.6%	75.7%	59.5%	3460965	74.9%	2724354	78.7%	0.01297	0.857	0.05892	0.85512	0.04655	9.38
171130_MB_EB_Hs_25yr_BA10_B1_AD002_indexed	Homo sapiens	BA10	NeuN+	Zymo-Spin 384 Well Plate	snmC-seq2	Illumina HiSeq 4000	5551304	3537607	63.7%	73.0%	54.5%	2909874	82.3%	2308072	79.3%	0.01698	0.84042	0.05029	0.83766	0.03389	8.03
171130_MB_EB_Hs_25yr_BA10_B2_AD002_indexed	Homo sapiens	BA10	NeuN+	Zymo-Spin 384 Well Plate	snmC-seq2	Illumina HiSeq 4000	3821618	2514937	65.8%	75.3%	56.3%	2103915	83.7%	1673170	79.5%	0.01026	0.81719	0.03848	0.81529	0.02851	6
171130_MB_EB_Hs_25yr_BA10_C8_AD012_indexed	Homo sapiens	BA10	NeuN+	Zymo-Spin 384 Well Plate	snmC-seq2	Illumina HiSeq 4000	5281716	3575102	67.7%	75.3%	60.1%	2820410	78.9%	2177957	77.2%	0.01108	0.81488	0.049	0.81281	0.03834	7.67
171130_MB_EB_Hs_25yr_BA10_D12_AD012_indexed	Homo sapiens	BA10	NeuN+	Zymo-Spin 384 Well Plate	snmC-seq2	Illumina HiSeq 4000	4181972	2774678	66.3%	74.2%	58.5%	2238532	80.7%	1720589	76.9%	0.04482	0.81017	0.08901	0.80126	0.04626	6.12
171130_MB_EB_Hs_25yr_BA10_D2_AD001_indexed	Homo sapiens	BA10	NeuN+	Zymo-Spin 384 Well Plate	snmC-seq2	Illumina HiSeq 4000	3501086	2266388	64.7%	73.0%	56.5%	1967319	86.8%	1557311	79.2%	0.00636	0.80376	0.00886	0.8025	0.00252	5.56
171130_MB_EB_Hs_25yr_BA10_E11_AD010_indexed	Homo sapiens	BA10	NeuN+	Zymo-Spin 384 Well Plate	snmC-seq2	Illumina HiSeq 4000	4920356	3236803	65.8%	73.6%	57.9%	2646600	81.8%	2020536	76.3%	0.02073	0.83577	0.05214	0.83229	0.03207	7.16
171130_MB_EB_Hs_25yr_BA10_E4_AD010_indexed	Homo sapiens	BA10	NeuN+	Zymo-Spin 384 Well Plate	snmC-seq2	Illumina HiSeq 4000	5739954	3787480	66.0%	74.0%	58.0%	3062653	80.9%	2339518	76.4%	0.02269	0.81094	0.0639	0.80655	0.04217	8.13
171130_MB_EB_Hs_25yr_BA10_E4_AD012_indexed	Homo sapiens	BA10	NeuN+	Zymo-Spin 384 Well Plate	snmC-seq2	Illumina HiSeq 4000	4737004	3130619	66.1%	74.2%	57.9%	2524921	80.7%	1923129	76.2%	0.01726	0.86575	0.07699	0.86339	0.06078	6.74
171130_MB_EB_Hs_25yr_BA10_E6_AD010_indexed	Homo sapiens	BA10	NeuN+	Zymo-Spin 384 Well Plate	snmC-seq2	Illumina HiSeq 4000	4521676	3036002	67.1%	74.6%	59.7%	2348368	77.4%	1827113	77.8%	0.01946	0.8141	0.02562	0.81041	0.00628	6.38
171130_MB_EB_Hs_25yr_BA10_E7_AD002_indexed	Homo sapiens	BA10	NeuN+	Zymo-Spin 384 Well Plate	snmC-seq2	Illumina HiSeq 4000	5261416	3494752	66.4%	75.3%	57.5%	2663411	76.2%	2124676	79.8%	0.00929	0.79716	0.03578	0.79526	0.02674	7.37
171130_MB_EB_Hs_25yr_BA10_F12_AD002_indexed	Homo sapiens	BA10	NeuN+	Zymo-Spin 384 Well Plate	snmC-seq2	Illumina HiSeq 4000	4875000	3221610	66.1%	74.9%	57.2%	2553060	79.2%	2028651	79.5%	0.00928	0.80875	0.03743	0.80696	0.02841	7.11
171130_MB_EB_Hs_25yr_BA10_F1_AD010_indexed	Homo sapiens	BA10	NeuN+	Zymo-Spin 384 Well Plate	snmC-seq2	Illumina HiSeq 4000	4551118	2999857	65.9%	73.6%	58.3%	2411465	80.4%	1873256	77.7%	0.02088	0.75287	0.0264	0.7476	0.00564	6.58
171130_MB_EB_Hs_25yr_BA10_F2_AD012_indexed	Homo sapiens	BA10	NeuN+	Zymo-Spin 384 Well Plate	snmC-seq2	Illumina HiSeq 4000	3760078	2527829	67.2%	74.9%	59.6%	2083326	82.4%	1594923	76.6%	0.01001	0.809	0.04258	0.80707	0.0329	5.75
171130_MB_EB_Hs_25yr_BA10_G1_AD002_indexed	Homo sapiens	BA10	NeuN+	Zymo-Spin 384 Well Plate	snmC-seq2	Illumina HiSeq 4000	2993798	1949905	65.1%	74.5%	55.7%	1665252	85.4%	1313755	78.9%	0.01361	0.86084	0.06051	0.85892	0.04755	4.75
171130_MB_EB_Hs_25yr_BA10_G3_AD010_indexed	Homo sapiens	BA10	NeuN+	Zymo-Spin 384 Well Plate	snmC-seq2	Illumina HiSeq 4000	4172542	2728760	65.4%	73.4%	57.4%	2279187	83.5%	1742444	76.5%	0.02108	0.84737	0.04637	0.84408	0.02583	6.21
171130_MB_EB_Hs_25yr_BA10_G3_AD012_indexed	Homo sapiens	BA10	NeuN+	Zymo-Spin 384 Well Plate	snmC-seq2	Illumina HiSeq 4000	4318718	2836242	65.7%	74.1%	57.2%	2363211	83.3%	1788265	75.7%	0.01986	0.87424	0.08955	0.87169	0.0711	6.42
171130_MB_EB_Hs_25yr_BA10_H2_AD010_indexed	Homo sapiens	BA10	NeuN+	Zymo-Spin 384 Well Plate	snmC-seq2	Illumina HiSeq 4000	2993540	1985680	66.3%	74.1%	58.6%	1693389	85.3%	1321101	78.0%	0.0512	0.80314	0.09282	0.79252	0.04387	4.8
171130_MB_EB_Hs_25yr_BA10_H7_AD010_indexed	Homo sapiens	BA10	NeuN+	Zymo-Spin 384 Well Plate	snmC-seq2	Illumina HiSeq 4000	5392810	3541973	65.7%	73.7%	57.6%	2825413	79.8%	2192097	77.6%	0.02675	0.86137	0.08467	0.85756	0.05951	7.72
171130_MB_EB_Hs_25yr_BA10_A12_AD010_indexed	Homo sapiens	BA10	NeuN+	Zymo-Spin 384 Well Plate	snmC-seq2	Illumina HiSeq 4000	5178590	3421195	66.1%	74.0%	58.1%	2747760	80.3%	2147707	78.2%	0.02515	0.85623	0.07426	0.85252	0.05038	7.59
171130_MB_EB_Hs_25yr_BA10_A1_AD001_indexed	Homo sapiens	BA10	NeuN+	Zymo-Spin 384 Well Plate	snmC-seq2	Illumina HiSeq 4000	4471834	2994417	67.0%	74.7%	59.2%	2467840	82.4%	1986008	80.5%	0.01234	0.85253	0.04924	0.85069	0.03736	7.06
171130_MB_EB_Hs_25yr_BA10_A5_AD001_indexed	Homo sapiens	BA10	NeuN+	Zymo-Spin 384 Well Plate	snmC-seq2	Illumina HiSeq 4000	6301552	4193498	66.5%	74.6%	58.5%	3154260	75.2%	2540650	80.5%	0.01267	0.85098	0.0558	0.84907	0.04368	8.75
171130_MB_EB_Hs_25yr_BA10_A7_AD001_indexed	Homo sapiens	BA10	NeuN+	Zymo-Spin 384 Well Plate	snmC-seq2	Illumina HiSeq 4000	6346926	4271187	67.3%	75.2%	59.3%	3336228	78.1%	2689231	80.6%	0.01108	0.85007	0.04416	0.84839	0.03345	9.36
171130_MB_EB_Hs_25yr_BA10_B1_AD012_indexed	Homo sapiens	BA10	NeuN+	Zymo-Spin 384 Well Plate	snmC-seq2	Illumina HiSeq 4000	5199730	3492069	67.2%	75.3%	59.0%	2818217	80.7%	2213943	78.6%	0.01335	0.86787	0.05787	0.86608	0.04512	7.8
171130_MB_EB_Hs_25yr_BA10_B9_AD001_indexed	Homo sapiens	BA10	NeuN+	Zymo-Spin 384 Well Plate	snmC-seq2	Illumina HiSeq 4000	4803736	3246058	67.6%	75.4%	59.8%	2553969	78.7%	2059638	80.6%	0.01088	0.79716	0.04816	0.79493	0.03769	7.25

171130_MB_EB_Hs_25yr_BA10_C4_AD002_indexed	Homo sapiens	BA10	NeuN+	Zymo-Spin 384 Well Plate	snmC-seq2	Illumina HiSeq 4000	5692524	3666614	64.4%	74.4%	54.4%	2912129	79.4%	2302679	79.1%	0.01388	0.83617	0.06321	0.83386	0.05002	7.98
171130_MB_EB_Hs_25yr_BA10_C7_AD002_indexed	Homo sapiens	BA10	NeuN+	Zymo-Spin 384 Well Plate	snmC-seq2	Illumina HiSeq 4000	4982350	3152871	63.3%	72.7%	53.9%	2518153	79.9%	2013680	80.0%	0.01168	0.80116	0.04631	0.79881	0.03504	7.08
171130_MB_EB_Hs_25yr_BA10_D12_AD001_indexed	Homo sapiens	BA10	NeuN+	Zymo-Spin 384 Well Plate	snmC-seq2	Illumina HiSeq 4000	5140650	3431970	66.8%	75.0%	58.5%	2765854	80.6%	2220124	80.3%	0.00864	0.80513	0.0353	0.80343	0.02689	7.83
171130_MB_EB_Hs_25yr_BA10_D4_AD001_indexed	Homo sapiens	BA10	NeuN+	Zymo-Spin 384 Well Plate	snmC-seq2	Illumina HiSeq 4000	5276964	3572497	67.7%	76.0%	59.4%	2803036	78.5%	2249412	80.2%	0.0108	0.8105	0.04704	0.80843	0.03664	7.89
171130_MB_EB_Hs_25yr_BA10_E11_AD012_indexed	Homo sapiens	BA10	NeuN+	Zymo-Spin 384 Well Plate	snmC-seq2	Illumina HiSeq 4000	4315592	2851577	66.1%	74.3%	57.9%	2326414	81.6%	1762787	75.8%	0.01174	0.85446	0.0513	0.85273	0.04003	6.24
171130_MB_EB_Hs_25yr_BA10_E3_AD002_indexed	Homo sapiens	BA10	NeuN+	Zymo-Spin 384 Well Plate	snmC-seq2	Illumina HiSeq 4000	2915546	1916315	65.7%	75.3%	56.2%	1645345	85.9%	1301018	79.1%	0.01017	0.81581	0.04226	0.81392	0.03242	4.73
171130_MB_EB_Hs_25yr_BA10_E3_AD010_indexed	Homo sapiens	BA10	NeuN+	Zymo-Spin 384 Well Plate	snmC-seq2	Illumina HiSeq 4000	3469710	2317309	66.8%	74.4%	59.2%	1974456	85.2%	1526448	77.3%	0.01861	0.82799	0.0302	0.82473	0.01181	5.52
171130_MB_EB_Hs_25yr_BA10_F3_AD010_indexed	Homo sapiens	BA10	NeuN+	Zymo-Spin 384 Well Plate	snmC-seq2	Illumina HiSeq 4000	2948954	1959211	66.4%	74.4%	58.5%	1634835	83.4%	1254312	76.7%	0.03134	0.81742	0.07925	0.81151	0.04946	4.53
171130_MB_EB_Hs_25yr_BA10_F9_AD001_indexed	Homo sapiens	BA10	NeuN+	Zymo-Spin 384 Well Plate	snmC-seq2	Illumina HiSeq 4000	5048850	3370587	66.8%	74.3%	59.2%	2680665	79.5%	2146214	80.1%	0.00642	0.81562	0.01104	0.81443	0.00465	7.54
171130_MB_EB_Hs_25yr_BA10_F9_AD002_indexed	Homo sapiens	BA10	NeuN+	Zymo-Spin 384 Well Plate	snmC-seq2	Illumina HiSeq 4000	4113478	2742520	66.7%	75.6%	57.8%	2194603	80.0%	1747590	79.6%	0.00855	0.84049	0.03194	0.83911	0.02359	6.19
171130_MB_EB_Hs_25yr_BA10_G4_AD012_indexed	Homo sapiens	BA10	NeuN+	Zymo-Spin 384 Well Plate	snmC-seq2	Illumina HiSeq 4000	5570132	3686583	66.2%	74.1%	58.2%	2944635	79.9%	2254147	76.6%	0.00694	0.79224	0.01503	0.79079	0.00815	7.91
171130_MB_EB_Hs_25yr_BA10_H6_AD002_indexed	Homo sapiens	BA10	NeuN+	Zymo-Spin 384 Well Plate	snmC-seq2	Illumina HiSeq 4000	2675634	1774329	66.3%	75.2%	57.5%	1371087	77.3%	1094195	79.8%	0.01469	0.84065	0.0599	0.83827	0.04588	3.87
171213_CEMBA_mm_P56_P63_3C_MOp_CEMBA171206_3C_1_CEMBA171206_3C_3_A1_AD001	Mus musculus	Primary motor cort	NeuN+	Zymo-Spin 384 Well Plate	snmC-seq3	Illumina Novaseq	5808566	3793962	65.3%	68.0%	62.7%	2593874	68.4%	2263072	87.2%	0.00685	0.7727	0.02376	0.77113	0.01703	9.14
171213_CEMBA_mm_P56_P63_3C_MOp_CEMBA171206_3C_1_CEMBA171206_3C_3_A1_AD002	Mus musculus	Primary motor cort	NeuN+	Zymo-Spin 384 Well Plate	snmC-seq4	Illumina Novaseq	6700710	4488754	67.0%	69.9%	64.1%	3061671	68.2%	2652529	86.6%	0.00834	0.79224	0.03282	0.79049	0.02469	10.65
171213_CEMBA_mm_P56_P63_3C_MOp_CEMBA171206_3C_1_CEMBA171206_3C_3_A1_AD004	Mus musculus	Primary motor cort	NeuN+	Zymo-Spin 384 Well Plate	snmC-seq5	Illumina Novaseq	6047684	4039906	66.8%	69.2%	64.4%	2772701	68.6%	2395144	86.4%	0.00678	0.75866	0.02207	0.75701	0.01539	9.64
171213_CEMBA_mm_P56_P63_3C_MOp_CEMBA171206_3C_1_CEMBA171206_3C_3_A1_AD006	Mus musculus	Primary motor cort	NeuN+	Zymo-Spin 384 Well Plate	snmC-seq6	Illumina Novaseq	7120648	4603951	64.7%	66.7%	62.6%	3161923	68.7%	2711859	85.8%	0.00865	0.77097	0.03338	0.76897	0.02495	10.85
171213_CEMBA_mm_P56_P63_3C_MOp_CEMBA171206_3C_1_CEMBA171206_3C_3_A1_AD007	Mus musculus	Primary motor cort	NeuN+	Zymo-Spin 384 Well Plate	snmC-seq7	Illumina Novaseq	7106416	4724488	66.5%	68.8%	64.2%	3225993	68.3%	2740046	84.9%	0.00758	0.75241	0.03076	0.75052	0.02336	10.96
171213_CEMBA_mm_P56_P63_3C_MOp_CEMBA171206_3C_1_CEMBA171206_3C_3_A1_AD008	Mus musculus	Primary motor cort	NeuN+	Zymo-Spin 384 Well Plate	snmC-seq8	Illumina Novaseq	5641778	3681269	65.3%	67.4%	63.1%	2527011	68.6%	2130692	84.3%	0.00763	0.77805	0.02874	0.77634	0.02127	8.6
171213_CEMBA_mm_P56_P63_3C_MOp_CEMBA171206_3C_1_CEMBA171206_3C_3_A1_AD010	Mus musculus	Primary motor cort	NeuN+	Zymo-Spin 384 Well Plate	snmC-seq9	Illumina Novaseq	4839718	3163573	65.4%	67.6%	63.1%	2168429	68.5%	1805855	83.3%	0.00824	0.78497	0.02886	0.78318	0.02079	7.31
171213_CEMBA_mm_P56_P63_3C_MOp_CEMBA171206_3C_1_CEMBA171206_3C_3_A1_AD012	Mus musculus	Primary motor cort	NeuN+	Zymo-Spin 384 Well Plate	snmC-seq10	Illumina Novaseq	6140928	4076326	66.4%	68.8%	64.0%	2797940	68.6%	2341360	83.7%	0.00736	0.74321	0.02572	0.74131	0.0185	9.41
171213_CEMBA_mm_P56_P63_3C_MOp_CEMBA171206_3C_1_CEMBA171206_3C_3_A10_AD001	Mus musculus	Primary motor cort	NeuN+	Zymo-Spin 384 Well Plate	snmC-seq11	Illumina Novaseq	2896392	1812485	62.6%	65.3%	59.9%	1475086	81.4%	1195574	81.1%	0.00738	0.75953	0.02543	0.75774	0.01818	4.92
171213_CEMBA_mm_P56_P63_3C_MOp_CEMBA171206_3C_1_CEMBA171206_3C_3_A10_AD002	Mus musculus	Primary motor cort	NeuN+	Zymo-Spin 384 Well Plate	snmC-seq12	Illumina Novaseq	3306366	2086717	63.1%	66.2%	60.0%	1685218	80.8%	1355517	80.4%	0.00938	0.77904	0.03741	0.77695	0.0283	5.58
171213_CEMBA_mm_P56_P63_3C_MOp_CEMBA171206_3C_1_CEMBA171206_3C_3_A10_AD004	Mus musculus	Primary motor cort	NeuN+	Zymo-Spin 384 Well Plate	snmC-seq13	Illumina Novaseq	3972894	2517605	63.4%	65.8%	60.9%	2032935	80.7%	1631274	80.2%	0.00746	0.75266	0.02216	0.7508	0.01481	6.65
171213_CEMBA_mm_P56_P63_3C_MOp_CEMBA171206_3C_1_CEMBA171206_3C_3_A10_AD007	Mus musculus	Primary motor cort	NeuN+	Zymo-Spin 384 Well Plate	snmC-seq14	Illumina Novaseq	3772120	2359076	62.5%	65.0%	60.0%	1903348	80.7%	1479211	77.7%	0.00701	0.75509	0.02432	0.75336	0.01743	6.02
171213_CEMBA_mm_P56_P63_3C_MOp_CEMBA171206_3C_1_CEMBA171206_3C_3_A10_AD008	Mus musculus	Primary motor cort	NeuN+	Zymo-Spin 384 Well Plate	snmC-seq15	Illumina Novaseq	3314196	2063268	62.3%	64.6%	59.9%	1671265	81.0%	1293662	77.4%	0.00683	0.76675	0.02134	0.76515	0.01461	5.28
171213_CEMBA_mm_P56_P63_3C_MOp_CEMBA171206_3C_1_CEMBA171206_3C_3_A10_AD010	Mus musculus	Primary motor cort	NeuN+	Zymo-Spin 384 Well Plate	snmC-seq16	Illumina Novaseq	3748522	2350911	62.7%	65.1%	60.3%	1903022	80.9%	1464874	77.0%	0.00787	0.75533	0.02884	0.75339	0.02114	5.96
171213_CEMBA_mm_P56_P63_3C_MOp_CEMBA171206_3C_1_CEMBA171206_3C_3_A10_AD012	Mus musculus	Primary motor cort	NeuN+	Zymo-Spin 384 Well Plate	snmC-seq17	Illumina Novaseq	3487424	2174584	62.4%	64.9%	59.8%	1760912	81.0%	1354225	76.9%	0.00704	0.75947	0.02107	0.75776	0.01413	5.53
171213_CEMBA_mm_P56_P63_3C_MOp_CEMBA171206_3C_1_CEMBA171206_3C_3_A11_AD001	Mus musculus	Primary motor cort	NeuN-	Zymo-Spin 384 Well Plate	snmC-seq18	Illumina Novaseq	6116788	4093733	66.9%	69.0%	64.8%	2791389	68.2%	2427787	87.0%	0.00611	0.76064	0.01985	0.75917	0.01382	9.74
171213_CEMBA_mm_P56_P63_3C_MOp_CEMBA171206_3C_1_CEMBA171206_3C_3_A11_AD002	Mus musculus	Primary motor cort	NeuN+	Zymo-Spin 384 Well Plate	snmC-seq19	Illumina Novaseq	5616334	3790448	67.5%	69.9%	65.0%	2598858	68.6%	2224459	85.6%	0.00739	0.75106	0.02558	0.74921	0.01833	8.97
171213_CEMBA_mm_P56_P63_3C_MOp_CEMBA171206_3C_1_CEMBA171206_3C_3_A11_AD004	Mus musculus	Primary motor cort	NeuN+	Zymo-Spin 384 Well Plate	snmC-seq20	Illumina Novaseq	6819872	4619547	67.7%	69.7%	65.8%	3164070	68.5%	2698943	85.3%	0.00701	0.75225	0.02035	0.7505	0.01343	10.76
171213_CEMBA_mm_P56_P63_3C_MOp_CEMBA171206_3C_1_CEMBA171206_3C_3_A11_AD006	Mus musculus	Primary motor cort	NeuN+	Zymo-Spin 384 Well Plate	snmC-seq21	Illumina Novaseq	7382298	4854305	65.8%	67.5%	64.0%	3337099	68.7%	2815807	84.4%	0.00915	0.8243	0.03678	0.82268	0.02789	11.21
171213_CEMBA_mm_P56_P63_3C_MOp_CEMBA171206_3C_1_CEMBA171206_3C_3_A11_AD007	Mus musculus	Primary motor cort	NeuN+	Zymo-Spin 384 Well Plate	snmC-seq22	Illumina Novaseq	14656874	9960544	68.0%	70.0%	65.9%	6786486	68.1%	5669927	83.5%	0.00591	0.73898	0.01666	0.73743	0.01081	21.07
171213_CEMBA_mm_P56_P63_3C_MOp_CEMBA171206_3C_1_CEMBA171206_3C_3_A11_AD008	Mus musculus	Primary motor cort	NeuN+	Zymo-Spin 384 Well Plate	snmC-seq23	Illumina Novaseq	7483380	4224014	56.4%	58.2%	54.7%	2882527	68.2%	2432247	84.4%	0.00905	0.81054	0.03743	0.80881	0.02864	9.74
171213_CEMBA_mm_P56_P63_3C_MOp_CEMBA171206_3C_1_CEMBA171206_3C_3_A11_AD010	Mus musculus	Primary motor cort	NeuN+	Zymo-Spin 384 Well Plate	snmC-seq24	Illumina Novaseq	7003244	4695395	67.0%	69.1%	65.0%	3196725	68.1%	2682795	83.9%	0.00876	0.81735	0.03446	0.81574	0.02593	10.69
171213_CEMBA_mm_P56_P63_3C_MOp_CEMBA171206_3C_1_CEMBA171206_3C_3_A11_AD012	Mus musculus	Primary motor cort	NeuN+	Zymo-Spin 384 Well Plate	snmC-seq25	Illumina Novaseq	4671922	3128423	67.0%	69.3%	64.6%	2122862	67.9%	1753755	82.6%	0.00862	0.80579	0.035	0.8041	0.02661	7.09
171213_CEMBA_mm_P56_P63_3C_MOp_CEMBA171206_3C_1_CEMBA171206_3C_3_A12_AD006	Mus musculus	Primary motor cort	NeuN+	Zymo-Spin 384 Well Plate	snmC-seq26	Illumina Novaseq	4203814	2372424	56.4%	57.7%	55.2%	1570487	66.2%	1329484	84.7%	0.0061	0.72391	0.00999	0.72222	0.00391	5.36
171213_CEMBA_mm_P56_P63_3C_MOp_CEMBA171207_3C_1_CEMBA171207_3C_2_A1_AD007	Mus musculus	Primary motor cort	NeuN+	Zymo-Spin 384 Well Plate	snmC-seq27	Illumina Novaseq	6572666	4412041	67.1%	69.1%	65.1%	3223253	73.1%	2794138	86.7%	0.00793	0.77695	0.032	0.77517	0.02426	11.24
171213_CEMBA_mm_P56_P63_3C_MOp_CEMBA171207_3C_1_CEMBA171207_3C_2_A1_AD010	Mus musculus	Primary motor cort	NeuN+	Zymo-Spin 384 Well Plate	snmC-seq28	Illumina Novaseq	4809826	3209992	66.7%	68.8%	64.6%	2358797	73.5%	2032235	86.2%	0.00742	0.756	0.02465	0.75418	0.01736	8.36
171213_CEMBA_mm_P56_P63_3C_MOp_CEMBA171207_3C_1_CEMBA171207_3C_2_A1_AD012	Mus musculus	Primary motor cort	NeuN+	Zymo-Spin 384 Well Plate	snmC-seq29	Illumina Novaseq	3846472	2325684	60.5%	62.5%	58.5%	1695643	72.9%	1455705	85.8%	0.00792	0.75499	0.02697	0.75303	0.0192	6.04
171213_CEMBA_mm_P56_P63_3C_MOp_CEMBA171207_3C_1_CEMBA171207_3C_2_A10_AD001	Mus musculus	Primary motor cort	NeuN+	Zymo-Spin 384 Well Plate	snmC-seq30	Illumina Novaseq	5033070	3370002	67.0%	69.9%	64.0%	2531011	75.1%	2196514	86.8%	0.005	0.75388	0.01876	0.75264	0.01383	8.98
171213_CEMBA_mm_P56_P63_3C_MOp_CEMBA171207_3C_1_CEMBA171207_3C_2_A10_AD002	Mus musculus	Primary motor cort	NeuN+	Zymo-Spin 384 Well Plate	snmC-seq31	Illumina Novaseq	4358850	2941775	67.5%	70.7%	64.3%	2206494	75.0%	1900791	86.1%	0.0072	0.75714	0.0258	0.75538	0.01873	7.84
171213_CEMBA_mm_P56_P63_3C_MOp_CEMBA171207_3C_1_CEMBA171207_3C_2_A10_AD004	Mus musculus	Primary motor cort	NeuN+	Zymo-Spin 384 Well Plate	snmC-seq32	Illumina Novaseq	5130966	3452562	67.3%	69.9%	64.7%	2597502	75.2%	2215720	85.3%	0.0068	0.7747	0.02385	0.77316	0.01717	9.05
171213_CEMBA_mm_P56_P63_3C_MOp_CEMBA171207_3C_1_CEMBA171207_3C_2_A10_AD006	Mus musculus	Primary motor cort	NeuN+	Zymo-Spin 384 Well Plate	snmC-seq33	Illumina Novaseq	5691338	3689304	64.8%	67.0%	62.6%	2783635	75.5%	2378063	85.4%	0.0076	0.75318	0.02711	0.75129	0.01966	9.67
171213_CEMBA_mm_P56_P63_3C_MOp_CEMBA171207_3C_1_CEMBA171207_3C_2_A10_AD007	Mus musculus	Primary motor cort	NeuN+	Zymo-Spin 384 Well Plate	snmC-seq34	Illumina Novaseq	3745514	2459192	65.7%	68.3%	63.0%	1844154	75.0%	1560670	84.6%	0.00694	0.75604	0.02544	0.75434	0.01863	6.44
171213_CEMBA_mm_P56_P63_3C_MOp_CEMBA171207_3C_1_CEMBA171207_3C_2_A10_AD008	Mus musculus	Primary motor cort	NeuN+	Zymo-Spin 384 Well Plate	snmC-seq35	Illumina Novaseq	2646966	1788766	67.6%	70.4%	64.8%	1360076	76.0%	1147193	84.3%	0.00848	0.78598	0.03118	0.78415	0.02289	4.75
171213_CEMBA_mm_P56_P63_3C_MOp_CEMBA171207_3C_1_CEMBA171207_3C_2_A10_AD010	Mus musculus	Primary motor cort	NeuN+	Zymo-Spin 384 Well Plate	snmC-seq36	Illumina Novaseq	5261116	3514155	66.8%	69.6%	64.0%	2640898	75.2%	2211393	83.7%	0.00759	0.76706	0.02412	0.76528	0.01666	9.02
171213_CEMBA_mm_P56_P63_3C_MOp_CEMBA171207_3C_1_CEMBA171207_3C_2_A10_AD012	Mus musculus	Primary motor cort	NeuN+	Zymo-Spin 384 Well Plate	snmC-seq37	Illumina Novaseq	4883860	3263799	66.8%	69.8%	63.9%	2446615	75.0%	2039236	83.3%	0.0086	0.80642	0.03003	0.80474	0.02162	8.36
171213_CEMBA_mm_P56_P63_3C_MOp_CEMBA171207_3C_1_CEMBA171207_3C_2_A11_AD001	Mus musculus	Primary motor cort	NeuN+	Zymo-Spin 384 Well Plate	snmC-seq38	Illumina Novaseq	5192688	3485451	67.1%	70.0%	64.3%	2464677	70.7%	2134026	86.6%	0.00566	0.74986	0.01906	0.74844	0.01348	8.65
171213_CEMBA_mm_P56_P63_3C_MOp_CEMBA171207_3C_1_CEMBA171207_3C_2_A11_AD002	Mus musculus	Primary motor cort	NeuN+	Zymo-Spin 384 Well Plate	snmC-seq39	Illumina Novaseq	3822654	2658503	69.5%	72.8%	66.3%	1889979	71.1%	1633228	86.4%	0.00855	0.77993	0.03524	0.77803	0.02692	6.69
171213_CEMBA_mm_P56_P63_3C_MOp_CEMBA171207_3C_1_CEMBA171207_3C_2_A11_AD004	Mus musculus	Primary motor cort	NeuN+	Zymo-Spin 384 Well Plate	snmC-seq40	Illumina Novaseq	6547524	4344264	66.3%	68.9%	63.8%	3063180	70.5%	2623393	85.6%	0.00742	0.76877	0.02742	0.76704	0.02015	10.55
171213_CEMBA_mm_P56_P63_3C_MOp_CEMBA171207_3C_1_CEMBA171207_3C_2_A11_AD007	Mus musculus	Primary motor cort	NeuN+	Zymo-Spin 384 Well Plate	snmC-seq41	Illumina Novaseq	4629218	2848834	61.5%	64.1%	59.0%	1987372	69.8%	1693069	85.2%	0.00808	0.77091	0.03125	0.76904	0.02336	6.9
171213_CEMBA_mm_P56_P63_3C_MOp_CEMBA171207_3C_1_CEMBA171207_3C_2_A11_AD010	Mus musculus	Primary motor cort	NeuN+	Zymo-Spin 384 Well Plate	snmC-seq42	Illumina Novaseq	5082694	3393446	66.8%	69.5%	64.0%	2393129	70.5%	2016566	84.3%	0.00708	0.76172	0.02238	0.76002	0.01541	8.15
171213_CEMBA_mm_P56_P63_3C_MOp_CEMBA171207_3C_1_CEMBA171207_3C_2_A11_AD012	Mus musculus	Primary motor cort	NeuN+	Zymo-Spin 384 Well Plate	snmC-seq43	Illumina Novaseq	4759428	3209597	67.4%	70.3%	64.6%	2264668	70.6%	1900363	83.9%	0.00685	0.77064	0.02288	0.76906	0.01614	7.73
171213_CEMBA_mm_P56_P63_3C_MOp_CEMBA171207_3C_1_CEMBA171207_3C_2_A12_AD004	Mus musculus	Primary motor cort	NeuN-	Zymo-Spin 384 Well Plate	snmC-seq44	Illumina Novaseq	5707692	3613236	63.3%	65.1%	61.5%	2644050	73.2%	2296188	86.8%	0.00592	0.73929	0.01188	0.73774	0.006	9.14
171213_CEMBA_mm_P56_P63_3C_MOp_CEMBA171207_3C_1_CEMBA171207_3C_2_A12_AD006	Mus musculus	Primary motor cort	NeuN+	Zymo-Spin 384 Well Plate	snmC-seq45	Illumina Novaseq	7259892	4655566	64.1%	65.9%	62.3%	3443444	74.0%	2972441	86.3%	0.00603	0.73931	0.01256	0.73773	0.00657	11.71
171213_CEMBA_mm_P56_P63_3C_MOp_CEMBA171207_3C_1_CEMBA171207_3C_2_A12_AD007	Mus musculus	Primary motor cort	NeuN+	Zymo-Spin 384 Well Plate	snmC-seq46	Illumina Novaseq	4105118	2699265	65.8%	67.8%	63.7%	1971889	73.1%	1691885	85.8%	0.00503	0.7044	0.00745	0.70291	0.00243	6.79
171213_CEMBA_mm_P56_P63_3C_MOp_CEMBA171207_3C_1_CEMBA171207_3C_2_A12_AD010	Mus musculus	Primary motor cort	NeuN+	Zymo-Spin 384 Well Plate	snmC-seq47	Illumina Novaseq	5427336	3631412	66.9%	69.0%	64.8%	2671248	73.6%	2264462	84.8%	0.00598	0.68277	0.00874	0.68086	0.00278	9.02
171213_CEMBA_mm_P56_P63_3C_MOp_CEMBA171207_3C_1_CEMBA171207_3C_2_A2_AD001	Mus musculus	Primary motor cort	NeuN+	Zymo-Spin 384 Well Plate	snmC-seq48	Illumina Novaseq	3476192	2287210	65.8%	68.4%	63.2%	1738946	76.0%	1522915	87.6%	0.00871	0.8084	0.03827	0.80672	0.02982	6.38
171213_CEMBA_mm_P56_P63_3C_MOp_CEMBA171207_3C_1_CEMBA171207_3C_2_A2_AD007	Mus musculus	Primary motor cort	NeuN+	Zymo-Spin 384 Well Plate	snmC-seq49	Illumina Novaseq	2685480	1772026	66.0%	68.2%	63.8%	1336875	75.4%	1152781	86.2%	0.00756	0.80896	0.02977	0.8075	0.02238	4.84
171213_CEMBA_mm_P56_P63_3C_MOp_CEMBA171207_3C_1_CEMBA171207_3C_2_A2_AD008	Mus musculus	Primary motor cort	NeuN+	Zymo-Spin 384 Well Plate	snmC-seq50	Illumina Novaseq	3658858	2397063	65.5%	67.8%	63.3%	1829748	76.3%	1566500	85.6%	0.00894	0.77803	0.03192	0.77603	0.02319	6.54
171219_CEMBA_mm_P56_P63_4B_CEMBA171213_4B_1_CEMBA171213_4B_2_A1_AD001_indexed	Mus musculus	Primary motor cort	NeuN+	Zymo-Spin 384 Well Plate	snmC-seq51	Illumina Novaseq	5094730	3222886	63.3%	66.2%	60.4%	2526896	78.4%	2107221	83.4%	0.0063	0.78514	0.02309	0.78378	0.0169	8.27
171219_CEMBA_mm_P56_P63_4B_CEMBA171213_4B_1_CEMBA171213_4B_2_A1_AD002_indexed	Mus musculus	Primary motor cort	NeuN+	Zymo-Spin 384 Well Plate	snmC-seq52	Illumina Novaseq	4848950	3043893	62.8%	65.6%	59.9%	2383972	78.3%	1969604	82.6%	0.00656	0.77167	0.02193	0.77016	0.01547	7.76
171219_CEMBA_mm_P56_P63_4B_CEMBA171213_4B_1_CEMBA171213_4B_2_A1_AD004_indexed	Mus musculus	Primary motor cort	NeuN+	Zymo-Spin 384 Well Plate	snmC-seq53	Illumina Novaseq	4974236	3158598	63.5%	66.1%	60.9%	2469583	78.2%	2041281	82.7%	0.00769	0.82069	0.0321	0.8193	0.0246	8
171219_CEMBA_mm_P56_P63_4B_CEMBA171213_4B_1_CEMBA171213_4B_2_A1_AD006_indexed	Mus musculus	Primary motor cort	NeuN+	Zymo-Spin 384 Well Plate	snmC-seq54	Illumina Novaseq	4789508	2958376	61.8%	64.2%	59.4%	2318990	78.4%	1910484	82.4%	0.00876	0.82291	0.03789	0.82134	0.02939	7.5
171219_CEMBA_mm_P56_P63_4B_CEMBA171213_4B_1_CEMBA171213_4B_2_A1_AD007_indexed	Mus musculus	Primary motor cort	NeuN+	Zymo-Spin 384 Well Plate	snmC-seq55	Illumina Novaseq	4478150	2873957	64.2%	66.6%	61.7%	2235753	77.8%	1834102	82.0%	0.00582	0.77591	0.01917	0.7746	0.01343	7.2
171219_CEMBA_mm_P56_P63_4B_CEMBA171213_4B_1_CEMBA171213_4B_2_A1_AD008_indexed	Mus musculus	Primary motor cort	NeuN+	Zymo-Spin 384 Well Plate	snmC-seq56	Illumina Novaseq	5462550	3483633	63.8%	66.6%	60.9%	2718227	78.0%	2218669	81.6%	0.00869	0.84506	0.03664	0.8437	0.0282	8.66
171219_CEMBA_mm_P56_P63_4B_CEMBA171213_4B_1_CEMBA171213_4B_2_A1_AD010_indexed	Mus musculus	Primary motor cort	NeuN+	Zymo-Spin 384 Well Plate	snmC-seq57	Illumina Novaseq	5390732	3353765	62.2%	64.7%	59.7%	2602481	77.6%	2105689	80.9%	0.00759	0.7815	0.03048	0.77983	0.02307	8.21
171219_CEMBA_mm_P56_P63_4B_CEMBA171213_4B_1_CEMBA171213_4B_2_A1_AD012_indexed	Mus musculus	Primary motor cort	NeuN+	Zymo-Spin 384 Well Plate	snmC-seq58	Illumina Novaseq	2402228	1403422	58.4%	60.7%	56.2%	1102438	78.6%	891671	80.9%	0.00857	0.8142	0.03438	0.81259	0.02603	3.53
171219_CEMBA_mm_P56_P63_4B_CEMBA171213_4B_1_CEMBA171213_4B_2_A10_AD001_indexed	Mus musculus	Primary motor cort	NeuN-	Zymo-Spin 384 Well Plate	snmC-seq59	Illumina Novaseq	4055598	2524586	62.2%	65.9%	58.6%	1995897	79.1%	1645122	82.4%	0.00724	0.81856	0.03121	0.81724	0.02414	6.61
171219_CEMBA_mm_P56_P63_4B_CEMBA171213_4B_1_CEMBA171213_4B_2_A10_AD002_indexed	Mus musculus	Primary motor cort	NeuN+	Zymo-Spin 384 Well Plate	snmC-seq60	Illumina Novaseq	3362188	2110925	62.8%	66.3%	59.3%	1683726	79.8%	1378941	81.9%	0.00631	0.7727	0.02124	0.77126	0.01502	5.57
171219_CEMBA_mm_P56_P63_4B_CEMBA171213_4B_1_CEMBA171213_4B_2_A10_AD004_indexed	Mus musculus	Primary motor cort	NeuN-	Zymo-Spin 384 Well Plate	snmC-seq61	Illumina Novaseq	3544558	2246875	63.4%	66.6%	60.2%	1789224	79.6%	1452463	81.2%	0.00634	0.75479	0.02015	0.75323	0.0139	5.8
171219_CEMBA_mm_P56_P63_4B_CEMBA171213_4B_1_CEMBA171213_4B_2_A10_AD006_indexed	Mus musculus	Primary motor cort	NeuN+	Zymo-Spin 384 Well Plate	snmC-seq62	Illumina Novaseq	5832512	3623976	62.1%	65.3%	59.0%	2879783	79.5%	2326866	80.8%	0.00786	0.78612	0.03032	0.78443	0.02264	9.2
171219_CEMBA_mm_P56_P63_4B_CEMBA171213_4B_1_CEMBA171213_4B_2_A10_AD007_indexed	Mus musculus	Primary motor cort	NeuN+	Zymo-Spin 384 Well Plate	snmC-seq63	Illumina Novaseq	5319458	3410468	64.1%	67.4%	60.8%	2700386	79.2%	2158564	79.9%	0.00705	0.80678	0.02644	0.80541	0.01953	8.55
171219_CEMBA_mm_P56_P63_4B_CEMBA171213_4B_1_CEMBA171213_4B_2_A10_AD008_indexed	Mus musculus	Primary motor cort	NeuN+	Zymo-Spin 384 Well Plate	snmC-seq64	Illumina Novaseq	4501140	2828200	62.8%	66.2%	59.4%	2238867	79.2%	1779414	79.5%	0.00825	0.77598	0.03389	0.77412	0.02585	7.06
171219_CEMBA_mm_P56_P63_4B_CEMBA171213_4B_1_CEMBA171213_4B_2_A10_AD012_indexed	Mus musculus	Primary motor cort	NeuN+	Zymo-Spin 384 Well Plate	snmC-seq65	Illumina Novaseq	5490104	3521921	64.2%	67.5%	60.8%	2797120	79.4%	2190787	78.3%	0.00876	0.81854	0.03745	0.81694	0.02894	8.63
171219_CEMBA_mm_P56_P63_4B_CEMBA171213_4B_1_CEMBA171213_4B_2_A12_AD002_indexed	Mus musculus	Primary motor cort	NeuN+	Zymo-Spin 384 Well Plate	snmC-seq66	Illumina Novaseq	5516246	3490959	63.3%	66.8%	59.8%	2567203	73.5%	2103736	81.9%	0.00613	0.77257	0.01624	0.77117	0.01017	8.29
171219_CEMBA_mm_P56_P63_4B_CEMBA171213_4B_1_CEMBA171213_4B_2_A12_AD004_indexed	Mus musculus	Primary motor cort	NeuN+	Zymo-Spin 384 Well Plate	snmC-seq67	Illumina Novaseq	8221852	5216239	63.4%	66.6%	60.3%	3801431	72.9%	3117077	82.0%	0.00552	0.73857	0.01315	0.73712	0.00767	11.98
171219_CEMBA_mm_P56_P63_4B_CEMBA171213_4B_1_CEMBA171213_4B_2_A12_AD007_indexed	Mus musculus	Primary motor cort	NeuN+	Zymo-Spin 384 Well Plate	snmC-seq68	Illumina Novaseq	14897080	9532442	64.0%	67.1%	60.8%	6888098	72.3%	5598520	81.3%	0.00451	0.71797	0.00844	0.71669	0.00395	20.32
171219_CEMBA_mm_P56_P63_4B_CEMBA171213_4B_1_CEMBA171213_4B_2_A12_AD008_indexed	Mus musculus	Primary motor cort	NeuN+	Zymo-Spin 384 Well Plate	snmC-seq69	Illumina Novaseq	8398856	5241226	62.4%	65.6%	59.2%	3806088	72.6%	3064968	80.5%	0.00541	0.74844	0.01096	0.74707	0.00558	11.74
171219_CEMBA_mm_P56_P63_4B_CEMBA171213_4B_1_CEMBA171213_4B_2_A2_AD001_indexed	Mus musculus	Primary motor cort	NeuN+	Zymo-Spin 384 Well Plate	snmC-seq70	Illumina Novaseq	3834942	2363136	61.6%	65.1%	58.2%	1823243	77.2%	1508177	82.7%	0.00629	0.76291	0.02265	0.76141	0.01646	6.02
171219_CEMBA_mm_P56_P63_4B_CEMBA171213_4B_1_CEMBA171213_4B_2_A2_AD002_indexed	Mus musculus	Primary motor cort	NeuN+	Zymo-Spin 384 Well Plate	snmC-seq71	Illumina Novaseq	4018736	2568788	63.9%	67.4%	60.5%	1987241	77.4%	1643288	82.7%	0.0078	0.79462	0.03079	0.79301	0.02317	6.58
171219_CEMBA_mm_P56_P63_4B_CEMBA171213_4B_1_CEMBA171213_4B_2_A2_AD004_indexed	Mus musculus	Primary motor cort	NeuN+	Zymo-Spin 384 Well Plate	snmC-seq72	Illumina Novaseq	4632848	2682423	57.9%	60.8%	55.0%	2064313	77.0%	1703269	82.5%	0.00869	0.79087	0.03619	0.78904	0.02774	6.77
171219_CEMBA_mm_P56_P63_4B_CEMBA171213_4B_1_CEMBA171213_4B_2_A2_AD006_indexed	Mus musculus	Primary motor cort	NeuN+	Zymo-Spin 384 Well Plate	snmC-seq73	Illumina Novaseq	5493608	3402223	61.9%	65.0%	58.9%	2625931	77.2%	2149220	81.8%	0.00993	0.826	0.04568	0.82425	0.03611	8.49
171219_CEMBA_mm_P56_P63_4B_CEMBA171213_4B_1_CEMBA171213_4B_2_A2_AD007_indexed	Mus musculus	Primary motor cort	NeuN+	Zymo-Spin 384 Well Plate	snmC-seq74	Illumina Novaseq	4034390	2575971	63.9%	66.9%	60.8%	1977229	76.8%	1605265	81.2%	0.00739	0.799	0.02983	0.7975	0.02261	6.39
180118_CEMBA_mm_P56_P63_4B_CEMBA180104_4B_1_CEMBA180104_4B_2_A1_AD001_indexed	Mus musculus	Primary motor cort	NeuN+	Zymo-Spin 384 Well Plate	snmC-seq75	Illumina Novaseq	3780328	2513854	66.5%	69.3%	63.7%	1997072	79.4%	1706585	85.5%	0.00508	0.76379	0.02117	0.76258	0.01617	7.16
180118_CEMBA_mm_P56_P63_4B_CEMBA180104_4B_1_CEMBA180104_4B_2_A1_AD002_indexed	Mus musculus	Primary motor cort	NeuN+	Zymo-Spin 384 Well Plate	snmC-seq76	Illumina Novaseq	3119584	2024552	64.9%	68.0%	61.8%	1605075	79.3%	1363293	84.9%	0.00615	0.75156	0.02543	0.75002	0.0194	5.75
180118_CEMBA_mm_P56_P63_4B_CEMBA180104_4B_1_CEMBA180104_4B_2_A1_AD004_indexed	Mus musculus	Primary motor cort	NeuN+	Zymo-Spin 384 Well Plate	snmC-seq77	Illumina Novaseq	1557762	1069256	68.6%	71.2%	66.1%	863651	80.8%	733545	84.9%	0.00568	0.79315	0.02452	0.79197	0.01895	3.12
180118_CEMBA_mm_P56_P63_4B_CEMBA180104_4B_1_CEMBA180104_4B_2_A1_AD006_indexed	Mus musculus	Primary motor cort	NeuN+	Zymo-Spin 384 Well Plate	snmC-seq78	Illumina Novaseq	3506930	2270421	64.7%	66.8%	62.7%	1813353	79.9%	1528999	84.3%	0.00506	0.77165	0.01804	0.77049	0.01305	6.44
180118_CEMBA_mm_P56_P63_4B_CEMBA180104_4B_1_CEMBA180104_4B_2_A1_AD007_indexed	Mus musculus	Primary motor cort	NeuN+	Zymo-Spin 384 Well Plate	snmC-seq79	Illumina Novaseq	2216452	1472481	66.4%	68.9%	64.0%	1173187	79.7%	970282	82.7%	0.00601	0.78934	0.02205	0.78807	0.01614	4.11
180118_CEMBA_mm_P56_P63_4B_CEMBA180104_4B_1_CEMBA180104_4B_2_A10_AD001_indexed	Mus musculus	Primary motor cort	NeuN+	Zymo-Spin 384 Well Plate	snmC-seq80	Illumina Novaseq	2849002	1915099	67.2%	69.5%	65.0%	1531205	80.0%	1313244	85.8%	0.00533	0.76296	0.02159	0.76169	0.01635	5.54
180118_CEMBA_mm_P56_P63_4B_CEMBA180104_4B_1_CEMBA180104_4B_2_A10_AD002_indexed	Mus musculus	Primary motor cort	NeuN+	Zymo-Spin 384 Well Plate	snmC-seq81	Illumina Novaseq	2668144	1806057	67.7%	70.2%	65.2%	1437251	79.6%	1221188	85.0%	0.00536	0.76765	0.02011	0.7664	0.01483	5.16
180118_CEMBA_mm_P56_P63_4B_CEMBA180104_4B_1_CEMBA180104_4B_2_A10_AD004_indexed	Mus musculus	Primary motor cort	NeuN+	Zymo-Spin 384 Well Plate	snmC-seq82	Illumina Novaseq	2948226	1979091	67.1%	69.3%	65.0%	1583888	80.0%	1343299	84.8%	0.00667	0.8242	0.03092	0.82302	0.02441	5.67
180118_CEMBA_mm_P56_P63_4B_CEMBA180104_4B_1_CEMBA180104_4B_2_A10_AD006_indexed	Mus musculus	Primary motor cort	NeuN+	Zymo-Spin 384 Well Plate	snmC-seq83	Illumina Novaseq	3325384	2157185	64.9%	66.7%	63.0%	1724891	80.0%	1449858	84.1%	0.00585	0.76718	0.02285	0.76581	0.0171	6.1
180118_CEMBA_mm_P56_P63_4B_CEMBA180104_4B_1_CEMBA180104_4B_2_A10_AD007_indexed	Mus musculus	Primary motor cort	NeuN+	Zymo-Spin 384 Well Plate	snmC-seq84	Illumina Novaseq	3009456	2020573	67.1%	69.4%	64.9%	1606602	79.5%	1331633	82.9%	0.00655	0.8224	0.03233	0.82123	0.02595	5.61
180118_CEMBA_mm_P56_P63_4B_CEMBA180104_4B_1_CEMBA180104_4B_2_A10_AD008_indexed	Mus musculus	Primary motor cort	NeuN+	Zymo-Spin 384 Well Plate	snmC-seq85	Illumina Novaseq	3060472	2030880	66.4%	68.4%	64.3%	1627208	80.1%	1351021	83.0%	0.00632	0.83852	0.02675	0.83749	0.02056	5.69
180118_CEMBA_mm_P56_P63_4B_CEMBA180104_4B_1_CEMBA180104_4B_2_A10_AD010_indexed	Mus musculus	Primary motor cort	NeuN+	Zymo-Spin 384 Well Plate	snmC-seq86	Illumina Novaseq	2708110	1800181	66.5%	68.5%	64.4%	1444531	80.2%	1178336	81.6%	0.00524	0.77354	0.02026	0.77235	0.0151	4.97
180118_CEMBA_mm_P56_P63_4B_CEMBA180104_4B_1_CEMBA180104_4B_2_A10_AD012_indexed	Mus musculus	Primary motor cort	NeuN+	Zymo-Spin 384 Well Plate	snmC-seq87	Illumina Novaseq	2472076	1697509	68.7%	71.0%	66.3%	1363937	80.3%	1124594	82.5%	0.0083	0.78422	0.03925	0.78241	0.03121	4.76
180118_CEMBA_mm_P56_P63_4B_CEMBA180104_4B_1_CEMBA180104_4B_2_A11_AD001_indexed	Mus musculus	Primary motor cort	NeuN+	Zymo-Spin 384 Well Plate	snmC-seq88	Illumina Novaseq	3346326	2218120	66.3%	69.4%	63.2%	1745124	78.7%	1486702	85.2%	0.00619	0.79632	0.02794	0.79505	0.02189	6.25
180118_CEMBA_mm_P56_P63_4B_CEMBA180104_4B_1_CEMBA180104_4B_2_A11_AD004_indexed	Mus musculus	Primary motor cort	NeuN+	Zymo-Spin 384 Well Plate	snmC-seq89	Illumina Novaseq	2585186	1702652	65.9%	68.6%	63.2%	1337070	78.5%	1131708	84.6%	0.00568	0.7591	0.02334	0.75772	0.01776	4.78
180118_CEMBA_mm_P56_P63_4B_CEMBA180104_4B_1_CEMBA180104_4B_2_A11_AD006_indexed	Mus musculus	Primary motor cort	NeuN-	Zymo-Spin 384 Well Plate	snmC-seq90	Illumina Novaseq	4165464	2672308	64.2%	66.6%	61.7%	2110117	79.0%	1765077	83.6%	0.00641	0.79021	0.02632	0.78886	0.02004	7.38
180118_CEMBA_mm_P56_P63_4B_CEMBA180104_4B_1_CEMBA180104_4B_2_A11_AD007_indexed	Mus musculus	Primary motor cort	NeuN-	Zymo-Spin 384 Well Plate	snmC-seq91	Illumina Novaseq	3492296	2332171	66.8%	69.5%	64.0%	1841861	79.0%	1518761	82.5%	0.00486	0.76919	0.01876	0.76806	0.01397	6.37
180118_CEMBA_mm_P56_P63_4B_CEMBA180104_4B_1_CEMBA180104_4B_2_A11_AD008_indexed	Mus musculus	Primary motor cort	NeuN-	Zymo-Spin 384 Well Plate	snmC-seq92	Illumina Novaseq	3325180	2205872	66.3%	69.0%	63.6%	1734465	78.6%	1449950	83.6%	0.00822	0.8262	0.04211	0.82476	0.03417	6.1
180118_CEMBA_mm_P56_P63_4B_CEMBA180104_4B_1_CEMBA180104_4B_2_A11_AD010_indexed	Mus musculus	Primary motor cort	NeuN-	Zymo-Spin 384 Well Plate	snmC-seq93	Illumina Novaseq	3489256	2303321	66.0%	68.8%	63.2%	1811261	78.6%	1492812	82.4%	0.00564	0.77946	0.02285	0.77821	0.01731	6.27
180118_CEMBA_mm_P56_P63_4B_CEMBA180104_4B_1_CEMBA180104_4B_2_A11_AD012_indexed	Mus musculus	Primary motor cort	NeuN+	Zymo-Spin 384 Well Plate	snmC-seq94	Illumina Novaseq	2814418	1877112	66.7%	69.7%	63.7%	1483436	79.0%	1218723	82.2%	0.00611	0.79283	0.02487	0.79156	0.01888	5.15
180118_CEMBA_mm_P56_P63_4B_CEMBA180104_4B_1_CEMBA180104_4B_2_A12_AD006_indexed	Mus musculus	Primary motor cort	NeuN+	Zymo-Spin 384 Well Plate	snmC-seq95	Illumina Novaseq	6661586	4070117	61.1%	66.6%	55.6%	2983412	73.3%	2418126	81.1%	0.00386	0.743	0.00655	0.742	0.0027	9.84
180118_CEMBA_mm_P56_P63_4B_CEMBA180104_4B_1_CEMBA180104_4B_2_A12_AD007_indexed	Mus musculus	Primary motor cort	NeuN+	Zymo-Spin 384 Well Plate	snmC-seq96	Illumina Novaseq	4553980	2894947	63.6%	69.6%	57.5%	2116033	73.1%	1707601	80.7%	0.00384	0.74135	0.01	0.74035	0.00618	7.03
180118_CEMBA_mm_P56_P63_4B_CEMBA180104_4B_1_CEMBA180104_4B_2_A12_AD008_indexed	Mus musculus	Primary motor cort	NeuN+	Zymo-Spin 384 Well Plate	snmC-seq97	Illumina Novaseq	5301632	3301530	62.3%	67.9%	56.6%	2396327	72.6%	1926895	80.4%	0.00392	0.73206	0.00914	0.73101	0.00524	7.89
180118_CEMBA_mm_P56_P63_4B_CEMBA180104_4B_1_CEMBA180104_4B_2_A12_AD010_indexed	Mus musculus	Primary motor cort	NeuN+	Zymo-Spin 384 Well Plate	snmC-seq98	Illumina Novaseq	4838528	3051783	63.1%	68.6%	57.5%	2215577	72.6%	1763389	79.6%	0.00422	0.73005	0.0108	0.72891	0.00661	7.24

Supplementary Table 2. Sequence of library amplification primers for unique dual-indexing

P5 primer sequence	P7 primer sequence	P5 index sequence (10bp)	P7 index sequence (10bp)
AATGATACGGCGACCACCGAGATCTACACACGATCAGACACACTCTTTCCCTACACGACGCTCT	CAAGCAGAAGACGGCATACGAGATAGGCAATGGTGACTGGAGTTCAGACGTGTGCTCTT	ACGATCAGAC	CATTGCCTAT
AATGATACGGCGACCACCGAGATCTACACTCGAGAGTACACTCTTTCCCTACACGACGCTCT	CAAGCAGAAGACGGCATACGAGATTCACCTAGGTGACTGGAGTTCAGACGTGTGCTCTT	TCGAGAGTAC	CTAGGTGAAT
AATGATACGGCGACCACCGAGATCTACACCTAGCTCAACACTCTTTCCCTACACGACGCTCT	CAAGCAGAAGACGGCATACGAGATCATACGGAGTGACTGGAGTTCAGACGTGTGCTCTT	CTAGCTCAAC	TCCGTATGAT
AATGATACGGCGACCACCGAGATCTACACATCGTCTCACACTCTTTCCCTACACGACGCTCT	CAAGCAGAAGACGGCATACGAGATGTCATCGTGTGACTGGAGTTCAGACGTGTGCTCTT	ATCGTCTCAC	ACGATGACAT
AATGATACGGCGACCACCGAGATCTACACTCGACAAGACACTCTTTCCCTACACGACGCTCT	CAAGCAGAAGACGGCATACGAGATTTACCGACGTGACTGGAGTTCAGACGTGTGCTCTT	TCGACAAGAC	GTCGGTAAAT
AATGATACGGCGACCACCGAGATCTACACCCTTGGAAACACTCTTTCCCTACACGACGCTCT	CAAGCAGAAGACGGCATACGAGATACCTTCGAGTGACTGGAGTTCAGACGTGTGCTCTT	CCTTGGAAAC	TCGAAGGTAT
AATGATACGGCGACCACCGAGATCTACACATCATGCGACACTCTTTCCCTACACGACGCTCT	CAAGCAGAAGACGGCATACGAGATACGCTTCTGTGACTGGAGTTCAGACGTGTGCTCTT	ATCATGCGAC	AGAAGCGTAT
AATGATACGGCGACCACCGAGATCTACACTGTTCCGTACACTCTTTCCCTACACGACGCTCT	CAAGCAGAAGACGGCATACGAGATGAGTAGAGGTGACTGGAGTTCAGACGTGTGCTCTT	TGTTCCGTAC	CTCTACTCAT
AATGATACGGCGACCACCGAGATCTACACATTAGCCGACACTCTTTCCCTACACGACGCTCT	CAAGCAGAAGACGGCATACGAGATATGCCTAGGTGACTGGAGTTCAGACGTGTGCTCTT	ATTAGCCGAC	CTAGGCATAT
AATGATACGGCGACCACCGAGATCTACACCGATCGATACACTCTTTCCCTACACGACGCTCT	CAAGCAGAAGACGGCATACGAGATCAACTCCAGTGACTGGAGTTCAGACGTGTGCTCTT	CGATCGATAC	TGGAGTTGAT
AATGATACGGCGACCACCGAGATCTACACGATCTTGCACACTCTTTCCCTACACGACGCTCT	CAAGCAGAAGACGGCATACGAGATAAGTCCTCGTGACTGGAGTTCAGACGTGTGCTCTT	GATCTTGCAC	GAGGACTTAT
AATGATACGGCGACCACCGAGATCTACACAGGATAGCACACTCTTTCCCTACACGACGCTCT	CAAGCAGAAGACGGCATACGAGATGTCGATTGGTGACTGGAGTTCAGACGTGTGCTCTT	AGGATAGCAC	CAATCGACAT
AATGATACGGCGACCACCGAGATCTACACGTAGCGTAACACTCTTTCCCTACACGACGCTCT	CAAGCAGAAGACGGCATACGAGATGCGTTAGAGTGACTGGAGTTCAGACGTGTGCTCTT	GTAGCGTAAC	TCTAACGCAT
AATGATACGGCGACCACCGAGATCTACACAGAGTCCAACACTCTTTCCCTACACGACGCTCT	CAAGCAGAAGACGGCATACGAGATTTGCGAGAGTGACTGGAGTTCAGACGTGTGCTCTT	AGAGTCCAAC	TCTCGCAAAT
AATGATACGGCGACCACCGAGATCTACACGCTACTCTACACTCTTTCCCTACACGACGCTCT	CAAGCAGAAGACGGCATACGAGATACACCGATGTGACTGGAGTTCAGACGTGTGCTCTT	GCTACTCTAC	ATCGGTGTAT
AATGATACGGCGACCACCGAGATCTACACCTCTGGATACACTCTTTCCCTACACGACGCTCT	CAAGCAGAAGACGGCATACGAGATCGTATCTCGTGACTGGAGTTCAGACGTGTGCTCTT	CTCTGGATAC	GAGATACGAT
AATGATACGGCGACCACCGAGATCTACACAGATCGTCACACTCTTTCCCTACACGACGCTCT	CAAGCAGAAGACGGCATACGAGATAAGGAGACGTGACTGGAGTTCAGACGTGTGCTCTT	AGATCGTCAC	GTCTCCTTAT
AATGATACGGCGACCACCGAGATCTACACGCTCAGTTACACTCTTTCCCTACACGACGCTCT	CAAGCAGAAGACGGCATACGAGATTGTCGACTGTGACTGGAGTTCAGACGTGTGCTCTT	GCTCAGTTAC	AGTCGACAAT
AATGATACGGCGACCACCGAGATCTACACGTCCTAAGACACTCTTTCCCTACACGACGCTCT	CAAGCAGAAGACGGCATACGAGATTCAATCCGGTGACTGGAGTTCAGACGTGTGCTCTT	GTCCTAAGAC	CGGATTGAAT
AATGATACGGCGACCACCGAGATCTACACTATGGCACACACTCTTTCCCTACACGACGCTCT	CAAGCAGAAGACGGCATACGAGATGACTTGTGGTGACTGGAGTTCAGACGTGTGCTCTT	TATGGCACAC	CACAAGTCAT
AATGATACGGCGACCACCGAGATCTACACTCGGATTCACACTCTTTCCCTACACGACGCTCT	CAAGCAGAAGACGGCATACGAGATCCGATGTAGTGACTGGAGTTCAGACGTGTGCTCTT	TCGGATTCAC	TACATCGGAT
AATGATACGGCGACCACCGAGATCTACACAACAGCGAACACTCTTTCCCTACACGACGCTCT	CAAGCAGAAGACGGCATACGAGATTAGGAGCTGTGACTGGAGTTCAGACGTGTGCTCTT	AACAGCGAAC	AGCTCCTAAT
AATGATACGGCGACCACCGAGATCTACACCCAACGAAACACTCTTTCCCTACACGACGCTCT	CAAGCAGAAGACGGCATACGAGATCAACGAGTGTGACTGGAGTTCAGACGTGTGCTCTT	CCAACGAAAC	ACTCGTTGAT
AATGATACGGCGACCACCGAGATCTACACCAGTGCTTACACTCTTTCCCTACACGACGCTCT	CAAGCAGAAGACGGCATACGAGATTGTGTCAGGTGACTGGAGTTCAGACGTGTGCTCTT	CAGTGCTTAC	CTGACACAAT
AATGATACGGCGACCACCGAGATCTACACGATCAAGGACACTCTTTCCCTACACGACGCTCT	CAAGCAGAAGACGGCATACGAGATCTAGGTTGGTGACTGGAGTTCAGACGTGTGCTCTT	GATCAAGGAC	CAACCTAGAT
AATGATACGGCGACCACCGAGATCTACACTCTTCGACACACTCTTTCCCTACACGACGCTCT	CAAGCAGAAGACGGCATACGAGATGTGTCCTTGTGACTGGAGTTCAGACGTGTGCTCTT	TCTTCGACAC	AAGGACACAT
AATGATACGGCGACCACCGAGATCTACACATCGTGGTACACTCTTTCCCTACACGACGCTCT	CAAGCAGAAGACGGCATACGAGATTACCTGCAGTGACTGGAGTTCAGACGTGTGCTCTT	ATCGTGGTAC	TGCAGGTAAT
AATGATACGGCGACCACCGAGATCTACACCGGTAATCACACTCTTTCCCTACACGACGCTCT	CAAGCAGAAGACGGCATACGAGATCCTTAGGTGTGACTGGAGTTCAGACGTGTGCTCTT	CGGTAATCAC	ACCTAAGGAT
AATGATACGGCGACCACCGAGATCTACACAGTTGTGCACACTCTTTCCCTACACGACGCTCT	CAAGCAGAAGACGGCATACGAGATCACAGACTGTGACTGGAGTTCAGACGTGTGCTCTT	AGTTGTGCAC	AGTCTGTGAT
AATGATACGGCGACCACCGAGATCTACACAATGACGCACACTCTTTCCCTACACGACGCTCT	CAAGCAGAAGACGGCATACGAGATTCGAACCTGTGACTGGAGTTCAGACGTGTGCTCTT	AATGACGCAC	AGGTTCGAAT
AATGATACGGCGACCACCGAGATCTACACTACCGGATACACTCTTTCCCTACACGACGCTCT	CAAGCAGAAGACGGCATACGAGATGCATAGTCGTGACTGGAGTTCAGACGTGTGCTCTT	TACCGGATAC	GACTATGCAT
AATGATACGGCGACCACCGAGATCTACACTTGCAACGACACTCTTTCCCTACACGACGCTCT	CAAGCAGAAGACGGCATACGAGATCTCCTGAAGTGACTGGAGTTCAGACGTGTGCTCTT	TTGCAACGAC	TTCAGGAGAT
AATGATACGGCGACCACCGAGATCTACACCACTTCACACACTCTTTCCCTACACGACGCTCT	CAAGCAGAAGACGGCATACGAGATAACGCACAGTGACTGGAGTTCAGACGTGTGCTCTT	CACTTCACAC	TGTGCGTTAT
AATGATACGGCGACCACCGAGATCTACACTAGCCATGACACTCTTTCCCTACACGACGCTCT	CAAGCAGAAGACGGCATACGAGATTAGTCTCGGTGACTGGAGTTCAGACGTGTGCTCTT	TAGCCATGAC	CGAGACTAAT
AATGATACGGCGACCACCGAGATCTACACACAGGCATACACTCTTTCCCTACACGACGCTCT	CAAGCAGAAGACGGCATACGAGATACTCTGAGGTGACTGGAGTTCAGACGTGTGCTCTT	ACAGGCATAC	CTCAGAGTAT
AATGATACGGCGACCACCGAGATCTACACAGGTGTTGACACTCTTTCCCTACACGACGCTCT	CAAGCAGAAGACGGCATACGAGATGTTATGGCGTGACTGGAGTTCAGACGTGTGCTCTT	AGGTGTTGAC	GCCATAACAT
AATGATACGGCGACCACCGAGATCTACACCAGTCACAACACTCTTTCCCTACACGACGCTCT	CAAGCAGAAGACGGCATACGAGATCTCGGTAAGTGACTGGAGTTCAGACGTGTGCTCTT	CAGTCACAAC	TTACCGAGAT
AATGATACGGCGACCACCGAGATCTACACTCGATGACACACTCTTTCCCTACACGACGCTCT	CAAGCAGAAGACGGCATACGAGATTACAGAGCGTGACTGGAGTTCAGACGTGTGCTCTT	TCGATGACAC	GCTCTGTAAT
AATGATACGGCGACCACCGAGATCTACACGAAGTGCTACACTCTTTCCCTACACGACGCTCT	CAAGCAGAAGACGGCATACGAGATGCATAACGGTGACTGGAGTTCAGACGTGTGCTCTT	GAAGTGCTAC	CGTTATGCAT
AATGATACGGCGACCACCGAGATCTACACCTTCCTTCACACTCTTTCCCTACACGACGCTCT	CAAGCAGAAGACGGCATACGAGATGATCAGACGTGACTGGAGTTCAGACGTGTGCTCTT	CTTCCTTCAC	GTCTGATCAT
AATGATACGGCGACCACCGAGATCTACACCGAACAACACACTCTTTCCCTACACGACGCTCT	CAAGCAGAAGACGGCATACGAGATCGCAACTAGTGACTGGAGTTCAGACGTGTGCTCTT	CGAACAACAC	TAGTTGCGAT
AATGATACGGCGACCACCGAGATCTACACAACAACCGACACTCTTTCCCTACACGACGCTCT	CAAGCAGAAGACGGCATACGAGATTCCGATCAGTGACTGGAGTTCAGACGTGTGCTCTT	AACAACCGAC	TGATCGGAAT
AATGATACGGCGACCACCGAGATCTACACACCTCAGTACACTCTTTCCCTACACGACGCTCT	CAAGCAGAAGACGGCATACGAGATCAACTTGGGTGACTGGAGTTCAGACGTGTGCTCTT	ACCTCAGTAC	CCAAGTTGAT
AATGATACGGCGACCACCGAGATCTACACCGTCTTCAACACTCTTTCCCTACACGACGCTCT	CAAGCAGAAGACGGCATACGAGATTCAGTAGGGTGACTGGAGTTCAGACGTGTGCTCTT	CGTCTTCAAC	CCTACTGAAT
AATGATACGGCGACCACCGAGATCTACACTGCGTAACACACTCTTTCCCTACACGACGCTCT	CAAGCAGAAGACGGCATACGAGATACAGCAAGGTGACTGGAGTTCAGACGTGTGCTCTT	TGCGTAACAC	CTTGCTGTAT
AATGATACGGCGACCACCGAGATCTACACAACACGCTACACTCTTTCCCTACACGACGCTCT	CAAGCAGAAGACGGCATACGAGATGAATGGCAGTGACTGGAGTTCAGACGTGTGCTCTT	AACACGCTAC	TGCCATTCAT
AATGATACGGCGACCACCGAGATCTACACACTCGATCACACTCTTTCCCTACACGACGCTCT	CAAGCAGAAGACGGCATACGAGATCGGATCAAGTGACTGGAGTTCAGACGTGTGCTCTT	ACTCGATCAC	TTGATCCGAT
AATGATACGGCGACCACCGAGATCTACACTGAGCTGTACACTCTTTCCCTACACGACGCTCT	CAAGCAGAAGACGGCATACGAGATACTGCACTGTGACTGGAGTTCAGACGTGTGCTCTT	TGAGCTGTAC	AGTGCAGTAT
AATGATACGGCGACCACCGAGATCTACACTACTGCTCACACTCTTTCCCTACACGACGCTCT	CAAGCAGAAGACGGCATACGAGATCCTAAGTCGTGACTGGAGTTCAGACGTGTGCTCTT	TACTGCTCAC	GACTTAGGAT
AATGATACGGCGACCACCGAGATCTACACGACGAACTACACTCTTTCCCTACACGACGCTCT	CAAGCAGAAGACGGCATACGAGATTTCGTACGGTGACTGGAGTTCAGACGTGTGCTCTT	GACGAACTAC	CGTACGAAAT
AATGATACGGCGACCACCGAGATCTACACCTTCGCAAACACTCTTTCCCTACACGACGCTCT	CAAGCAGAAGACGGCATACGAGATTCCTGGTAGTGACTGGAGTTCAGACGTGTGCTCTT	CTTCGCAAAC	TACCAGGAAT
AATGATACGGCGACCACCGAGATCTACACATGGCGATACACTCTTTCCCTACACGACGCTCT	CAAGCAGAAGACGGCATACGAGATCATTGACGGTGACTGGAGTTCAGACGTGTGCTCTT	ATGGCGATAC	CGTCAATGAT
AATGATACGGCGACCACCGAGATCTACACACATGCCAACACTCTTTCCCTACACGACGCTCT	CAAGCAGAAGACGGCATACGAGATACCTCTTCGTGACTGGAGTTCAGACGTGTGCTCTT	ACATGCCAAC	GAAGAGGTAT
AATGATACGGCGACCACCGAGATCTACACGTCAACAGACACTCTTTCCCTACACGACGCTCT	CAAGCAGAAGACGGCATACGAGATCATTCGTCGTGACTGGAGTTCAGACGTGTGCTCTT	GTCAACAGAC	GACGAATGAT
AATGATACGGCGACCACCGAGATCTACACGTGGTATGACACTCTTTCCCTACACGACGCTCT	CAAGCAGAAGACGGCATACGAGATTTCCTCCTGTGACTGGAGTTCAGACGTGTGCTCTT	GTGGTATGAC	AGGAGGAAAT
AATGATACGGCGACCACCGAGATCTACACCCAACTTCACACTCTTTCCCTACACGACGCTCT	CAAGCAGAAGACGGCATACGAGATGCTGTAAGGTGACTGGAGTTCAGACGTGTGCTCTT	CCAACTTCAC	CTTACAGCAT
AATGATACGGCGACCACCGAGATCTACACGACGTCATACACTCTTTCCCTACACGACGCTCT	CAAGCAGAAGACGGCATACGAGATGACATCTCGTGACTGGAGTTCAGACGTGTGCTCTT	GACGTCATAC	GAGATGTCAT
AATGATACGGCGACCACCGAGATCTACACACGTCCAAACACTCTTTCCCTACACGACGCTCT	CAAGCAGAAGACGGCATACGAGATCAACCGTAGTGACTGGAGTTCAGACGTGTGCTCTT	ACGTCCAAAC	TACGGTTGAT
AATGATACGGCGACCACCGAGATCTACACGATCCACTACACTCTTTCCCTACACGACGCTCT	CAAGCAGAAGACGGCATACGAGATTGCGATAGGTGACTGGAGTTCAGACGTGTGCTCTT	GATCCACTAC	CTATCGCAAT
AATGATACGGCGACCACCGAGATCTACACAGCCTATCACACTCTTTCCCTACACGACGCTCT	CAAGCAGAAGACGGCATACGAGATTGGTTCGAGTGACTGGAGTTCAGACGTGTGCTCTT	AGCCTATCAC	TCGAACCAAT
AATGATACGGCGACCACCGAGATCTACACAGCTACCAACACTCTTTCCCTACACGACGCTCT	CAAGCAGAAGACGGCATACGAGATAAGCGTTCGTGACTGGAGTTCAGACGTGTGCTCTT	AGCTACCAAC	GAACGCTTAT
AATGATACGGCGACCACCGAGATCTACACAGATTGCGACACTCTTTCCCTACACGACGCTCT	CAAGCAGAAGACGGCATACGAGATCGATTCTGGTGACTGGAGTTCAGACGTGTGCTCTT	AGATTGCGAC	CAGAATCGAT
AATGATACGGCGACCACCGAGATCTACACCACACATCACACTCTTTCCCTACACGACGCTCT	CAAGCAGAAGACGGCATACGAGATGCAACCATGTGACTGGAGTTCAGACGTGTGCTCTT	CACACATCAC	ATGGTTGCAT
AATGATACGGCGACCACCGAGATCTACACGAGCAATCACACTCTTTCCCTACACGACGCTCT	CAAGCAGAAGACGGCATACGAGATAATCCAGCGTGACTGGAGTTCAGACGTGTGCTCTT	GAGCAATCAC	GCTGGATTAT
AATGATACGGCGACCACCGAGATCTACACATAGAGCGACACTCTTTCCCTACACGACGCTCT	CAAGCAGAAGACGGCATACGAGATAGTGCATCGTGACTGGAGTTCAGACGTGTGCTCTT	ATAGAGCGAC	GATGCACTAT
AATGATACGGCGACCACCGAGATCTACACGACCGATAACACTCTTTCCCTACACGACGCTCT	CAAGCAGAAGACGGCATACGAGATGCATTGGTGTGACTGGAGTTCAGACGTGTGCTCTT	GACCGATAAC	ACCAATGCAT
AATGATACGGCGACCACCGAGATCTACACCAGACGTTACACTCTTTCCCTACACGACGCTCT	CAAGCAGAAGACGGCATACGAGATCTTAGGACGTGACTGGAGTTCAGACGTGTGCTCTT	CAGACGTTAC	GTCCTAAGAT
AATGATACGGCGACCACCGAGATCTACACCTGAACGTACACTCTTTCCCTACACGACGCTCT	CAAGCAGAAGACGGCATACGAGATATAGTCGGGTGACTGGAGTTCAGACGTGTGCTCTT	CTGAACGTAC	CCGACTATAT
AATGATACGGCGACCACCGAGATCTACACTTGGACTGACACTCTTTCCCTACACGACGCTCT	CAAGCAGAAGACGGCATACGAGATGAGACCAAGTGACTGGAGTTCAGACGTGTGCTCTT	TTGGACTGAC	TTGGTCTCAT
AATGATACGGCGACCACCGAGATCTACACGTCTGCAAACACTCTTTCCCTACACGACGCTCT	CAAGCAGAAGACGGCATACGAGATAACAAGGCGTGACTGGAGTTCAGACGTGTGCTCTT	GTCTGCAAAC	GCCTTGTTAT
AATGATACGGCGACCACCGAGATCTACACCCACATTGACACTCTTTCCCTACACGACGCTCT	CAAGCAGAAGACGGCATACGAGATCCAGTATCGTGACTGGAGTTCAGACGTGTGCTCTT	CCACATTGAC	GATACTGGAT
AATGATACGGCGACCACCGAGATCTACACGATGGAGTACACTCTTTCCCTACACGACGCTCT	CAAGCAGAAGACGGCATACGAGATCCTCGAATGTGACTGGAGTTCAGACGTGTGCTCTT	GATGGAGTAC	ATTCGAGGAT
AATGATACGGCGACCACCGAGATCTACACAGGTCAACACACTCTTTCCCTACACGACGCTCT	CAAGCAGAAGACGGCATACGAGATCAACTGACGTGACTGGAGTTCAGACGTGTGCTCTT	AGGTCAACAC	GTCAGTTGAT
AATGATACGGCGACCACCGAGATCTACACTACACACGACACTCTTTCCCTACACGACGCTCT	CAAGCAGAAGACGGCATACGAGATTGCTCTACGTGACTGGAGTTCAGACGTGTGCTCTT	TACACACGAC	GTAGAGCAAT
AATGATACGGCGACCACCGAGATCTACACCAAGTCGTACACTCTTTCCCTACACGACGCTCT	CAAGCAGAAGACGGCATACGAGATCATCACGTGTGACTGGAGTTCAGACGTGTGCTCTT	CAAGTCGTAC	ACGTGATGAT
AATGATACGGCGACCACCGAGATCTACACAGCTAGTGACACTCTTTCCCTACACGACGCTCT	CAAGCAGAAGACGGCATACGAGATGCCACTTAGTGACTGGAGTTCAGACGTGTGCTCTT	AGCTAGTGAC	TAAGTGGCAT
AATGATACGGCGACCACCGAGATCTACACCTCCTAGTACACTCTTTCCCTACACGACGCTCT	CAAGCAGAAGACGGCATACGAGATGCTTCACAGTGACTGGAGTTCAGACGTGTGCTCTT	CTCCTAGTAC	TGTGAAGCAT
AATGATACGGCGACCACCGAGATCTACACACTCCTACACACTCTTTCCCTACACGACGCTCT	CAAGCAGAAGACGGCATACGAGATACCGAATGGTGACTGGAGTTCAGACGTGTGCTCTT	ACTCCTACAC	CATTCGGTAT
AATGATACGGCGACCACCGAGATCTACACCAATCAGGACACTCTTTCCCTACACGACGCTCT	CAAGCAGAAGACGGCATACGAGATCTCACCAAGTGACTGGAGTTCAGACGTGTGCTCTT	CAATCAGGAC	TTGGTGAGAT
AATGATACGGCGACCACCGAGATCTACACTCGTGCATACACTCTTTCCCTACACGACGCTCT	CAAGCAGAAGACGGCATACGAGATCAGAACTGGTGACTGGAGTTCAGACGTGTGCTCTT	TCGTGCATAC	CAGTTCTGAT
AATGATACGGCGACCACCGAGATCTACACTAACGTCGACACTCTTTCCCTACACGACGCTCT	CAAGCAGAAGACGGCATACGAGATAGAAGCCTGTGACTGGAGTTCAGACGTGTGCTCTT	TAACGTCGAC	AGGCTTCTAT
AATGATACGGCGACCACCGAGATCTACACAAGGCGTAACACTCTTTCCCTACACGACGCTCT	CAAGCAGAAGACGGCATACGAGATCACGATTCGTGACTGGAGTTCAGACGTGTGCTCTT	AAGGCGTAAC	GAATCGTGAT
AATGATACGGCGACCACCGAGATCTACACTCTTACGGACACTCTTTCCCTACACGACGCTCT	CAAGCAGAAGACGGCATACGAGATAAGCTGGTGTGACTGGAGTTCAGACGTGTGCTCTT	TCTTACGGAC	ACCAGCTTAT
AATGATACGGCGACCACCGAGATCTACACCGTGTGATACACTCTTTCCCTACACGACGCTCT	CAAGCAGAAGACGGCATACGAGATGCAATGAGGTGACTGGAGTTCAGACGTGTGCTCTT	CGTGTGATAC	CTCATTGCAT
AATGATACGGCGACCACCGAGATCTACACAACAGGTGACACTCTTTCCCTACACGACGCTCT	CAAGCAGAAGACGGCATACGAGATCTCTATCGGTGACTGGAGTTCAGACGTGTGCTCTT	AACAGGTGAC	CGATAGAGAT
AATGATACGGCGACCACCGAGATCTACACAGTCGAAGACACTCTTTCCCTACACGACGCTCT	CAAGCAGAAGACGGCATACGAGATACTCTCCAGTGACTGGAGTTCAGACGTGTGCTCTT	AGTCGAAGAC	TGGAGAGTAT
AATGATACGGCGACCACCGAGATCTACACTGGAAGCAACACTCTTTCCCTACACGACGCTCT	CAAGCAGAAGACGGCATACGAGATCAGCATACGTGACTGGAGTTCAGACGTGTGCTCTT	TGGAAGCAAC	GTATGCTGAT
AATGATACGGCGACCACCGAGATCTACACCTCGTTCTACACTCTTTCCCTACACGACGCTCT	CAAGCAGAAGACGGCATACGAGATTACTCCAGGTGACTGGAGTTCAGACGTGTGCTCTT	CTCGTTCTAC	CTGGAGTAAT
AATGATACGGCGACCACCGAGATCTACACACGAGAACACACTCTTTCCCTACACGACGCTCT	CAAGCAGAAGACGGCATACGAGATGAGGCATTGTGACTGGAGTTCAGACGTGTGCTCTT	ACGAGAACAC	AATGCCTCAT
AATGATACGGCGACCACCGAGATCTACACAAGCCTGAACACTCTTTCCCTACACGACGCTCT	CAAGCAGAAGACGGCATACGAGATACACCTCAGTGACTGGAGTTCAGACGTGTGCTCTT	AAGCCTGAAC	TGAGGTGTAT
AATGATACGGCGACCACCGAGATCTACACCTACAAGGACACTCTTTCCCTACACGACGCTCT	CAAGCAGAAGACGGCATACGAGATCGCAATGTGTGACTGGAGTTCAGACGTGTGCTCTT	CTACAAGGAC	ACATTGCGAT
AATGATACGGCGACCACCGAGATCTACACCGATGTTCACACTCTTTCCCTACACGACGCTCT	CAAGCAGAAGACGGCATACGAGATCCTAGAGAGTGACTGGAGTTCAGACGTGTGCTCTT	CGATGTTCAC	TCTCTAGGAT
AATGATACGGCGACCACCGAGATCTACACACCGGTTAACACTCTTTCCCTACACGACGCTCT	CAAGCAGAAGACGGCATACGAGATTACTAGCGGTGACTGGAGTTCAGACGTGTGCTCTT	ACCGGTTAAC	CGCTAGTAAT
AATGATACGGCGACCACCGAGATCTACACGAACGGTTACACTCTTTCCCTACACGACGCTCT	CAAGCAGAAGACGGCATACGAGATCGTCCATTGTGACTGGAGTTCAGACGTGTGCTCTT	GAACGGTTAC	AATGGACGAT
AATGATACGGCGACCACCGAGATCTACACCTGTACCAACACTCTTTCCCTACACGACGCTCT	CAAGCAGAAGACGGCATACGAGATTCGCTATCGTGACTGGAGTTCAGACGTGTGCTCTT	CTGTACCAAC	GATAGCGAAT

AATGATACGGCGACCACCGAGATCTACACGCGCATATACACTCTTTCCCTACACGACGCTCT	CAAGCAGAAGACGGCATACGAGATAATGGTCGGTGACTGGAGTTCAGACGTGTGCTCTT	GCGCATATAC	CGACCATTAT
AATGATACGGCGACCACCGAGATCTACACTGATAGGCACACTCTTTCCCTACACGACGCTCT	CAAGCAGAAGACGGCATACGAGATGAACGAAGGTGACTGGAGTTCAGACGTGTGCTCTT	TGATAGGCAC	CTTCGTTCAT
AATGATACGGCGACCACCGAGATCTACACCATCCAAGACACTCTTTCCCTACACGACGCTCT	CAAGCAGAAGACGGCATACGAGATACCTAGACGTGACTGGAGTTCAGACGTGTGCTCTT	CATCCAAGAC	GTCTAGGTAT
AATGATACGGCGACCACCGAGATCTACACGTGAGACTACACTCTTTCCCTACACGACGCTCT	CAAGCAGAAGACGGCATACGAGATTACGACGTGTGACTGGAGTTCAGACGTGTGCTCTT	GTGAGACTAC	ACGTCGTAAT
AATGATACGGCGACCACCGAGATCTACACCTGATGAGACACTCTTTCCCTACACGACGCTCT	CAAGCAGAAGACGGCATACGAGATTTGAGCTCGTGACTGGAGTTCAGACGTGTGCTCTT	CTGATGAGAC	GAGCTCAAAT
AATGATACGGCGACCACCGAGATCTACACACGGTACAACACTCTTTCCCTACACGACGCTCT	CAAGCAGAAGACGGCATACGAGATAGTACACGGTGACTGGAGTTCAGACGTGTGCTCTT	ACGGTACAAC	CGTGTACTAT
AATGATACGGCGACCACCGAGATCTACACCTCGACTTACACTCTTTCCCTACACGACGCTCT	CAAGCAGAAGACGGCATACGAGATTGTCAGTGGTGACTGGAGTTCAGACGTGTGCTCTT	CTCGACTTAC	CACTGACAAT
AATGATACGGCGACCACCGAGATCTACACACAACGTGACACTCTTTCCCTACACGACGCTCT	CAAGCAGAAGACGGCATACGAGATGACTACGAGTGACTGGAGTTCAGACGTGTGCTCTT	ACAACGTGAC	TCGTAGTCAT
AATGATACGGCGACCACCGAGATCTACACTGCTGTGAACACTCTTTCCCTACACGACGCTCT	CAAGCAGAAGACGGCATACGAGATTTACGTGCGTGACTGGAGTTCAGACGTGTGCTCTT	TGCTGTGAAC	GCACGTAAAT
AATGATACGGCGACCACCGAGATCTACACCCAAGTAGACACTCTTTCCCTACACGACGCTCT	CAAGCAGAAGACGGCATACGAGATACTGCTTGGTGACTGGAGTTCAGACGTGTGCTCTT	CCAAGTAGAC	CAAGCAGTAT
AATGATACGGCGACCACCGAGATCTACACAACTGAGGACACTCTTTCCCTACACGACGCTCT	CAAGCAGAAGACGGCATACGAGATGCCTATGTGTGACTGGAGTTCAGACGTGTGCTCTT	AACTGAGGAC	ACATAGGCAT
AATGATACGGCGACCACCGAGATCTACACAGGTAGGAACACTCTTTCCCTACACGACGCTCT	CAAGCAGAAGACGGCATACGAGATGTACCACAGTGACTGGAGTTCAGACGTGTGCTCTT	AGGTAGGAAC	TGTGGTACAT
AATGATACGGCGACCACCGAGATCTACACTTCGCCATACACTCTTTCCCTACACGACGCTCT	CAAGCAGAAGACGGCATACGAGATTAGTGGTGGTGACTGGAGTTCAGACGTGTGCTCTT	TTCGCCATAC	CACCACTAAT
AATGATACGGCGACCACCGAGATCTACACCAGGTAAGACACTCTTTCCCTACACGACGCTCT	CAAGCAGAAGACGGCATACGAGATATACGCAGGTGACTGGAGTTCAGACGTGTGCTCTT	CAGGTAAGAC	CTGCGTATAT
AATGATACGGCGACCACCGAGATCTACACGTATCGAGACACTCTTTCCCTACACGACGCTCT	CAAGCAGAAGACGGCATACGAGATAAGACCGTGTGACTGGAGTTCAGACGTGTGCTCTT	GTATCGAGAC	ACGGTCTTAT
AATGATACGGCGACCACCGAGATCTACACTTCACGGAACACTCTTTCCCTACACGACGCTCT	CAAGCAGAAGACGGCATACGAGATCTCCAATCGTGACTGGAGTTCAGACGTGTGCTCTT	TTCACGGAAC	GATTGGAGAT
AATGATACGGCGACCACCGAGATCTACACGAGCTCTAACACTCTTTCCCTACACGACGCTCT	CAAGCAGAAGACGGCATACGAGATTCTGGACAGTGACTGGAGTTCAGACGTGTGCTCTT	GAGCTCTAAC	TGTCCAGAAT
AATGATACGGCGACCACCGAGATCTACACGTCAGTCAACACTCTTTCCCTACACGACGCTCT	CAAGCAGAAGACGGCATACGAGATAACACTGGGTGACTGGAGTTCAGACGTGTGCTCTT	GTCAGTCAAC	CCAGTGTTAT
AATGATACGGCGACCACCGAGATCTACACCACGTCTAACACTCTTTCCCTACACGACGCTCT	CAAGCAGAAGACGGCATACGAGATTTGGTGCAGTGACTGGAGTTCAGACGTGTGCTCTT	CACGTCTAAC	TGCACCAAAT
AATGATACGGCGACCACCGAGATCTACACAATTCCGGACACTCTTTCCCTACACGACGCTCT	CAAGCAGAAGACGGCATACGAGATCCTGTCAAGTGACTGGAGTTCAGACGTGTGCTCTT	AATTCCGGAC	TTGACAGGAT
AATGATACGGCGACCACCGAGATCTACACTCTAGGAGACACTCTTTCCCTACACGACGCTCT	CAAGCAGAAGACGGCATACGAGATCTATGCCTGTGACTGGAGTTCAGACGTGTGCTCTT	TCTAGGAGAC	AGGCATAGAT
AATGATACGGCGACCACCGAGATCTACACATCCGTTGACACTCTTTCCCTACACGACGCTCT	CAAGCAGAAGACGGCATACGAGATTTCGGCTAGTGACTGGAGTTCAGACGTGTGCTCTT	ATCCGTTGAC	TAGCCGAAAT
AATGATACGGCGACCACCGAGATCTACACGATAGCCAACACTCTTTCCCTACACGACGCTCT	CAAGCAGAAGACGGCATACGAGATACCGACAAGTGACTGGAGTTCAGACGTGTGCTCTT	GATAGCCAAC	TTGTCGGTAT
AATGATACGGCGACCACCGAGATCTACACTATGACCGACACTCTTTCCCTACACGACGCTCT	CAAGCAGAAGACGGCATACGAGATCGTAGATGGTGACTGGAGTTCAGACGTGTGCTCTT	TATGACCGAC	CATCTACGAT
AATGATACGGCGACCACCGAGATCTACACCGATTGGAACACTCTTTCCCTACACGACGCTCT	CAAGCAGAAGACGGCATACGAGATCTGTATGCGTGACTGGAGTTCAGACGTGTGCTCTT	CGATTGGAAC	GCATACAGAT
AATGATACGGCGACCACCGAGATCTACACACAAGCTCACACTCTTTCCCTACACGACGCTCT	CAAGCAGAAGACGGCATACGAGATGTTGCTGTGTGACTGGAGTTCAGACGTGTGCTCTT	ACAAGCTCAC	ACAGCAACAT
AATGATACGGCGACCACCGAGATCTACACGAACCTTCACACTCTTTCCCTACACGACGCTCT	CAAGCAGAAGACGGCATACGAGATAGAACCAGGTGACTGGAGTTCAGACGTGTGCTCTT	GAACCTTCAC	CTGGTTCTAT
AATGATACGGCGACCACCGAGATCTACACAGCGAGATACACTCTTTCCCTACACGACGCTCT	CAAGCAGAAGACGGCATACGAGATGATGTCGAGTGACTGGAGTTCAGACGTGTGCTCTT	AGCGAGATAC	TCGACATCAT
AATGATACGGCGACCACCGAGATCTACACCCGTAACTACACTCTTTCCCTACACGACGCTCT	CAAGCAGAAGACGGCATACGAGATAGGAGGTTGTGACTGGAGTTCAGACGTGTGCTCTT	CCGTAACTAC	AACCTCCTAT
AATGATACGGCGACCACCGAGATCTACACTCAGACACACACTCTTTCCCTACACGACGCTCT	CAAGCAGAAGACGGCATACGAGATAATCGCTGGTGACTGGAGTTCAGACGTGTGCTCTT	TCAGACACAC	CAGCGATTAT
AATGATACGGCGACCACCGAGATCTACACCGAAGTCAACACTCTTTCCCTACACGACGCTCT	CAAGCAGAAGACGGCATACGAGATAGTGACCTGTGACTGGAGTTCAGACGTGTGCTCTT	CGAAGTCAAC	AGGTCACTAT
AATGATACGGCGACCACCGAGATCTACACGTGATCCAACACTCTTTCCCTACACGACGCTCT	CAAGCAGAAGACGGCATACGAGATCGAATTGCGTGACTGGAGTTCAGACGTGTGCTCTT	GTGATCCAAC	GCAATTCGAT
AATGATACGGCGACCACCGAGATCTACACACTGGTGTACACTCTTTCCCTACACGACGCTCT	CAAGCAGAAGACGGCATACGAGATCAAGAAGCGTGACTGGAGTTCAGACGTGTGCTCTT	ACTGGTGTAC	GCTTCTTGAT
AATGATACGGCGACCACCGAGATCTACACCTAACCTGACACTCTTTCCCTACACGACGCTCT	CAAGCAGAAGACGGCATACGAGATCACCAGTTGTGACTGGAGTTCAGACGTGTGCTCTT	CTAACCTGAC	AACTGGTGAT
AATGATACGGCGACCACCGAGATCTACACAGCCAACTACACTCTTTCCCTACACGACGCTCT	CAAGCAGAAGACGGCATACGAGATGTATTCCGGTGACTGGAGTTCAGACGTGTGCTCTT	AGCCAACTAC	CGGAATACAT
AATGATACGGCGACCACCGAGATCTACACCCAGTTGAACACTCTTTCCCTACACGACGCTCT	CAAGCAGAAGACGGCATACGAGATTTCGAAGCGTGACTGGAGTTCAGACGTGTGCTCTT	CCAGTTGAAC	GCTTCGAAAT
AATGATACGGCGACCACCGAGATCTACACAAGTGCAGACACTCTTTCCCTACACGACGCTCT	CAAGCAGAAGACGGCATACGAGATAGACCTTGGTGACTGGAGTTCAGACGTGTGCTCTT	AAGTGCAGAC	CAAGGTCTAT
AATGATACGGCGACCACCGAGATCTACACAACCGTGTACACTCTTTCCCTACACGACGCTCT	CAAGCAGAAGACGGCATACGAGATCCAAGGTTGTGACTGGAGTTCAGACGTGTGCTCTT	AACCGTGTAC	AACCTTGGAT
AATGATACGGCGACCACCGAGATCTACACCGCGTATTACACTCTTTCCCTACACGACGCTCT	CAAGCAGAAGACGGCATACGAGATACGTATGGGTGACTGGAGTTCAGACGTGTGCTCTT	CGCGTATTAC	CCATACGTAT
AATGATACGGCGACCACCGAGATCTACACAGTTCGCAACACTCTTTCCCTACACGACGCTCT	CAAGCAGAAGACGGCATACGAGATAAGGACCAGTGACTGGAGTTCAGACGTGTGCTCTT	AGTTCGCAAC	TGGTCCTTAT
AATGATACGGCGACCACCGAGATCTACACTAGTCAGCACACTCTTTCCCTACACGACGCTCT	CAAGCAGAAGACGGCATACGAGATTATGCGGTGTGACTGGAGTTCAGACGTGTGCTCTT	TAGTCAGCAC	ACCGCATAAT
AATGATACGGCGACCACCGAGATCTACACAACACCACACACTCTTTCCCTACACGACGCTCT	CAAGCAGAAGACGGCATACGAGATAAGGAAGGGTGACTGGAGTTCAGACGTGTGCTCTT	AACACCACAC	CCTTCCTTAT
AATGATACGGCGACCACCGAGATCTACACGTAAGCACACACTCTTTCCCTACACGACGCTCT	CAAGCAGAAGACGGCATACGAGATAGCGTGTAGTGACTGGAGTTCAGACGTGTGCTCTT	GTAAGCACAC	TACACGCTAT
AATGATACGGCGACCACCGAGATCTACACGTCCTTGAACACTCTTTCCCTACACGACGCTCT	CAAGCAGAAGACGGCATACGAGATTCTACGCAGTGACTGGAGTTCAGACGTGTGCTCTT	GTCCTTGAAC	TGCGTAGAAT
AATGATACGGCGACCACCGAGATCTACACCAGGTTCAACACTCTTTCCCTACACGACGCTCT	CAAGCAGAAGACGGCATACGAGATTGGCTCTTGTGACTGGAGTTCAGACGTGTGCTCTT	CAGGTTCAAC	AAGAGCCAAT
AATGATACGGCGACCACCGAGATCTACACCCAACACTACACTCTTTCCCTACACGACGCTCT	CAAGCAGAAGACGGCATACGAGATCCTTCCATGTGACTGGAGTTCAGACGTGTGCTCTT	CCAACACTAC	ATGGAAGGAT
AATGATACGGCGACCACCGAGATCTACACGAGAGTACACACTCTTTCCCTACACGACGCTCT	CAAGCAGAAGACGGCATACGAGATATACTGGCGTGACTGGAGTTCAGACGTGTGCTCTT	GAGAGTACAC	GCCAGTATAT
AATGATACGGCGACCACCGAGATCTACACAGATACGGACACTCTTTCCCTACACGACGCTCT	CAAGCAGAAGACGGCATACGAGATAACCTACGGTGACTGGAGTTCAGACGTGTGCTCTT	AGATACGGAC	CGTAGGTTAT
AATGATACGGCGACCACCGAGATCTACACGTTCTTCGACACTCTTTCCCTACACGACGCTCT	CAAGCAGAAGACGGCATACGAGATCATACTCGGTGACTGGAGTTCAGACGTGTGCTCTT	GTTCTTCGAC	CGAGTATGAT
AATGATACGGCGACCACCGAGATCTACACATTCCGCTACACTCTTTCCCTACACGACGCTCT	CAAGCAGAAGACGGCATACGAGATTGCACTTGGTGACTGGAGTTCAGACGTGTGCTCTT	ATTCCGCTAC	CAAGTGCAAT
AATGATACGGCGACCACCGAGATCTACACAAGCTCACACACTCTTTCCCTACACGACGCTCT	CAAGCAGAAGACGGCATACGAGATTCACTCGAGTGACTGGAGTTCAGACGTGTGCTCTT	AAGCTCACAC	TCGAGTGAAT
AATGATACGGCGACCACCGAGATCTACACTGATCACGACACTCTTTCCCTACACGACGCTCT	CAAGCAGAAGACGGCATACGAGATCACTGTAGGTGACTGGAGTTCAGACGTGTGCTCTT	TGATCACGAC	CTACAGTGAT
AATGATACGGCGACCACCGAGATCTACACCAATGCGAACACTCTTTCCCTACACGACGCTCT	CAAGCAGAAGACGGCATACGAGATGTACGATCGTGACTGGAGTTCAGACGTGTGCTCTT	CAATGCGAAC	GATCGTACAT
AATGATACGGCGACCACCGAGATCTACACATGCGTCAACACTCTTTCCCTACACGACGCTCT	CAAGCAGAAGACGGCATACGAGATTGGTGAAGGTGACTGGAGTTCAGACGTGTGCTCTT	ATGCGTCAAC	CTTCACCAAT
AATGATACGGCGACCACCGAGATCTACACTACATCGGACACTCTTTCCCTACACGACGCTCT	CAAGCAGAAGACGGCATACGAGATTAGCTGAGGTGACTGGAGTTCAGACGTGTGCTCTT	TACATCGGAC	CTCAGCTAAT
AATGATACGGCGACCACCGAGATCTACACACTGCGAAACACTCTTTCCCTACACGACGCTCT	CAAGCAGAAGACGGCATACGAGATAGAGCAGAGTGACTGGAGTTCAGACGTGTGCTCTT	ACTGCGAAAC	TCTGCTCTAT
AATGATACGGCGACCACCGAGATCTACACTCTGTCGTACACTCTTTCCCTACACGACGCTCT	CAAGCAGAAGACGGCATACGAGATCTTCGGTTGTGACTGGAGTTCAGACGTGTGCTCTT	TCTGTCGTAC	AACCGAAGAT
AATGATACGGCGACCACCGAGATCTACACCTCAAGCTACACTCTTTCCCTACACGACGCTCT	CAAGCAGAAGACGGCATACGAGATACAACAGCGTGACTGGAGTTCAGACGTGTGCTCTT	CTCAAGCTAC	GCTGTTGTAT
AATGATACGGCGACCACCGAGATCTACACAACCACTCACACTCTTTCCCTACACGACGCTCT	CAAGCAGAAGACGGCATACGAGATAGCCGTAAGTGACTGGAGTTCAGACGTGTGCTCTT	AACCACTCAC	TTACGGCTAT
AATGATACGGCGACCACCGAGATCTACACCTTACAGCACACTCTTTCCCTACACGACGCTCT	CAAGCAGAAGACGGCATACGAGATCTCTTGTCGTGACTGGAGTTCAGACGTGTGCTCTT	CTTACAGCAC	GACAAGAGAT
AATGATACGGCGACCACCGAGATCTACACAGTCTTGGACACTCTTTCCCTACACGACGCTCT	CAAGCAGAAGACGGCATACGAGATCAGATCCTGTGACTGGAGTTCAGACGTGTGCTCTT	AGTCTTGGAC	AGGATCTGAT
AATGATACGGCGACCACCGAGATCTACACCACGCAATACACTCTTTCCCTACACGACGCTCT	CAAGCAGAAGACGGCATACGAGATGATGCTACGTGACTGGAGTTCAGACGTGTGCTCTT	CACGCAATAC	GTAGCATCAT
AATGATACGGCGACCACCGAGATCTACACAGCTTCAGACACTCTTTCCCTACACGACGCTCT	CAAGCAGAAGACGGCATACGAGATAGGAACACGTGACTGGAGTTCAGACGTGTGCTCTT	AGCTTCAGAC	GTGTTCCTAT
AATGATACGGCGACCACCGAGATCTACACCCTCGTTAACACTCTTTCCCTACACGACGCTCT	CAAGCAGAAGACGGCATACGAGATACCATCCTGTGACTGGAGTTCAGACGTGTGCTCTT	CCTCGTTAAC	AGGATGGTAT
AATGATACGGCGACCACCGAGATCTACACTGAGACGAACACTCTTTCCCTACACGACGCTCT	CAAGCAGAAGACGGCATACGAGATGAACGTGAGTGACTGGAGTTCAGACGTGTGCTCTT	TGAGACGAAC	TCACGTTCAT
AATGATACGGCGACCACCGAGATCTACACCACAGGAAACACTCTTTCCCTACACGACGCTCT	CAAGCAGAAGACGGCATACGAGATTAGAACGCGTGACTGGAGTTCAGACGTGTGCTCTT	CACAGGAAAC	GCGTTCTAAT
AATGATACGGCGACCACCGAGATCTACACACTCAACGACACTCTTTCCCTACACGACGCTCT	CAAGCAGAAGACGGCATACGAGATAACCAGAGGTGACTGGAGTTCAGACGTGTGCTCTT	ACTCAACGAC	CTCTGGTTAT
AATGATACGGCGACCACCGAGATCTACACAAGCGACTACACTCTTTCCCTACACGACGCTCT	CAAGCAGAAGACGGCATACGAGATCGACCTAAGTGACTGGAGTTCAGACGTGTGCTCTT	AAGCGACTAC	TTAGGTCGAT
AATGATACGGCGACCACCGAGATCTACACCCTACCTAACACTCTTTCCCTACACGACGCTCT	CAAGCAGAAGACGGCATACGAGATCTCTCAGAGTGACTGGAGTTCAGACGTGTGCTCTT	CCTACCTAAC	TCTGAGAGAT
AATGATACGGCGACCACCGAGATCTACACATCTCCTGACACTCTTTCCCTACACGACGCTCT	CAAGCAGAAGACGGCATACGAGATAGGCTGAAGTGACTGGAGTTCAGACGTGTGCTCTT	ATCTCCTGAC	TTCAGCCTAT
AATGATACGGCGACCACCGAGATCTACACTCACGATGACACTCTTTCCCTACACGACGCTCT	CAAGCAGAAGACGGCATACGAGATATCGGAGAGTGACTGGAGTTCAGACGTGTGCTCTT	TCACGATGAC	TCTCCGATAT
AATGATACGGCGACCACCGAGATCTACACCCACAACAACACTCTTTCCCTACACGACGCTCT	CAAGCAGAAGACGGCATACGAGATGATACCTGGTGACTGGAGTTCAGACGTGTGCTCTT	CCACAACAAC	CAGGTATCAT
AATGATACGGCGACCACCGAGATCTACACAGGTCTGTACACTCTTTCCCTACACGACGCTCT	CAAGCAGAAGACGGCATACGAGATTCCTGACTGTGACTGGAGTTCAGACGTGTGCTCTT	AGGTCTGTAC	AGTCAGGAAT
AATGATACGGCGACCACCGAGATCTACACAGAAGGACACACTCTTTCCCTACACGACGCTCT	CAAGCAGAAGACGGCATACGAGATTCAGCCTTGTGACTGGAGTTCAGACGTGTGCTCTT	AGAAGGACAC	AAGGCTGAAT
AATGATACGGCGACCACCGAGATCTACACGCGTATCAACACTCTTTCCCTACACGACGCTCT	CAAGCAGAAGACGGCATACGAGATAAGCATCGGTGACTGGAGTTCAGACGTGTGCTCTT	GCGTATCAAC	CGATGCTTAT
AATGATACGGCGACCACCGAGATCTACACCAACACAGACACTCTTTCCCTACACGACGCTCT	CAAGCAGAAGACGGCATACGAGATGCCAATACGTGACTGGAGTTCAGACGTGTGCTCTT	CAACACAGAC	GTATTGGCAT
AATGATACGGCGACCACCGAGATCTACACTCCACGTTACACTCTTTCCCTACACGACGCTCT	CAAGCAGAAGACGGCATACGAGATGACACAGTGTGACTGGAGTTCAGACGTGTGCTCTT	TCCACGTTAC	ACTGTGTCAT
AATGATACGGCGACCACCGAGATCTACACATCGCAACACACTCTTTCCCTACACGACGCTCT	CAAGCAGAAGACGGCATACGAGATAAGAGGCAGTGACTGGAGTTCAGACGTGTGCTCTT	ATCGCAACAC	TGCCTCTTAT
AATGATACGGCGACCACCGAGATCTACACACGTCGTTACACTCTTTCCCTACACGACGCTCT	CAAGCAGAAGACGGCATACGAGATGAAGACTGGTGACTGGAGTTCAGACGTGTGCTCTT	ACGTCGTTAC	CAGTCTTCAT
AATGATACGGCGACCACCGAGATCTACACCGAATACGACACTCTTTCCCTACACGACGCTCT	CAAGCAGAAGACGGCATACGAGATCCGTTATGGTGACTGGAGTTCAGACGTGTGCTCTT	CGAATACGAC	CATAACGGAT
AATGATACGGCGACCACCGAGATCTACACTGCTTGCTACACTCTTTCCCTACACGACGCTCT	CAAGCAGAAGACGGCATACGAGATCTAGCAGTGTGACTGGAGTTCAGACGTGTGCTCTT	TGCTTGCTAC	ACTGCTAGAT
AATGATACGGCGACCACCGAGATCTACACCTCGAACAACACTCTTTCCCTACACGACGCTCT	CAAGCAGAAGACGGCATACGAGATGCCAGAATGTGACTGGAGTTCAGACGTGTGCTCTT	CTCGAACAAC	ATTCTGGCAT
AATGATACGGCGACCACCGAGATCTACACACATGGAGACACTCTTTCCCTACACGACGCTCT	CAAGCAGAAGACGGCATACGAGATCGAGAGAAGTGACTGGAGTTCAGACGTGTGCTCTT	ACATGGAGAC	TTCTCTCGAT
AATGATACGGCGACCACCGAGATCTACACACAAGACGACACTCTTTCCCTACACGACGCTCT	CAAGCAGAAGACGGCATACGAGATAACTCGGAGTGACTGGAGTTCAGACGTGTGCTCTT	ACAAGACGAC	TCCGAGTTAT
AATGATACGGCGACCACCGAGATCTACACCGCCTTATACACTCTTTCCCTACACGACGCTCT	CAAGCAGAAGACGGCATACGAGATACAGTTCGGTGACTGGAGTTCAGACGTGTGCTCTT	CGCCTTATAC	CGAACTGTAT
AATGATACGGCGACCACCGAGATCTACACAGCAGACAACACTCTTTCCCTACACGACGCTCT	CAAGCAGAAGACGGCATACGAGATTGACCGTTGTGACTGGAGTTCAGACGTGTGCTCTT	AGCAGACAAC	AACGGTCAAT
AATGATACGGCGACCACCGAGATCTACACGTTAAGCGACACTCTTTCCCTACACGACGCTCT	CAAGCAGAAGACGGCATACGAGATCATCTGCTGTGACTGGAGTTCAGACGTGTGCTCTT	GTTAAGCGAC	AGCAGATGAT
AATGATACGGCGACCACCGAGATCTACACCATGGATCACACTCTTTCCCTACACGACGCTCT	CAAGCAGAAGACGGCATACGAGATCGCTGATAGTGACTGGAGTTCAGACGTGTGCTCTT	CATGGATCAC	TATCAGCGAT
AATGATACGGCGACCACCGAGATCTACACACAGAGGTACACTCTTTCCCTACACGACGCTCT	CAAGCAGAAGACGGCATACGAGATTCGTCTGAGTGACTGGAGTTCAGACGTGTGCTCTT	ACAGAGGTAC	TCAGACGAAT
AATGATACGGCGACCACCGAGATCTACACTAAGTGGCACACTCTTTCCCTACACGACGCTCT	CAAGCAGAAGACGGCATACGAGATCACATGGTGTGACTGGAGTTCAGACGTGTGCTCTT	TAAGTGGCAC	ACCATGTGAT
AATGATACGGCGACCACCGAGATCTACACAGTCAGGTACACTCTTTCCCTACACGACGCTCT	CAAGCAGAAGACGGCATACGAGATCGAGTTAGGTGACTGGAGTTCAGACGTGTGCTCTT	AGTCAGGTAC	CTAACTCGAT
AATGATACGGCGACCACCGAGATCTACACGCCTTAACACACTCTTTCCCTACACGACGCTCT	CAAGCAGAAGACGGCATACGAGATAGCTAAGCGTGACTGGAGTTCAGACGTGTGCTCTT	GCCTTAACAC	GCTTAGCTAT
AATGATACGGCGACCACCGAGATCTACACGTTGGCATACACTCTTTCCCTACACGACGCTCT	CAAGCAGAAGACGGCATACGAGATGTTCCATGGTGACTGGAGTTCAGACGTGTGCTCTT	GTTGGCATAC	CATGGAACAT
AATGATACGGCGACCACCGAGATCTACACCAACCTCTACACTCTTTCCCTACACGACGCTCT	CAAGCAGAAGACGGCATACGAGATGCATCCTAGTGACTGGAGTTCAGACGTGTGCTCTT	CAACCTCTAC	TAGGATGCAT
AATGATACGGCGACCACCGAGATCTACACTGGATGGTACACTCTTTCCCTACACGACGCTCT	CAAGCAGAAGACGGCATACGAGATCCATGAACGTGACTGGAGTTCAGACGTGTGCTCTT	TGGATGGTAC	GTTCATGGAT
AATGATACGGCGACCACCGAGATCTACACCTATCCACACACTCTTTCCCTACACGACGCTCT	CAAGCAGAAGACGGCATACGAGATATCCACGAGTGACTGGAGTTCAGACGTGTGCTCTT	CTATCCACAC	TCGTGGATAT
AATGATACGGCGACCACCGAGATCTACACGATCTCAGACACTCTTTCCCTACACGACGCTCT	CAAGCAGAAGACGGCATACGAGATGAGAAGGTGTGACTGGAGTTCAGACGTGTGCTCTT	GATCTCAGAC	ACCTTCTCAT
AATGATACGGCGACCACCGAGATCTACACGAACGAAGACACTCTTTCCCTACACGACGCTCT	CAAGCAGAAGACGGCATACGAGATTGATAGGCGTGACTGGAGTTCAGACGTGTGCTCTT	GAACGAAGAC	GCCTATCAAT

AATGATACGGCGACCACCGAGATCTACACCTAGACACACTCTTTCCCTACACGACGCTCT	CAAGCAGAAGACGGCATACGAGATCATCCAAGGTGACTGGAGTTCAGACGTGTGCTCTT	ACCTAGACAC	CTTGGATGAT
AATGATACGGCGACCACCGAGATCTACTACGACGTACACTCTTTCCCTACACGACGCTCT	CAAGCAGAAGACGGCATACGAGATGTGAGACTGTGACTGGAGTTCAGACGTGTGCTCTT	TACGACGTAC	AGTCTCACAT
AATGATACGGCGACCACCGAGATCTACTTGAGCTCACACTCTTTCCCTACACGACGCTCT	CAAGCAGAAGACGGCATACGAGATCTGATGAGGTGACTGGAGTTCAGACGTGTGCTCTT	TTGAGCTCAC	CTCATCAGAT
AATGATACGGCGACCACCGAGATCTACAGTACACGACACTCTTTCCCTACACGACGCTCT	CAAGCAGAAGACGGCATACGAGATACGGTACAGTGACTGGAGTTCAGACGTGTGCTCTT	AGTACACGAC	TGTACCGTAT
AATGATACGGCGACCACCGAGATCTACTGTCAGTGACACTCTTTCCCTACACGACGCTCT	CAAGCAGAAGACGGCATACGAGATCTCGACTTGTGACTGGAGTTCAGACGTGTGCTCTT	TGTCAGTGAC	AAGTCGAGAT
AATGATACGGCGACCACCGAGATCTACGACTACGAACACTCTTTCCCTACACGACGCTCT	CAAGCAGAAGACGGCATACGAGATACAACGTGGTGACTGGAGTTCAGACGTGTGCTCTT	GACTACGAAC	CACGTTGTAT
AATGATACGGCGACCACCGAGATCTACTTACGTGCACACTCTTTCCCTACACGACGCTCT	CAAGCAGAAGACGGCATACGAGATTGCTGTGAGTGACTGGAGTTCAGACGTGTGCTCTT	TTACGTGCAC	TCACAGCAAT
AATGATACGGCGACCACCGAGATCTACACTGCTTGACACTCTTTCCCTACACGACGCTCT	CAAGCAGAAGACGGCATACGAGATCCAAGTAGGTGACTGGAGTTCAGACGTGTGCTCTT	ACTGCTTGAC	CTACTTGGAT
AATGATACGGCGACCACCGAGATCTACGCCTATGTACACTCTTTCCCTACACGACGCTCT	CAAGCAGAAGACGGCATACGAGATAACTGAGGGTGACTGGAGTTCAGACGTGTGCTCTT	GCCTATGTAC	CCTCAGTTAT
AATGATACGGCGACCACCGAGATCTACGTACCACAACACTCTTTCCCTACACGACGCTCT	CAAGCAGAAGACGGCATACGAGATAGGTAGGAGTGACTGGAGTTCAGACGTGTGCTCTT	GTACCACAAC	TCCTACCTAT
AATGATACGGCGACCACCGAGATCTACTAGTGGTGACACTCTTTCCCTACACGACGCTCT	CAAGCAGAAGACGGCATACGAGATTTCGCCATGTGACTGGAGTTCAGACGTGTGCTCTT	TAGTGGTGAC	ATGGCGAAAT
AATGATACGGCGACCACCGAGATCTACATACGCAGACACTCTTTCCCTACACGACGCTCT	CAAGCAGAAGACGGCATACGAGATCAGGTAAGGTGACTGGAGTTCAGACGTGTGCTCTT	ATACGCAGAC	CTTACCTGAT
AATGATACGGCGACCACCGAGATCTACAAGACCGTACACTCTTTCCCTACACGACGCTCT	CAAGCAGAAGACGGCATACGAGATGTATCGAGGTGACTGGAGTTCAGACGTGTGCTCTT	AAGACCGTAC	CTCGATACAT
AATGATACGGCGACCACCGAGATCTACCTCCAATCACACTCTTTCCCTACACGACGCTCT	CAAGCAGAAGACGGCATACGAGATTTCACGGAGTGACTGGAGTTCAGACGTGTGCTCTT	CTCCAATCAC	TCCGTGAAAT
AATGATACGGCGACCACCGAGATCTACTCTGGACAACACTCTTTCCCTACACGACGCTCT	CAAGCAGAAGACGGCATACGAGATGAGCTCTAGTGACTGGAGTTCAGACGTGTGCTCTT	TCTGGACAAC	TAGAGCTCAT
AATGATACGGCGACCACCGAGATCTACAACACTGGACACTCTTTCCCTACACGACGCTCT	CAAGCAGAAGACGGCATACGAGATGTCAGTCAGTGACTGGAGTTCAGACGTGTGCTCTT	AACACTGGAC	TGACTGACAT
AATGATACGGCGACCACCGAGATCTACTTGGTGCAACACTCTTTCCCTACACGACGCTCT	CAAGCAGAAGACGGCATACGAGATCACGTCTAGTGACTGGAGTTCAGACGTGTGCTCTT	TTGGTGCAAC	TAGACGTGAT
AATGATACGGCGACCACCGAGATCTACCCTGTCAAACACTCTTTCCCTACACGACGCTCT	CAAGCAGAAGACGGCATACGAGATAATTCCGGGTGACTGGAGTTCAGACGTGTGCTCTT	CCTGTCAAAC	CCGGAATTAT
AATGATACGGCGACCACCGAGATCTACCTATGCCTACACTCTTTCCCTACACGACGCTCT	CAAGCAGAAGACGGCATACGAGATTCTAGGAGGTGACTGGAGTTCAGACGTGTGCTCTT	CTATGCCTAC	CTCCTAGAAT
AATGATACGGCGACCACCGAGATCTACTTCGGCTAACACTCTTTCCCTACACGACGCTCT	CAAGCAGAAGACGGCATACGAGATATCCGTTGGTGACTGGAGTTCAGACGTGTGCTCTT	TTCGGCTAAC	CAACGGATAT
AATGATACGGCGACCACCGAGATCTACACCGACAAACACTCTTTCCCTACACGACGCTCT	CAAGCAGAAGACGGCATACGAGATGATAGCCAGTGACTGGAGTTCAGACGTGTGCTCTT	ACCGACAAAC	TGGCTATCAT
AATGATACGGCGACCACCGAGATCTACCGTAGATGACACTCTTTCCCTACACGACGCTCT	CAAGCAGAAGACGGCATACGAGATTATGACCGGTGACTGGAGTTCAGACGTGTGCTCTT	CGTAGATGAC	CGGTCATAAT
AATGATACGGCGACCACCGAGATCTACCTGTATGCACACTCTTTCCCTACACGACGCTCT	CAAGCAGAAGACGGCATACGAGATCGATTGGAGTGACTGGAGTTCAGACGTGTGCTCTT	CTGTATGCAC	TCCAATCGAT
AATGATACGGCGACCACCGAGATCTACGTTGCTGTACACTCTTTCCCTACACGACGCTCT	CAAGCAGAAGACGGCATACGAGATACAAGCTCGTGACTGGAGTTCAGACGTGTGCTCTT	GTTGCTGTAC	GAGCTTGTAT
AATGATACGGCGACCACCGAGATCTACAGAACCAGACACTCTTTCCCTACACGACGCTCT	CAAGCAGAAGACGGCATACGAGATGAACCTTCGTGACTGGAGTTCAGACGTGTGCTCTT	AGAACCAGAC	GAAGGTTCAT
AATGATACGGCGACCACCGAGATCTACGATGTCGAACACTCTTTCCCTACACGACGCTCT	CAAGCAGAAGACGGCATACGAGATAGCGAGATGTGACTGGAGTTCAGACGTGTGCTCTT	GATGTCGAAC	ATCTCGCTAT
AATGATACGGCGACCACCGAGATCTACAGGAGGTTACACTCTTTCCCTACACGACGCTCT	CAAGCAGAAGACGGCATACGAGATCCGTAACTGTGACTGGAGTTCAGACGTGTGCTCTT	AGGAGGTTAC	AGTTACGGAT
AATGATACGGCGACCACCGAGATCTACAATCGCTGACACTCTTTCCCTACACGACGCTCT	CAAGCAGAAGACGGCATACGAGATTCAGACACGTGACTGGAGTTCAGACGTGTGCTCTT	AATCGCTGAC	GTGTCTGAAT
AATGATACGGCGACCACCGAGATCTACAGTGACCTACACTCTTTCCCTACACGACGCTCT	CAAGCAGAAGACGGCATACGAGATCGAAGTCAGTGACTGGAGTTCAGACGTGTGCTCTT	AGTGACCTAC	TGACTTCGAT
AATGATACGGCGACCACCGAGATCTACCGAATTGCACACTCTTTCCCTACACGACGCTCT	CAAGCAGAAGACGGCATACGAGATGTGATCCAGTGACTGGAGTTCAGACGTGTGCTCTT	CGAATTGCAC	TGGATCACAT
AATGATACGGCGACCACCGAGATCTACCAAGAAGCACACTCTTTCCCTACACGACGCTCT	CAAGCAGAAGACGGCATACGAGATACTGGTGTGTGACTGGAGTTCAGACGTGTGCTCTT	CAAGAAGCAC	ACACCAGTAT
AATGATACGGCGACCACCGAGATCTACCACCAGTTACACTCTTTCCCTACACGACGCTCT	CAAGCAGAAGACGGCATACGAGATCTAACCTGGTGACTGGAGTTCAGACGTGTGCTCTT	CACCAGTTAC	CAGGTTAGAT
AATGATACGGCGACCACCGAGATCTACGTATTCCGACACTCTTTCCCTACACGACGCTCT	CAAGCAGAAGACGGCATACGAGATAGCCAACTGTGACTGGAGTTCAGACGTGTGCTCTT	GTATTCCGAC	AGTTGGCTAT
AATGATACGGCGACCACCGAGATCTACTTCGAAGCACACTCTTTCCCTACACGACGCTCT	CAAGCAGAAGACGGCATACGAGATCCAGTTGAGTGACTGGAGTTCAGACGTGTGCTCTT	TTCGAAGCAC	TCAACTGGAT
AATGATACGGCGACCACCGAGATCTACAGACCTTGACACTCTTTCCCTACACGACGCTCT	CAAGCAGAAGACGGCATACGAGATAAGTGCAGGTGACTGGAGTTCAGACGTGTGCTCTT	AGACCTTGAC	CTGCACTTAT
AATGATACGGCGACCACCGAGATCTACCCAAGGTTACACTCTTTCCCTACACGACGCTCT	CAAGCAGAAGACGGCATACGAGATAACCGTGTGTGACTGGAGTTCAGACGTGTGCTCTT	CCAAGGTTAC	ACACGGTTAT
AATGATACGGCGACCACCGAGATCTACACGTATGGACACTCTTTCCCTACACGACGCTCT	CAAGCAGAAGACGGCATACGAGATCGCGTATTGTGACTGGAGTTCAGACGTGTGCTCTT	ACGTATGGAC	AATACGCGAT
AATGATACGGCGACCACCGAGATCTACAAGGACCAACACTCTTTCCCTACACGACGCTCT	CAAGCAGAAGACGGCATACGAGATAGTTCGCAGTGACTGGAGTTCAGACGTGTGCTCTT	AAGGACCAAC	TGCGAACTAT
AATGATACGGCGACCACCGAGATCTACTATGCGGTACACTCTTTCCCTACACGACGCTCT	CAAGCAGAAGACGGCATACGAGATTAGTCAGCGTGACTGGAGTTCAGACGTGTGCTCTT	TATGCGGTAC	GCTGACTAAT
AATGATACGGCGACCACCGAGATCTACAAGGAAGGACACTCTTTCCCTACACGACGCTCT	CAAGCAGAAGACGGCATACGAGATAACACCACGTGACTGGAGTTCAGACGTGTGCTCTT	AAGGAAGGAC	GTGGTGTTAT
AATGATACGGCGACCACCGAGATCTACAGCGTGTAACACTCTTTCCCTACACGACGCTCT	CAAGCAGAAGACGGCATACGAGATGTAAGCACGTGACTGGAGTTCAGACGTGTGCTCTT	AGCGTGTAAC	GTGCTTACAT
AATGATACGGCGACCACCGAGATCTACTCTACGCAACACTCTTTCCCTACACGACGCTCT	CAAGCAGAAGACGGCATACGAGATGTCCTTGAGTGACTGGAGTTCAGACGTGTGCTCTT	TCTACGCAAC	TCAAGGACAT
AATGATACGGCGACCACCGAGATCTACTGGCTCTTACACTCTTTCCCTACACGACGCTCT	CAAGCAGAAGACGGCATACGAGATCAGGTTCAGTGACTGGAGTTCAGACGTGTGCTCTT	TGGCTCTTAC	TGAACCTGAT
AATGATACGGCGACCACCGAGATCTACCCTTCCATACACTCTTTCCCTACACGACGCTCT	CAAGCAGAAGACGGCATACGAGATCCAACACTGTGACTGGAGTTCAGACGTGTGCTCTT	CCTTCCATAC	AGTGTTGGAT
AATGATACGGCGACCACCGAGATCTACATACTGGCACACTCTTTCCCTACACGACGCTCT	CAAGCAGAAGACGGCATACGAGATGAGAGTACGTGACTGGAGTTCAGACGTGTGCTCTT	ATACTGGCAC	GTACTCTCAT
AATGATACGGCGACCACCGAGATCTACAACCTACGACACTCTTTCCCTACACGACGCTCT	CAAGCAGAAGACGGCATACGAGATAGATACGGGTGACTGGAGTTCAGACGTGTGCTCTT	AACCTACGAC	CCGTATCTAT
AATGATACGGCGACCACCGAGATCTACCATACTCGACACTCTTTCCCTACACGACGCTCT	CAAGCAGAAGACGGCATACGAGATGTTCTTCGGTGACTGGAGTTCAGACGTGTGCTCTT	CATACTCGAC	CGAAGAACAT
AATGATACGGCGACCACCGAGATCTACTGCACTTGACACTCTTTCCCTACACGACGCTCT	CAAGCAGAAGACGGCATACGAGATATTCCGCTGTGACTGGAGTTCAGACGTGTGCTCTT	TGCACTTGAC	AGCGGAATAT
AATGATACGGCGACCACCGAGATCTACTCACTCGAACACTCTTTCCCTACACGACGCTCT	CAAGCAGAAGACGGCATACGAGATAAGCTCACGTGACTGGAGTTCAGACGTGTGCTCTT	TCACTCGAAC	GTGAGCTTAT
AATGATACGGCGACCACCGAGATCTACCACTGTAGACACTCTTTCCCTACACGACGCTCT	CAAGCAGAAGACGGCATACGAGATTGATCACGGTGACTGGAGTTCAGACGTGTGCTCTT	CACTGTAGAC	CGTGATCAAT
AATGATACGGCGACCACCGAGATCTACGTACGATCACACTCTTTCCCTACACGACGCTCT	CAAGCAGAAGACGGCATACGAGATCAATGCGAGTGACTGGAGTTCAGACGTGTGCTCTT	GTACGATCAC	TCGCATTGAT
AATGATACGGCGACCACCGAGATCTACTGGTGAAGACACTCTTTCCCTACACGACGCTCT	CAAGCAGAAGACGGCATACGAGATATGCGTCAGTGACTGGAGTTCAGACGTGTGCTCTT	TGGTGAAGAC	TGACGCATAT
AATGATACGGCGACCACCGAGATCTACTAGCTGAGACACTCTTTCCCTACACGACGCTCT	CAAGCAGAAGACGGCATACGAGATTACATCGGGTGACTGGAGTTCAGACGTGTGCTCTT	TAGCTGAGAC	CCGATGTAAT
AATGATACGGCGACCACCGAGATCTACAGAGCAGAACACTCTTTCCCTACACGACGCTCT	CAAGCAGAAGACGGCATACGAGATACTGCGAAGTGACTGGAGTTCAGACGTGTGCTCTT	AGAGCAGAAC	TTCGCAGTAT
AATGATACGGCGACCACCGAGATCTACCTTCGGTTACACTCTTTCCCTACACGACGCTCT	CAAGCAGAAGACGGCATACGAGATTCTGTCGTGTGACTGGAGTTCAGACGTGTGCTCTT	CTTCGGTTAC	ACGACAGAAT
AATGATACGGCGACCACCGAGATCTACACAACAGCACACTCTTTCCCTACACGACGCTCT	CAAGCAGAAGACGGCATACGAGATCTCAAGCTGTGACTGGAGTTCAGACGTGTGCTCTT	ACAACAGCAC	AGCTTGAGAT
AATGATACGGCGACCACCGAGATCTACAGCCGTAAACACTCTTTCCCTACACGACGCTCT	CAAGCAGAAGACGGCATACGAGATAACCACTCGTGACTGGAGTTCAGACGTGTGCTCTT	AGCCGTAAAC	GAGTGGTTAT
AATGATACGGCGACCACCGAGATCTACCTCTTGTCACACTCTTTCCCTACACGACGCTCT	CAAGCAGAAGACGGCATACGAGATCTTACAGCGTGACTGGAGTTCAGACGTGTGCTCTT	CTCTTGTCAC	GCTGTAAGAT
AATGATACGGCGACCACCGAGATCTACCAGATCCTACACTCTTTCCCTACACGACGCTCT	CAAGCAGAAGACGGCATACGAGATAGTCTTGGGTGACTGGAGTTCAGACGTGTGCTCTT	CAGATCCTAC	CCAAGACTAT
AATGATACGGCGACCACCGAGATCTACGATGCTACACACTCTTTCCCTACACGACGCTCT	CAAGCAGAAGACGGCATACGAGATCACGCAATGTGACTGGAGTTCAGACGTGTGCTCTT	GATGCTACAC	ATTGCGTGAT
AATGATACGGCGACCACCGAGATCTACAGGAACACACACTCTTTCCCTACACGACGCTCT	CAAGCAGAAGACGGCATACGAGATAGCTTCAGGTGACTGGAGTTCAGACGTGTGCTCTT	AGGAACACAC	CTGAAGCTAT
AATGATACGGCGACCACCGAGATCTACACCATCCTACACTCTTTCCCTACACGACGCTCT	CAAGCAGAAGACGGCATACGAGATCCTCGTTAGTGACTGGAGTTCAGACGTGTGCTCTT	ACCATCCTAC	TAACGAGGAT
AATGATACGGCGACCACCGAGATCTACGAACGTGAACACTCTTTCCCTACACGACGCTCT	CAAGCAGAAGACGGCATACGAGATTGAGACGAGTGACTGGAGTTCAGACGTGTGCTCTT	GAACGTGAAC	TCGTCTCAAT
AATGATACGGCGACCACCGAGATCTACTAGAACGCACACTCTTTCCCTACACGACGCTCT	CAAGCAGAAGACGGCATACGAGATCACAGGAAGTGACTGGAGTTCAGACGTGTGCTCTT	TAGAACGCAC	TTCCTGTGAT
AATGATACGGCGACCACCGAGATCTACAACCAGAGACACTCTTTCCCTACACGACGCTCT	CAAGCAGAAGACGGCATACGAGATACTCAACGGTGACTGGAGTTCAGACGTGTGCTCTT	AACCAGAGAC	CGTTGAGTAT
AATGATACGGCGACCACCGAGATCTACCGACCTAAACACTCTTTCCCTACACGACGCTCT	CAAGCAGAAGACGGCATACGAGATAAGCGACTGTGACTGGAGTTCAGACGTGTGCTCTT	CGACCTAAAC	AGTCGCTTAT
AATGATACGGCGACCACCGAGATCTACCTCTCAGAACACTCTTTCCCTACACGACGCTCT	CAAGCAGAAGACGGCATACGAGATCCTACCTAGTGACTGGAGTTCAGACGTGTGCTCTT	CTCTCAGAAC	TAGGTAGGAT
AATGATACGGCGACCACCGAGATCTACAGGCTGAAACACTCTTTCCCTACACGACGCTCT	CAAGCAGAAGACGGCATACGAGATATCTCCTGGTGACTGGAGTTCAGACGTGTGCTCTT	AGGCTGAAAC	CAGGAGATAT
AATGATACGGCGACCACCGAGATCTACATCGGAGAACACTCTTTCCCTACACGACGCTCT	CAAGCAGAAGACGGCATACGAGATTCACGATGGTGACTGGAGTTCAGACGTGTGCTCTT	ATCGGAGAAC	CATCGTGAAT
AATGATACGGCGACCACCGAGATCTACGATACCTGACACTCTTTCCCTACACGACGCTCT	CAAGCAGAAGACGGCATACGAGATCCACAACAGTGACTGGAGTTCAGACGTGTGCTCTT	GATACCTGAC	TGTTGTGGAT
AATGATACGGCGACCACCGAGATCTACTCCTGACTACACTCTTTCCCTACACGACGCTCT	CAAGCAGAAGACGGCATACGAGATAGGTCTGTGTGACTGGAGTTCAGACGTGTGCTCTT	TCCTGACTAC	ACAGACCTAT
AATGATACGGCGACCACCGAGATCTACTCAGCCTTACACTCTTTCCCTACACGACGCTCT	CAAGCAGAAGACGGCATACGAGATAGAAGGACGTGACTGGAGTTCAGACGTGTGCTCTT	TCAGCCTTAC	GTCCTTCTAT
AATGATACGGCGACCACCGAGATCTACAAGCATCGACACTCTTTCCCTACACGACGCTCT	CAAGCAGAAGACGGCATACGAGATGCGTATCAGTGACTGGAGTTCAGACGTGTGCTCTT	AAGCATCGAC	TGATACGCAT
AATGATACGGCGACCACCGAGATCTACGCCAATACACACTCTTTCCCTACACGACGCTCT	CAAGCAGAAGACGGCATACGAGATCAACACAGGTGACTGGAGTTCAGACGTGTGCTCTT	GCCAATACAC	CTGTGTTGAT
AATGATACGGCGACCACCGAGATCTACGACACAGTACACTCTTTCCCTACACGACGCTCT	CAAGCAGAAGACGGCATACGAGATTCCACGTTGTGACTGGAGTTCAGACGTGTGCTCTT	GACACAGTAC	AACGTGGAAT
AATGATACGGCGACCACCGAGATCTACAAGAGGCAACACTCTTTCCCTACACGACGCTCT	CAAGCAGAAGACGGCATACGAGATATCGCAACGTGACTGGAGTTCAGACGTGTGCTCTT	AAGAGGCAAC	GTTGCGATAT
AATGATACGGCGACCACCGAGATCTACGAAGACTGACACTCTTTCCCTACACGACGCTCT	CAAGCAGAAGACGGCATACGAGATACGTCGTTGTGACTGGAGTTCAGACGTGTGCTCTT	GAAGACTGAC	AACGACGTAT
AATGATACGGCGACCACCGAGATCTACCCGTTATGACACTCTTTCCCTACACGACGCTCT	CAAGCAGAAGACGGCATACGAGATCGAATACGGTGACTGGAGTTCAGACGTGTGCTCTT	CCGTTATGAC	CGTATTCGAT
AATGATACGGCGACCACCGAGATCTACCTAGCAGTACACTCTTTCCCTACACGACGCTCT	CAAGCAGAAGACGGCATACGAGATTGCTTGCTGTGACTGGAGTTCAGACGTGTGCTCTT	CTAGCAGTAC	AGCAAGCAAT
AATGATACGGCGACCACCGAGATCTACGCCAGAATACACTCTTTCCCTACACGACGCTCT	CAAGCAGAAGACGGCATACGAGATCTCGAACAGTGACTGGAGTTCAGACGTGTGCTCTT	GCCAGAATAC	TGTTCGAGAT
AATGATACGGCGACCACCGAGATCTACCGAGAGAAACACTCTTTCCCTACACGACGCTCT	CAAGCAGAAGACGGCATACGAGATACATGGAGGTGACTGGAGTTCAGACGTGTGCTCTT	CGAGAGAAAC	CTCCATGTAT
AATGATACGGCGACCACCGAGATCTACAACTCGGAACACTCTTTCCCTACACGACGCTCT	CAAGCAGAAGACGGCATACGAGATACAAGACGGTGACTGGAGTTCAGACGTGTGCTCTT	AACTCGGAAC	CGTCTTGTAT
AATGATACGGCGACCACCGAGATCTACACAGTTCGACACTCTTTCCCTACACGACGCTCT	CAAGCAGAAGACGGCATACGAGATCGCCTTATGTGACTGGAGTTCAGACGTGTGCTCTT	ACAGTTCGAC	ATAAGGCGAT
AATGATACGGCGACCACCGAGATCTACTGACCGTTACACTCTTTCCCTACACGACGCTCT	CAAGCAGAAGACGGCATACGAGATAGCAGACAGTGACTGGAGTTCAGACGTGTGCTCTT	TGACCGTTAC	TGTCTGCTAT
AATGATACGGCGACCACCGAGATCTACCATCTGCTACACTCTTTCCCTACACGACGCTCT	CAAGCAGAAGACGGCATACGAGATGTTAAGCGGTGACTGGAGTTCAGACGTGTGCTCTT	CATCTGCTAC	CGCTTAACAT
AATGATACGGCGACCACCGAGATCTACCGCTGATAACACTCTTTCCCTACACGACGCTCT	CAAGCAGAAGACGGCATACGAGATCATGGATCGTGACTGGAGTTCAGACGTGTGCTCTT	CGCTGATAAC	GATCCATGAT
AATGATACGGCGACCACCGAGATCTACTCGTCTGAACACTCTTTCCCTACACGACGCTCT	CAAGCAGAAGACGGCATACGAGATACAGAGGTGTGACTGGAGTTCAGACGTGTGCTCTT	TCGTCTGAAC	ACCTCTGTAT
AATGATACGGCGACCACCGAGATCTACCACATGGTACACTCTTTCCCTACACGACGCTCT	CAAGCAGAAGACGGCATACGAGATTAAGTGGCGTGACTGGAGTTCAGACGTGTGCTCTT	CACATGGTAC	GCCACTTAAT
AATGATACGGCGACCACCGAGATCTACCGAGTTAGACACTCTTTCCCTACACGACGCTCT	CAAGCAGAAGACGGCATACGAGATAGTCAGGTGTGACTGGAGTTCAGACGTGTGCTCTT	CGAGTTAGAC	ACCTGACTAT
AATGATACGGCGACCACCGAGATCTACAGCTAAGCACACTCTTTCCCTACACGACGCTCT	CAAGCAGAAGACGGCATACGAGATGCCTTAACGTGACTGGAGTTCAGACGTGTGCTCTT	AGCTAAGCAC	GTTAAGGCAT
AATGATACGGCGACCACCGAGATCTACGTTCCATGACACTCTTTCCCTACACGACGCTCT	CAAGCAGAAGACGGCATACGAGATGTTGGCATGTGACTGGAGTTCAGACGTGTGCTCTT	GTTCCATGAC	ATGCCAACAT
AATGATACGGCGACCACCGAGATCTACGCATCCTAACACTCTTTCCCTACACGACGCTCT	CAAGCAGAAGACGGCATACGAGATCAACCTCTGTGACTGGAGTTCAGACGTGTGCTCTT	GCATCCTAAC	AGAGGTTGAT
AATGATACGGCGACCACCGAGATCTACCCATGAACACACTCTTTCCCTACACGACGCTCT	CAAGCAGAAGACGGCATACGAGATTGGATGGTGTGACTGGAGTTCAGACGTGTGCTCTT	CCATGAACAC	ACCATCCAAT
AATGATACGGCGACCACCGAGATCTACATCCACGAACACTCTTTCCCTACACGACGCTCT	CAAGCAGAAGACGGCATACGAGATCTATCCACGTGACTGGAGTTCAGACGTGTGCTCTT	ATCCACGAAC	GTGGATAGAT
AATGATACGGCGACCACCGAGATCTACGAGAAGGTACACTCTTTCCCTACACGACGCTCT	CAAGCAGAAGACGGCATACGAGATGATCTCAGGTGACTGGAGTTCAGACGTGTGCTCTT	GAGAAGGTAC	CTGAGATCAT
AATGATACGGCGACCACCGAGATCTACAGGCAATGACACTCTTTCCCTACACGACGCTCT	CAAGCAGAAGACGGCATACGAGATACGATCAGGTGACTGGAGTTCAGACGTGTGCTCTT	AGGCAATGAC	CTGATCGTAT
AATGATACGGCGACCACCGAGATCTACTCACCTAGACACTCTTTCCCTACACGACGCTCT	CAAGCAGAAGACGGCATACGAGATTCGAGAGTGTGACTGGAGTTCAGACGTGTGCTCTT	TCACCTAGAC	ACTCTCGAAT
AATGATACGGCGACCACCGAGATCTACCATACGGAACACTCTTTCCCTACACGACGCTCT	CAAGCAGAAGACGGCATACGAGATCTAGCTCAGTGACTGGAGTTCAGACGTGTGCTCTT	CATACGGAAC	TGAGCTAGAT

AATGATACGGCGACCACCGAGATCTACACGTCATCGTACACTCTTTCCCTACACGACGCTCT	CAAGCAGAAGACGGCATACGAGATATCGTCTCGTGACTGGAGTTCAGACGTGTGCTCTT	GTCATCGTAC	GAGACGATAT
AATGATACGGCGACCACCGAGATCTACACTTACCGACACACTCTTTCCCTACACGACGCTCT	CAAGCAGAAGACGGCATACGAGATTCGACAAGGTGACTGGAGTTCAGACGTGTGCTCTT	TTACCGACAC	CTTGTCGAAT
AATGATACGGCGACCACCGAGATCTACACACCTTCGAACACTCTTTCCCTACACGACGCTCT	CAAGCAGAAGACGGCATACGAGATCCTTGGAAGTGACTGGAGTTCAGACGTGTGCTCTT	ACCTTCGAAC	TTCCAAGGAT
AATGATACGGCGACCACCGAGATCTACACACGCTTCTACACTCTTTCCCTACACGACGCTCT	CAAGCAGAAGACGGCATACGAGATATCATGCGGTGACTGGAGTTCAGACGTGTGCTCTT	ACGCTTCTAC	CGCATGATAT
AATGATACGGCGACCACCGAGATCTACACGAGTAGAGACACTCTTTCCCTACACGACGCTCT	CAAGCAGAAGACGGCATACGAGATTGTTCCGTGTGACTGGAGTTCAGACGTGTGCTCTT	GAGTAGAGAC	ACGGAACAAT
AATGATACGGCGACCACCGAGATCTACACATGCCTAGACACTCTTTCCCTACACGACGCTCT	CAAGCAGAAGACGGCATACGAGATATTAGCCGGTGACTGGAGTTCAGACGTGTGCTCTT	ATGCCTAGAC	CGGCTAATAT
AATGATACGGCGACCACCGAGATCTACACCAACTCCAACACTCTTTCCCTACACGACGCTCT	CAAGCAGAAGACGGCATACGAGATCGATCGATGTGACTGGAGTTCAGACGTGTGCTCTT	CAACTCCAAC	ATCGATCGAT
AATGATACGGCGACCACCGAGATCTACACAAGTCCTCACACTCTTTCCCTACACGACGCTCT	CAAGCAGAAGACGGCATACGAGATGATCTTGCGTGACTGGAGTTCAGACGTGTGCTCTT	AAGTCCTCAC	GCAAGATCAT
AATGATACGGCGACCACCGAGATCTACACGTCGATTGACACTCTTTCCCTACACGACGCTCT	CAAGCAGAAGACGGCATACGAGATAGGATAGCGTGACTGGAGTTCAGACGTGTGCTCTT	GTCGATTGAC	GCTATCCTAT
AATGATACGGCGACCACCGAGATCTACACGCGTTAGAACACTCTTTCCCTACACGACGCTCT	CAAGCAGAAGACGGCATACGAGATGTAGCGTAGTGACTGGAGTTCAGACGTGTGCTCTT	GCGTTAGAAC	TACGCTACAT
AATGATACGGCGACCACCGAGATCTACACTTGCGAGAACACTCTTTCCCTACACGACGCTCT	CAAGCAGAAGACGGCATACGAGATAGAGTCCAGTGACTGGAGTTCAGACGTGTGCTCTT	TTGCGAGAAC	TGGACTCTAT
AATGATACGGCGACCACCGAGATCTACACACACCGATACACTCTTTCCCTACACGACGCTCT	CAAGCAGAAGACGGCATACGAGATGCTACTCTGTGACTGGAGTTCAGACGTGTGCTCTT	ACACCGATAC	AGAGTAGCAT
AATGATACGGCGACCACCGAGATCTACACCGTATCTCACACTCTTTCCCTACACGACGCTCT	CAAGCAGAAGACGGCATACGAGATCTCTGGATGTGACTGGAGTTCAGACGTGTGCTCTT	CGTATCTCAC	ATCCAGAGAT
AATGATACGGCGACCACCGAGATCTACACAAGGAGACACACTCTTTCCCTACACGACGCTCT	CAAGCAGAAGACGGCATACGAGATAGATCGTCGTGACTGGAGTTCAGACGTGTGCTCTT	AAGGAGACAC	GACGATCTAT
AATGATACGGCGACCACCGAGATCTACACTGTCGACTACACTCTTTCCCTACACGACGCTCT	CAAGCAGAAGACGGCATACGAGATGCTCAGTTGTGACTGGAGTTCAGACGTGTGCTCTT	TGTCGACTAC	AACTGAGCAT
AATGATACGGCGACCACCGAGATCTACACTCAATCCGACACTCTTTCCCTACACGACGCTCT	CAAGCAGAAGACGGCATACGAGATGTCCTAAGGTGACTGGAGTTCAGACGTGTGCTCTT	TCAATCCGAC	CTTAGGACAT
AATGATACGGCGACCACCGAGATCTACACGACTTGTGACACTCTTTCCCTACACGACGCTCT	CAAGCAGAAGACGGCATACGAGATTATGGCACGTGACTGGAGTTCAGACGTGTGCTCTT	GACTTGTGAC	GTGCCATAAT
AATGATACGGCGACCACCGAGATCTACACCCGATGTAACACTCTTTCCCTACACGACGCTCT	CAAGCAGAAGACGGCATACGAGATTCGGATTCGTGACTGGAGTTCAGACGTGTGCTCTT	CCGATGTAAC	GAATCCGAAT
AATGATACGGCGACCACCGAGATCTACACTAGGAGCTACACTCTTTCCCTACACGACGCTCT	CAAGCAGAAGACGGCATACGAGATAACAGCGAGTGACTGGAGTTCAGACGTGTGCTCTT	TAGGAGCTAC	TCGCTGTTAT
AATGATACGGCGACCACCGAGATCTACACCAACGAGTACACTCTTTCCCTACACGACGCTCT	CAAGCAGAAGACGGCATACGAGATCCAACGAAGTGACTGGAGTTCAGACGTGTGCTCTT	CAACGAGTAC	TTCGTTGGAT
AATGATACGGCGACCACCGAGATCTACACTGTGTCAGACACTCTTTCCCTACACGACGCTCT	CAAGCAGAAGACGGCATACGAGATCAGTGCTTGTGACTGGAGTTCAGACGTGTGCTCTT	TGTGTCAGAC	AAGCACTGAT
AATGATACGGCGACCACCGAGATCTACACCTAGGTTGACACTCTTTCCCTACACGACGCTCT	CAAGCAGAAGACGGCATACGAGATGATCAAGGGTGACTGGAGTTCAGACGTGTGCTCTT	CTAGGTTGAC	CCTTGATCAT
AATGATACGGCGACCACCGAGATCTACACGTGTCCTTACACTCTTTCCCTACACGACGCTCT	CAAGCAGAAGACGGCATACGAGATTCTTCGACGTGACTGGAGTTCAGACGTGTGCTCTT	GTGTCCTTAC	GTCGAAGAAT
AATGATACGGCGACCACCGAGATCTACACTACCTGCAACACTCTTTCCCTACACGACGCTCT	CAAGCAGAAGACGGCATACGAGATATCGTGGTGTGACTGGAGTTCAGACGTGTGCTCTT	TACCTGCAAC	ACCACGATAT
AATGATACGGCGACCACCGAGATCTACACCCTTAGGTACACTCTTTCCCTACACGACGCTCT	CAAGCAGAAGACGGCATACGAGATCGGTAATCGTGACTGGAGTTCAGACGTGTGCTCTT	CCTTAGGTAC	GATTACCGAT
AATGATACGGCGACCACCGAGATCTACACCACAGACTACACTCTTTCCCTACACGACGCTCT	CAAGCAGAAGACGGCATACGAGATAGTTGTGCGTGACTGGAGTTCAGACGTGTGCTCTT	CACAGACTAC	GCACAACTAT
AATGATACGGCGACCACCGAGATCTACACTCGAACCTACACTCTTTCCCTACACGACGCTCT	CAAGCAGAAGACGGCATACGAGATAATGACGCGTGACTGGAGTTCAGACGTGTGCTCTT	TCGAACCTAC	GCGTCATTAT
AATGATACGGCGACCACCGAGATCTACACGCATAGTCACACTCTTTCCCTACACGACGCTCT	CAAGCAGAAGACGGCATACGAGATTACCGGATGTGACTGGAGTTCAGACGTGTGCTCTT	GCATAGTCAC	ATCCGGTAAT
AATGATACGGCGACCACCGAGATCTACACCTCCTGAAACACTCTTTCCCTACACGACGCTCT	CAAGCAGAAGACGGCATACGAGATTTGCAACGGTGACTGGAGTTCAGACGTGTGCTCTT	CTCCTGAAAC	CGTTGCAAAT
AATGATACGGCGACCACCGAGATCTACACAACGCACAACACTCTTTCCCTACACGACGCTCT	CAAGCAGAAGACGGCATACGAGATCACTTCACGTGACTGGAGTTCAGACGTGTGCTCTT	AACGCACAAC	GTGAAGTGAT
AATGATACGGCGACCACCGAGATCTACACTAGTCTCGACACTCTTTCCCTACACGACGCTCT	CAAGCAGAAGACGGCATACGAGATTAGCCATGGTGACTGGAGTTCAGACGTGTGCTCTT	TAGTCTCGAC	CATGGCTAAT
AATGATACGGCGACCACCGAGATCTACACACTCTGAGACACTCTTTCCCTACACGACGCTCT	CAAGCAGAAGACGGCATACGAGATACAGGCATGTGACTGGAGTTCAGACGTGTGCTCTT	ACTCTGAGAC	ATGCCTGTAT
AATGATACGGCGACCACCGAGATCTACACGTTATGGCACACTCTTTCCCTACACGACGCTCT	CAAGCAGAAGACGGCATACGAGATAGGTGTTGGTGACTGGAGTTCAGACGTGTGCTCTT	GTTATGGCAC	CAACACCTAT
AATGATACGGCGACCACCGAGATCTACACCTCGGTAAACACTCTTTCCCTACACGACGCTCT	CAAGCAGAAGACGGCATACGAGATCAGTCACAGTGACTGGAGTTCAGACGTGTGCTCTT	CTCGGTAAAC	TGTGACTGAT
AATGATACGGCGACCACCGAGATCTACACTACAGAGCACACTCTTTCCCTACACGACGCTCT	CAAGCAGAAGACGGCATACGAGATTCGATGACGTGACTGGAGTTCAGACGTGTGCTCTT	TACAGAGCAC	GTCATCGAAT
AATGATACGGCGACCACCGAGATCTACACGCATAACGACACTCTTTCCCTACACGACGCTCT	CAAGCAGAAGACGGCATACGAGATGAAGTGCTGTGACTGGAGTTCAGACGTGTGCTCTT	GCATAACGAC	AGCACTTCAT
AATGATACGGCGACCACCGAGATCTACACGATCAGACACACTCTTTCCCTACACGACGCTCT	CAAGCAGAAGACGGCATACGAGATCTTCCTTCGTGACTGGAGTTCAGACGTGTGCTCTT	GATCAGACAC	GAAGGAAGAT
AATGATACGGCGACCACCGAGATCTACACCGCAACTAACACTCTTTCCCTACACGACGCTCT	CAAGCAGAAGACGGCATACGAGATCGAACAACGTGACTGGAGTTCAGACGTGTGCTCTT	CGCAACTAAC	GTTGTTCGAT
AATGATACGGCGACCACCGAGATCTACACTCCGATCAACACTCTTTCCCTACACGACGCTCT	CAAGCAGAAGACGGCATACGAGATAACAACCGGTGACTGGAGTTCAGACGTGTGCTCTT	TCCGATCAAC	CGGTTGTTAT
AATGATACGGCGACCACCGAGATCTACACCAACTTGGACACTCTTTCCCTACACGACGCTCT	CAAGCAGAAGACGGCATACGAGATACCTCAGTGTGACTGGAGTTCAGACGTGTGCTCTT	CAACTTGGAC	ACTGAGGTAT
AATGATACGGCGACCACCGAGATCTACACTCAGTAGGACACTCTTTCCCTACACGACGCTCT	CAAGCAGAAGACGGCATACGAGATCGTCTTCAGTGACTGGAGTTCAGACGTGTGCTCTT	TCAGTAGGAC	TGAAGACGAT
AATGATACGGCGACCACCGAGATCTACACACAGCAAGACACTCTTTCCCTACACGACGCTCT	CAAGCAGAAGACGGCATACGAGATTGCGTAACGTGACTGGAGTTCAGACGTGTGCTCTT	ACAGCAAGAC	GTTACGCAAT
AATGATACGGCGACCACCGAGATCTACACGAATGGCAACACTCTTTCCCTACACGACGCTCT	CAAGCAGAAGACGGCATACGAGATAACACGCTGTGACTGGAGTTCAGACGTGTGCTCTT	GAATGGCAAC	AGCGTGTTAT
AATGATACGGCGACCACCGAGATCTACACCGGATCAAACACTCTTTCCCTACACGACGCTCT	CAAGCAGAAGACGGCATACGAGATACTCGATCGTGACTGGAGTTCAGACGTGTGCTCTT	CGGATCAAAC	GATCGAGTAT
AATGATACGGCGACCACCGAGATCTACACACTGCACTACACTCTTTCCCTACACGACGCTCT	CAAGCAGAAGACGGCATACGAGATTGAGCTGTGTGACTGGAGTTCAGACGTGTGCTCTT	ACTGCACTAC	ACAGCTCAAT
AATGATACGGCGACCACCGAGATCTACACCCTAAGTCACACTCTTTCCCTACACGACGCTCT	CAAGCAGAAGACGGCATACGAGATTACTGCTCGTGACTGGAGTTCAGACGTGTGCTCTT	CCTAAGTCAC	GAGCAGTAAT
AATGATACGGCGACCACCGAGATCTACACTTCGTACGACACTCTTTCCCTACACGACGCTCT	CAAGCAGAAGACGGCATACGAGATGACGAACTGTGACTGGAGTTCAGACGTGTGCTCTT	TTCGTACGAC	AGTTCGTCAT
AATGATACGGCGACCACCGAGATCTACACTCCTGGTAACACTCTTTCCCTACACGACGCTCT	CAAGCAGAAGACGGCATACGAGATCTTCGCAAGTGACTGGAGTTCAGACGTGTGCTCTT	TCCTGGTAAC	TTGCGAAGAT
AATGATACGGCGACCACCGAGATCTACACCATTGACGACACTCTTTCCCTACACGACGCTCT	CAAGCAGAAGACGGCATACGAGATATGGCGATGTGACTGGAGTTCAGACGTGTGCTCTT	CATTGACGAC	ATCGCCATAT
AATGATACGGCGACCACCGAGATCTACACACCTCTTCACACTCTTTCCCTACACGACGCTCT	CAAGCAGAAGACGGCATACGAGATACATGCCAGTGACTGGAGTTCAGACGTGTGCTCTT	ACCTCTTCAC	TGGCATGTAT
AATGATACGGCGACCACCGAGATCTACACCATTCGTCACACTCTTTCCCTACACGACGCTCT	CAAGCAGAAGACGGCATACGAGATGTCAACAGGTGACTGGAGTTCAGACGTGTGCTCTT	CATTCGTCAC	CTGTTGACAT
AATGATACGGCGACCACCGAGATCTACACTTCCTCCTACACTCTTTCCCTACACGACGCTCT	CAAGCAGAAGACGGCATACGAGATGTGGTATGGTGACTGGAGTTCAGACGTGTGCTCTT	TTCCTCCTAC	CATACCACAT
AATGATACGGCGACCACCGAGATCTACACGCTGTAAGACACTCTTTCCCTACACGACGCTCT	CAAGCAGAAGACGGCATACGAGATCCAACTTCGTGACTGGAGTTCAGACGTGTGCTCTT	GCTGTAAGAC	GAAGTTGGAT
AATGATACGGCGACCACCGAGATCTACACGACATCTCACACTCTTTCCCTACACGACGCTCT	CAAGCAGAAGACGGCATACGAGATGACGTCATGTGACTGGAGTTCAGACGTGTGCTCTT	GACATCTCAC	ATGACGTCAT
AATGATACGGCGACCACCGAGATCTACACCAACCGTAACACTCTTTCCCTACACGACGCTCT	CAAGCAGAAGACGGCATACGAGATACGTCCAAGTGACTGGAGTTCAGACGTGTGCTCTT	CAACCGTAAC	TTGGACGTAT
AATGATACGGCGACCACCGAGATCTACACTGCGATAGACACTCTTTCCCTACACGACGCTCT	CAAGCAGAAGACGGCATACGAGATGATCCACTGTGACTGGAGTTCAGACGTGTGCTCTT	TGCGATAGAC	AGTGGATCAT
AATGATACGGCGACCACCGAGATCTACACTGGTTCGAACACTCTTTCCCTACACGACGCTCT	CAAGCAGAAGACGGCATACGAGATAGCCTATCGTGACTGGAGTTCAGACGTGTGCTCTT	TGGTTCGAAC	GATAGGCTAT
AATGATACGGCGACCACCGAGATCTACACAAGCGTTCACACTCTTTCCCTACACGACGCTCT	CAAGCAGAAGACGGCATACGAGATAGCTACCAGTGACTGGAGTTCAGACGTGTGCTCTT	AAGCGTTCAC	TGGTAGCTAT
AATGATACGGCGACCACCGAGATCTACACCGATTCTGACACTCTTTCCCTACACGACGCTCT	CAAGCAGAAGACGGCATACGAGATAGATTGCGGTGACTGGAGTTCAGACGTGTGCTCTT	CGATTCTGAC	CGCAATCTAT
AATGATACGGCGACCACCGAGATCTACACGCAACCATACACTCTTTCCCTACACGACGCTCT	CAAGCAGAAGACGGCATACGAGATCACACATCGTGACTGGAGTTCAGACGTGTGCTCTT	GCAACCATAC	GATGTGTGAT
AATGATACGGCGACCACCGAGATCTACACAATCCAGCACACTCTTTCCCTACACGACGCTCT	CAAGCAGAAGACGGCATACGAGATGAGCAATCGTGACTGGAGTTCAGACGTGTGCTCTT	AATCCAGCAC	GATTGCTCAT
AATGATACGGCGACCACCGAGATCTACACAGTGCATCACACTCTTTCCCTACACGACGCTCT	CAAGCAGAAGACGGCATACGAGATATAGAGCGGTGACTGGAGTTCAGACGTGTGCTCTT	AGTGCATCAC	CGCTCTATAT
AATGATACGGCGACCACCGAGATCTACACGCATTGGTACACTCTTTCCCTACACGACGCTCT	CAAGCAGAAGACGGCATACGAGATGACCGATAGTGACTGGAGTTCAGACGTGTGCTCTT	GCATTGGTAC	TATCGGTCAT
AATGATACGGCGACCACCGAGATCTACACCTTAGGACACACTCTTTCCCTACACGACGCTCT	CAAGCAGAAGACGGCATACGAGATCAGACGTTGTGACTGGAGTTCAGACGTGTGCTCTT	CTTAGGACAC	AACGTCTGAT
AATGATACGGCGACCACCGAGATCTACACATAGTCGGACACTCTTTCCCTACACGACGCTCT	CAAGCAGAAGACGGCATACGAGATCTGAACGTGTGACTGGAGTTCAGACGTGTGCTCTT	ATAGTCGGAC	ACGTTCAGAT
AATGATACGGCGACCACCGAGATCTACACGAGACCAAACACTCTTTCCCTACACGACGCTCT	CAAGCAGAAGACGGCATACGAGATTTGGACTGGTGACTGGAGTTCAGACGTGTGCTCTT	GAGACCAAAC	CAGTCCAAAT
AATGATACGGCGACCACCGAGATCTACACAACAAGGCACACTCTTTCCCTACACGACGCTCT	CAAGCAGAAGACGGCATACGAGATGTCTGCAAGTGACTGGAGTTCAGACGTGTGCTCTT	AACAAGGCAC	TTGCAGACAT
AATGATACGGCGACCACCGAGATCTACACCCAGTATCACACTCTTTCCCTACACGACGCTCT	CAAGCAGAAGACGGCATACGAGATCCACATTGGTGACTGGAGTTCAGACGTGTGCTCTT	CCAGTATCAC	CAATGTGGAT
AATGATACGGCGACCACCGAGATCTACACCCTCGAATACACTCTTTCCCTACACGACGCTCT	CAAGCAGAAGACGGCATACGAGATGATGGAGTGTGACTGGAGTTCAGACGTGTGCTCTT	CCTCGAATAC	ACTCCATCAT
AATGATACGGCGACCACCGAGATCTACACCAACTGACACACTCTTTCCCTACACGACGCTCT	CAAGCAGAAGACGGCATACGAGATAGGTCAACGTGACTGGAGTTCAGACGTGTGCTCTT	CAACTGACAC	GTTGACCTAT
AATGATACGGCGACCACCGAGATCTACACTGCTCTACACACTCTTTCCCTACACGACGCTCT	CAAGCAGAAGACGGCATACGAGATTACACACGGTGACTGGAGTTCAGACGTGTGCTCTT	TGCTCTACAC	CGTGTGTAAT
AATGATACGGCGACCACCGAGATCTACACCATCACGTACACTCTTTCCCTACACGACGCTCT	CAAGCAGAAGACGGCATACGAGATCAAGTCGTGTGACTGGAGTTCAGACGTGTGCTCTT	CATCACGTAC	ACGACTTGAT
AATGATACGGCGACCACCGAGATCTACACGCCACTTAACACTCTTTCCCTACACGACGCTCT	CAAGCAGAAGACGGCATACGAGATAGCTAGTGGTGACTGGAGTTCAGACGTGTGCTCTT	GCCACTTAAC	CACTAGCTAT
AATGATACGGCGACCACCGAGATCTACACGCTTCACAACACTCTTTCCCTACACGACGCTCT	CAAGCAGAAGACGGCATACGAGATCTCCTAGTGTGACTGGAGTTCAGACGTGTGCTCTT	GCTTCACAAC	ACTAGGAGAT
AATGATACGGCGACCACCGAGATCTACACACCGAATGACACTCTTTCCCTACACGACGCTCT	CAAGCAGAAGACGGCATACGAGATACTCCTACGTGACTGGAGTTCAGACGTGTGCTCTT	ACCGAATGAC	GTAGGAGTAT
AATGATACGGCGACCACCGAGATCTACACCTCACCAAACACTCTTTCCCTACACGACGCTCT	CAAGCAGAAGACGGCATACGAGATCAATCAGGGTGACTGGAGTTCAGACGTGTGCTCTT	CTCACCAAAC	CCTGATTGAT
AATGATACGGCGACCACCGAGATCTACACCAGAACTGACACTCTTTCCCTACACGACGCTCT	CAAGCAGAAGACGGCATACGAGATTCGTGCATGTGACTGGAGTTCAGACGTGTGCTCTT	CAGAACTGAC	ATGCACGAAT
AATGATACGGCGACCACCGAGATCTACACAGAAGCCTACACTCTTTCCCTACACGACGCTCT	CAAGCAGAAGACGGCATACGAGATTAACGTCGGTGACTGGAGTTCAGACGTGTGCTCTT	AGAAGCCTAC	CGACGTTAAT
AATGATACGGCGACCACCGAGATCTACACCACGATTCACACTCTTTCCCTACACGACGCTCT	CAAGCAGAAGACGGCATACGAGATAAGGCGTAGTGACTGGAGTTCAGACGTGTGCTCTT	CACGATTCAC	TACGCCTTAT
AATGATACGGCGACCACCGAGATCTACACAAGCTGGTACACTCTTTCCCTACACGACGCTCT	CAAGCAGAAGACGGCATACGAGATTCTTACGGGTGACTGGAGTTCAGACGTGTGCTCTT	AAGCTGGTAC	CCGTAAGAAT
AATGATACGGCGACCACCGAGATCTACACGCAATGAGACACTCTTTCCCTACACGACGCTCT	CAAGCAGAAGACGGCATACGAGATCGTGTGATGTGACTGGAGTTCAGACGTGTGCTCTT	GCAATGAGAC	ATCACACGAT
AATGATACGGCGACCACCGAGATCTACACCTCTATCGACACTCTTTCCCTACACGACGCTCT	CAAGCAGAAGACGGCATACGAGATAACAGGTGGTGACTGGAGTTCAGACGTGTGCTCTT	CTCTATCGAC	CACCTGTTAT
AATGATACGGCGACCACCGAGATCTACACACTCTCCAACACTCTTTCCCTACACGACGCTCT	CAAGCAGAAGACGGCATACGAGATAGTCGAAGGTGACTGGAGTTCAGACGTGTGCTCTT	ACTCTCCAAC	CTTCGACTAT
AATGATACGGCGACCACCGAGATCTACACCAGCATACACACTCTTTCCCTACACGACGCTCT	CAAGCAGAAGACGGCATACGAGATTGGAAGCAGTGACTGGAGTTCAGACGTGTGCTCTT	CAGCATACAC	TGCTTCCAAT
AATGATACGGCGACCACCGAGATCTACACTACTCCAGACACTCTTTCCCTACACGACGCTCT	CAAGCAGAAGACGGCATACGAGATCTCGTTCTGTGACTGGAGTTCAGACGTGTGCTCTT	TACTCCAGAC	AGAACGAGAT
AATGATACGGCGACCACCGAGATCTACACGAGGCATTACACTCTTTCCCTACACGACGCTCT	CAAGCAGAAGACGGCATACGAGATACGAGAACGTGACTGGAGTTCAGACGTGTGCTCTT	GAGGCATTAC	GTTCTCGTAT
AATGATACGGCGACCACCGAGATCTACACACACCTCAACACTCTTTCCCTACACGACGCTCT	CAAGCAGAAGACGGCATACGAGATAAGCCTGAGTGACTGGAGTTCAGACGTGTGCTCTT	ACACCTCAAC	TCAGGCTTAT
AATGATACGGCGACCACCGAGATCTACACCGCAATGTACACTCTTTCCCTACACGACGCTCT	CAAGCAGAAGACGGCATACGAGATCTACAAGGGTGACTGGAGTTCAGACGTGTGCTCTT	CGCAATGTAC	CCTTGTAGAT
AATGATACGGCGACCACCGAGATCTACACCCTAGAGAACACTCTTTCCCTACACGACGCTCT	CAAGCAGAAGACGGCATACGAGATCGATGTTCGTGACTGGAGTTCAGACGTGTGCTCTT	CCTAGAGAAC	GAACATCGAT
AATGATACGGCGACCACCGAGATCTACACTACTAGCGACACTCTTTCCCTACACGACGCTCT	CAAGCAGAAGACGGCATACGAGATACCGGTTAGTGACTGGAGTTCAGACGTGTGCTCTT	TACTAGCGAC	TAACCGGTAT
AATGATACGGCGACCACCGAGATCTACACCGTCCATTACACTCTTTCCCTACACGACGCTCT	CAAGCAGAAGACGGCATACGAGATGAACGGTTGTGACTGGAGTTCAGACGTGTGCTCTT	CGTCCATTAC	AACCGTTCAT
AATGATACGGCGACCACCGAGATCTACACTCGCTATCACACTCTTTCCCTACACGACGCTCT	CAAGCAGAAGACGGCATACGAGATCTGTACCAGTGACTGGAGTTCAGACGTGTGCTCTT	TCGCTATCAC	TGGTACAGAT
AATGATACGGCGACCACCGAGATCTACACAATGGTCGACACTCTTTCCCTACACGACGCTCT	CAAGCAGAAGACGGCATACGAGATGCGCATATGTGACTGGAGTTCAGACGTGTGCTCTT	AATGGTCGAC	ATATGCGCAT
AATGATACGGCGACCACCGAGATCTACACCGTAGAACAGACACTCTTTCCCTACACGACGCTCT	CAAGCAGAAGACGGCATACGAGATCCGCTAACAGGTGACTGGAGTTCAGACGTGTGCTCTT	CGTAGAACAG	CTGTTAGCGG
AATGATACGGCGACCACCGAGATCTACACCTGGCATATTACACTCTTTCCCTACACGACGCTCT	CAAGCAGAAGACGGCATACGAGATTTATCTTCCGGTGACTGGAGTTCAGACGTGTGCTCTT	CTGGCATATT	CGGAAGATAA
AATGATACGGCGACCACCGAGATCTACACAGAACCTCGCACACTCTTTCCCTACACGACGCTCT	CAAGCAGAAGACGGCATACGAGATAACGAAGTGTGTGACTGGAGTTCAGACGTGTGCTCTT	AGAACCTCGC	ACACTTCGTT
AATGATACGGCGACCACCGAGATCTACACTGTCGTTAAGACACTCTTTCCCTACACGACGCTCT	CAAGCAGAAGACGGCATACGAGATTTGAGCGACGGTGACTGGAGTTCAGACGTGTGCTCTT	TGTCGTTAAG	CGTCGCTCAA
AATGATACGGCGACCACCGAGATCTACACCATACCGCTGACACTCTTTCCCTACACGACGCTCT	CAAGCAGAAGACGGCATACGAGATTCGGCCAAGTGTGACTGGAGTTCAGACGTGTGCTCTT	CATACCGCTG	ACTTGGCCGA

AATGATACGGCGACCACCGAGATCTACACCTTCAATAGCACACTCTTTCCCTACACGACGCTCT	CAAGCAGAAGACGGCATACGAGATGCAATCAATCGTGACTGGAGTTCAGACGTGTGCTCTT	CTTCAATAGC	GATTGATTGC
AATGATACGGCGACCACCGAGATCTACACCTCAGTCCGTACACTCTTTCCCTACACGACGCTCT	CAAGCAGAAGACGGCATACGAGATACCGGAGTAAGTGACTGGAGTTCAGACGTGTGCTCTT	CTCAGTCCGT	TTACTCCGGT
AATGATACGGCGACCACCGAGATCTACACCGCTTACAATACACTCTTTCCCTACACGACGCTCT	CAAGCAGAAGACGGCATACGAGATTGCAAGCGTGGTGACTGGAGTTCAGACGTGTGCTCTT	CGCTTACAAT	CACGCTTGCA
AATGATACGGCGACCACCGAGATCTACACAGAGAGACCGACACTCTTTCCCTACACGACGCTCT	CAAGCAGAAGACGGCATACGAGATGTTCTGTCTAGTGACTGGAGTTCAGACGTGTGCTCTT	AGAGAGACCG	TAGACAGAAC
AATGATACGGCGACCACCGAGATCTACACTAGCACACTTACACTCTTTCCCTACACGACGCTCT	CAAGCAGAAGACGGCATACGAGATGAAGAGCAATGTGACTGGAGTTCAGACGTGTGCTCTT	TAGCACACTT	ATTGCTCTTC
AATGATACGGCGACCACCGAGATCTACACCGGAGCATCTACACTCTTTCCCTACACGACGCTCT	CAAGCAGAAGACGGCATACGAGATTATGTGTGTCGTGACTGGAGTTCAGACGTGTGCTCTT	CGGAGCATCT	GACACACATA
AATGATACGGCGACCACCGAGATCTACACCGCTTGGTTCACACTCTTTCCCTACACGACGCTCT	CAAGCAGAAGACGGCATACGAGATGCCGTTGAAGGTGACTGGAGTTCAGACGTGTGCTCTT	CGCTTGGTTC	CTTCAACGGC
AATGATACGGCGACCACCGAGATCTACACCTTGAAGAGGACACTCTTTCCCTACACGACGCTCT	CAAGCAGAAGACGGCATACGAGATTAGCCTGTGGGTGACTGGAGTTCAGACGTGTGCTCTT	CTTGAAGAGG	CCACAGGCTA
AATGATACGGCGACCACCGAGATCTACACAACGCCATTCACACTCTTTCCCTACACGACGCTCT	CAAGCAGAAGACGGCATACGAGATTATGCCGGCAGTGACTGGAGTTCAGACGTGTGCTCTT	AACGCCATTC	TGCCGGCATA
AATGATACGGCGACCACCGAGATCTACACACAATTGGTCACACTCTTTCCCTACACGACGCTCT	CAAGCAGAAGACGGCATACGAGATAGACGTTAGTGTGACTGGAGTTCAGACGTGTGCTCTT	ACAATTGGTC	ACTAACGTCT
AATGATACGGCGACCACCGAGATCTACACTAGTGTTACGACACTCTTTCCCTACACGACGCTCT	CAAGCAGAAGACGGCATACGAGATTAATCCGGAAGTGACTGGAGTTCAGACGTGTGCTCTT	TAGTGTTACG	TTCCGGATTA
AATGATACGGCGACCACCGAGATCTACACCTTCCTAGGCACACTCTTTCCCTACACGACGCTCT	CAAGCAGAAGACGGCATACGAGATAACACGAGAAGTGACTGGAGTTCAGACGTGTGCTCTT	CTTCCTAGGC	TTCTCGTGTT
AATGATACGGCGACCACCGAGATCTACACCTTCTCCAGCACACTCTTTCCCTACACGACGCTCT	CAAGCAGAAGACGGCATACGAGATACAACTCGAGGTGACTGGAGTTCAGACGTGTGCTCTT	CTTCTCCAGC	CTCGAGTTGT
AATGATACGGCGACCACCGAGATCTACACTAGCGCAATAACACTCTTTCCCTACACGACGCTCT	CAAGCAGAAGACGGCATACGAGATCGGCACATGAGTGACTGGAGTTCAGACGTGTGCTCTT	TAGCGCAATA	TCATGTGCCG
AATGATACGGCGACCACCGAGATCTACACTTAGACCACAACACTCTTTCCCTACACGACGCTCT	CAAGCAGAAGACGGCATACGAGATCTATCTCTCAGTGACTGGAGTTCAGACGTGTGCTCTT	TTAGACCACA	TGAGAGATAG
AATGATACGGCGACCACCGAGATCTACACGACCAAGCATACACTCTTTCCCTACACGACGCTCT	CAAGCAGAAGACGGCATACGAGATAAGTTACCAGGTGACTGGAGTTCAGACGTGTGCTCTT	GACCAAGCAT	CTGGTAACTT
AATGATACGGCGACCACCGAGATCTACACTATGCAACTCACACTCTTTCCCTACACGACGCTCT	CAAGCAGAAGACGGCATACGAGATTACCTTATGCGTGACTGGAGTTCAGACGTGTGCTCTT	TATGCAACTC	GCATAAGGTA
AATGATACGGCGACCACCGAGATCTACACTAACGCAGTCACACTCTTTCCCTACACGACGCTCT	CAAGCAGAAGACGGCATACGAGATTGTTAACCAGGTGACTGGAGTTCAGACGTGTGCTCTT	TAACGCAGTC	CTGGTTAACA
AATGATACGGCGACCACCGAGATCTACACCCATAAGCCAACACTCTTTCCCTACACGACGCTCT	CAAGCAGAAGACGGCATACGAGATTTGTACTCGCGTGACTGGAGTTCAGACGTGTGCTCTT	CCATAAGCCA	GCGAGTACAA
AATGATACGGCGACCACCGAGATCTACACCGGAACCAGTACACTCTTTCCCTACACGACGCTCT	CAAGCAGAAGACGGCATACGAGATAACCATCAAGGTGACTGGAGTTCAGACGTGTGCTCTT	CGGAACCAGT	CTTGATGGTT
AATGATACGGCGACCACCGAGATCTACACCGCCATCAATACACTCTTTCCCTACACGACGCTCT	CAAGCAGAAGACGGCATACGAGATTCCTTCACTAGTGACTGGAGTTCAGACGTGTGCTCTT	CGCCATCAAT	TAGTGAAGGA
AATGATACGGCGACCACCGAGATCTACACAGGACCTGTAACACTCTTTCCCTACACGACGCTCT	CAAGCAGAAGACGGCATACGAGATGTACACCATGGTGACTGGAGTTCAGACGTGTGCTCTT	AGGACCTGTA	CATGGTGTAC
AATGATACGGCGACCACCGAGATCTACACCTAGACACTCACACTCTTTCCCTACACGACGCTCT	CAAGCAGAAGACGGCATACGAGATTCTCGCATCGGTGACTGGAGTTCAGACGTGTGCTCTT	CTAGACACTC	CGATGCGAGA
AATGATACGGCGACCACCGAGATCTACACACATGGCCTGACACTCTTTCCCTACACGACGCTCT	CAAGCAGAAGACGGCATACGAGATTACGGCAGAAGTGACTGGAGTTCAGACGTGTGCTCTT	ACATGGCCTG	TTCTGCCGTA
AATGATACGGCGACCACCGAGATCTACACTCCAGCTCATACACTCTTTCCCTACACGACGCTCT	CAAGCAGAAGACGGCATACGAGATCCATACCGCAGTGACTGGAGTTCAGACGTGTGCTCTT	TCCAGCTCAT	TGCGGTATGG
AATGATACGGCGACCACCGAGATCTACACCACCAGCACAACACTCTTTCCCTACACGACGCTCT	CAAGCAGAAGACGGCATACGAGATAGTCGCATGGGTGACTGGAGTTCAGACGTGTGCTCTT	CACCAGCACA	CCATGCGACT
AATGATACGGCGACCACCGAGATCTACACCCGCGATTAGACACTCTTTCCCTACACGACGCTCT	CAAGCAGAAGACGGCATACGAGATACCACTGTTAGTGACTGGAGTTCAGACGTGTGCTCTT	CCGCGATTAG	TAACAGTGGT
AATGATACGGCGACCACCGAGATCTACACAGTGCCGATGACACTCTTTCCCTACACGACGCTCT	CAAGCAGAAGACGGCATACGAGATTAGTAACCGAGTGACTGGAGTTCAGACGTGTGCTCTT	AGTGCCGATG	TCGGTTACTA
AATGATACGGCGACCACCGAGATCTACACGATCTATTGGACACTCTTTCCCTACACGACGCTCT	CAAGCAGAAGACGGCATACGAGATGCACCGCTATGTGACTGGAGTTCAGACGTGTGCTCTT	GATCTATTGG	ATAGCGGTGC
AATGATACGGCGACCACCGAGATCTACACCATATTGGAGACACTCTTTCCCTACACGACGCTCT	CAAGCAGAAGACGGCATACGAGATTTGCTCACCAGTGACTGGAGTTCAGACGTGTGCTCTT	CATATTGGAG	TGGTGAGCAA
AATGATACGGCGACCACCGAGATCTACACAAGTCCATAGACACTCTTTCCCTACACGACGCTCT	CAAGCAGAAGACGGCATACGAGATTGGAACGGAAGTGACTGGAGTTCAGACGTGTGCTCTT	AAGTCCATAG	TTCCGTTCCA
AATGATACGGCGACCACCGAGATCTACACAGTACCATGAACACTCTTTCCCTACACGACGCTCT	CAAGCAGAAGACGGCATACGAGATAATTAAGCGGGTGACTGGAGTTCAGACGTGTGCTCTT	AGTACCATGA	CCGCTTAATT
AATGATACGGCGACCACCGAGATCTACACGCCGGTAAGTACACTCTTTCCCTACACGACGCTCT	CAAGCAGAAGACGGCATACGAGATAGACGAGGTCGTGACTGGAGTTCAGACGTGTGCTCTT	GCCGGTAAGT	GACCTCGTCT
AATGATACGGCGACCACCGAGATCTACACTACCAGAACGACACTCTTTCCCTACACGACGCTCT	CAAGCAGAAGACGGCATACGAGATCTAATAGGCTGTGACTGGAGTTCAGACGTGTGCTCTT	TACCAGAACG	AGCCTATTAG
AATGATACGGCGACCACCGAGATCTACACCATTGGCCAAACACTCTTTCCCTACACGACGCTCT	CAAGCAGAAGACGGCATACGAGATGCCTACCGATGTGACTGGAGTTCAGACGTGTGCTCTT	CATTGGCCAA	ATCGGTAGGC
AATGATACGGCGACCACCGAGATCTACACGCTGGCCAATACACTCTTTCCCTACACGACGCTCT	CAAGCAGAAGACGGCATACGAGATCTTATCAGGTGTGACTGGAGTTCAGACGTGTGCTCTT	GCTGGCCAAT	ACCTGATAAG
AATGATACGGCGACCACCGAGATCTACACCTACGTGGTAACACTCTTTCCCTACACGACGCTCT	CAAGCAGAAGACGGCATACGAGATTGCATATAGGGTGACTGGAGTTCAGACGTGTGCTCTT	CTACGTGGTA	CCTATATGCA
AATGATACGGCGACCACCGAGATCTACACCTTATTCGGAACACTCTTTCCCTACACGACGCTCT	CAAGCAGAAGACGGCATACGAGATGCACCTGATCGTGACTGGAGTTCAGACGTGTGCTCTT	CTTATTCGGA	GATCAGGTGC
AATGATACGGCGACCACCGAGATCTACACCTGGCATCCAACACTCTTTCCCTACACGACGCTCT	CAAGCAGAAGACGGCATACGAGATGTACTCGTGAGTGACTGGAGTTCAGACGTGTGCTCTT	CTGGCATCCA	TCACGAGTAC
AATGATACGGCGACCACCGAGATCTACACCCTAGCAATTACACTCTTTCCCTACACGACGCTCT	CAAGCAGAAGACGGCATACGAGATCACATATGCAGTGACTGGAGTTCAGACGTGTGCTCTT	CCTAGCAATT	TGCATATGTG
AATGATACGGCGACCACCGAGATCTACACCCTCCTCTTGACACTCTTTCCCTACACGACGCTCT	CAAGCAGAAGACGGCATACGAGATCGTACTATACGTGACTGGAGTTCAGACGTGTGCTCTT	CCTCCTCTTG	GTATAGTACG
AATGATACGGCGACCACCGAGATCTACACTCGTTGTAGCACACTCTTTCCCTACACGACGCTCT	CAAGCAGAAGACGGCATACGAGATTATCAGCCAAGTGACTGGAGTTCAGACGTGTGCTCTT	TCGTTGTAGC	TTGGCTGATA
AATGATACGGCGACCACCGAGATCTACACGCAGACTTCAACACTCTTTCCCTACACGACGCTCT	CAAGCAGAAGACGGCATACGAGATCCAAGCCATCGTGACTGGAGTTCAGACGTGTGCTCTT	GCAGACTTCA	GATGGCTTGG
AATGATACGGCGACCACCGAGATCTACACCCTTAAGCAGACACTCTTTCCCTACACGACGCTCT	CAAGCAGAAGACGGCATACGAGATGATCGCCTCAGTGACTGGAGTTCAGACGTGTGCTCTT	CCTTAAGCAG	TGAGGCGATC
AATGATACGGCGACCACCGAGATCTACACCCTCATAACTACACTCTTTCCCTACACGACGCTCT	CAAGCAGAAGACGGCATACGAGATGCTCTGAAGGGTGACTGGAGTTCAGACGTGTGCTCTT	CCTCATAACT	CCTTCAGAGC
AATGATACGGCGACCACCGAGATCTACACTTCATGGTGCACACTCTTTCCCTACACGACGCTCT	CAAGCAGAAGACGGCATACGAGATCTTGGCCTCTGTGACTGGAGTTCAGACGTGTGCTCTT	TTCATGGTGC	AGAGGCCAAG
AATGATACGGCGACCACCGAGATCTACACCGGACGGAATACACTCTTTCCCTACACGACGCTCT	CAAGCAGAAGACGGCATACGAGATTCATCGGAATGTGACTGGAGTTCAGACGTGTGCTCTT	CGGACGGAAT	ATTCCGATGA
AATGATACGGCGACCACCGAGATCTACACCCGTGAGCAAACACTCTTTCCCTACACGACGCTCT	CAAGCAGAAGACGGCATACGAGATTCACCGTATAGTGACTGGAGTTCAGACGTGTGCTCTT	CCGTGAGCAA	TATACGGTGA
AATGATACGGCGACCACCGAGATCTACACTTGGCATCGCACACTCTTTCCCTACACGACGCTCT	CAAGCAGAAGACGGCATACGAGATTGTTACCTCAGTGACTGGAGTTCAGACGTGTGCTCTT	TTGGCATCGC	TGAGGTAACA
AATGATACGGCGACCACCGAGATCTACACGATCTGGTTAACACTCTTTCCCTACACGACGCTCT	CAAGCAGAAGACGGCATACGAGATGAAGGCCTAAGTGACTGGAGTTCAGACGTGTGCTCTT	GATCTGGTTA	TTAGGCCTTC
AATGATACGGCGACCACCGAGATCTACACCCTATAATGCACACTCTTTCCCTACACGACGCTCT	CAAGCAGAAGACGGCATACGAGATTACGAATCGAGTGACTGGAGTTCAGACGTGTGCTCTT	CCTATAATGC	TCGATTCGTA
AATGATACGGCGACCACCGAGATCTACACGTAAGGAATCACACTCTTTCCCTACACGACGCTCT	CAAGCAGAAGACGGCATACGAGATTGTTAGAGGTGTGACTGGAGTTCAGACGTGTGCTCTT	GTAAGGAATC	ACCTCTAACA
AATGATACGGCGACCACCGAGATCTACACAAGTAACGTCACACTCTTTCCCTACACGACGCTCT	CAAGCAGAAGACGGCATACGAGATGTTCCTCATTGTGACTGGAGTTCAGACGTGTGCTCTT	AAGTAACGTC	AATGAGGAAC
AATGATACGGCGACCACCGAGATCTACACTCCAGTTCTCACACTCTTTCCCTACACGACGCTCT	CAAGCAGAAGACGGCATACGAGATCTGGTATTAGGTGACTGGAGTTCAGACGTGTGCTCTT	TCCAGTTCTC	CTAATACCAG
AATGATACGGCGACCACCGAGATCTACACCTGTAGCGCTACACTCTTTCCCTACACGACGCTCT	CAAGCAGAAGACGGCATACGAGATAGGCCTCCAAGTGACTGGAGTTCAGACGTGTGCTCTT	CTGTAGCGCT	TTGGAGGCCT
AATGATACGGCGACCACCGAGATCTACACAGACTGATCAACACTCTTTCCCTACACGACGCTCT	CAAGCAGAAGACGGCATACGAGATTCGGCTATGAGTGACTGGAGTTCAGACGTGTGCTCTT	AGACTGATCA	TCATAGCCGA
AATGATACGGCGACCACCGAGATCTACACCGGTTGAACGACACTCTTTCCCTACACGACGCTCT	CAAGCAGAAGACGGCATACGAGATGCGAGCCATAGTGACTGGAGTTCAGACGTGTGCTCTT	CGGTTGAACG	TATGGCTCGC
AATGATACGGCGACCACCGAGATCTACACAGAATGGAGGACACTCTTTCCCTACACGACGCTCT	CAAGCAGAAGACGGCATACGAGATCCTTCATAGGGTGACTGGAGTTCAGACGTGTGCTCTT	AGAATGGAGG	CCTATGAAGG
AATGATACGGCGACCACCGAGATCTACACGCGCGAACATACACTCTTTCCCTACACGACGCTCT	CAAGCAGAAGACGGCATACGAGATGCTTGAGACGGTGACTGGAGTTCAGACGTGTGCTCTT	GCGCGAACAT	CGTCTCAAGC
AATGATACGGCGACCACCGAGATCTACACACCGGCGAATACACTCTTTCCCTACACGACGCTCT	CAAGCAGAAGACGGCATACGAGATGAGGAGTATGGTGACTGGAGTTCAGACGTGTGCTCTT	ACCGGCGAAT	CATACTCCTC
AATGATACGGCGACCACCGAGATCTACACGCCAATTGCAACACTCTTTCCCTACACGACGCTCT	CAAGCAGAAGACGGCATACGAGATGCTTACACTTGTGACTGGAGTTCAGACGTGTGCTCTT	GCCAATTGCA	AAGTGTAAGC
AATGATACGGCGACCACCGAGATCTACACCCTGCAGCATACACTCTTTCCCTACACGACGCTCT	CAAGCAGAAGACGGCATACGAGATGTCGATGATTGTGACTGGAGTTCAGACGTGTGCTCTT	CCTGCAGCAT	AATCATCGAC
AATGATACGGCGACCACCGAGATCTACACCCAGAGGAATACACTCTTTCCCTACACGACGCTCT	CAAGCAGAAGACGGCATACGAGATACGCGAGTCAGTGACTGGAGTTCAGACGTGTGCTCTT	CCAGAGGAAT	TGACTCGCGT
AATGATACGGCGACCACCGAGATCTACACCCAATCAAGTACACTCTTTCCCTACACGACGCTCT	CAAGCAGAAGACGGCATACGAGATCTGTGAAGAAGTGACTGGAGTTCAGACGTGTGCTCTT	CCAATCAAGT	TTCTTCACAG
AATGATACGGCGACCACCGAGATCTACACCACGTTACTCACACTCTTTCCCTACACGACGCTCT	CAAGCAGAAGACGGCATACGAGATTACGCAACGGGTGACTGGAGTTCAGACGTGTGCTCTT	CACGTTACTC	CCGTTGCGTA
AATGATACGGCGACCACCGAGATCTACACCTACGTTCCAACACTCTTTCCCTACACGACGCTCT	CAAGCAGAAGACGGCATACGAGATTCCACTGCGTGTGACTGGAGTTCAGACGTGTGCTCTT	CTACGTTCCA	ACGCAGTGGA
AATGATACGGCGACCACCGAGATCTACACTAAGGTGACAACACTCTTTCCCTACACGACGCTCT	CAAGCAGAAGACGGCATACGAGATTTGAGCCTGGGTGACTGGAGTTCAGACGTGTGCTCTT	TAAGGTGACA	CCAGGCTCAA
AATGATACGGCGACCACCGAGATCTACACAAGCAGGCTCACACTCTTTCCCTACACGACGCTCT	CAAGCAGAAGACGGCATACGAGATTAGTGAATCGGTGACTGGAGTTCAGACGTGTGCTCTT	AAGCAGGCTC	CGATTCACTA
AATGATACGGCGACCACCGAGATCTACACGCTACACGAGACACTCTTTCCCTACACGACGCTCT	CAAGCAGAAGACGGCATACGAGATGCCTGTACTGGTGACTGGAGTTCAGACGTGTGCTCTT	GCTACACGAG	CAGTACAGGC
AATGATACGGCGACCACCGAGATCTACACTGCTATGCAGACACTCTTTCCCTACACGACGCTCT	CAAGCAGAAGACGGCATACGAGATGTGATAAGCTGTGACTGGAGTTCAGACGTGTGCTCTT	TGCTATGCAG	AGCTTATCAC
AATGATACGGCGACCACCGAGATCTACACACCGCTAGCAACACTCTTTCCCTACACGACGCTCT	CAAGCAGAAGACGGCATACGAGATCTACCACGAAGTGACTGGAGTTCAGACGTGTGCTCTT	ACCGCTAGCA	TTCGTGGTAG
AATGATACGGCGACCACCGAGATCTACACAGACAGTGGCACACTCTTTCCCTACACGACGCTCT	CAAGCAGAAGACGGCATACGAGATCCATATACGAGTGACTGGAGTTCAGACGTGTGCTCTT	AGACAGTGGC	TCGTATATGG
AATGATACGGCGACCACCGAGATCTACACGTGCAGATTAACACTCTTTCCCTACACGACGCTCT	CAAGCAGAAGACGGCATACGAGATGAACGGCCATGTGACTGGAGTTCAGACGTGTGCTCTT	GTGCAGATTA	ATGGCCGTTC
AATGATACGGCGACCACCGAGATCTACACCTTACGAGTAACACTCTTTCCCTACACGACGCTCT	CAAGCAGAAGACGGCATACGAGATAGTACGTGAAGTGACTGGAGTTCAGACGTGTGCTCTT	CTTACGAGTA	TTCACGTACT
AATGATACGGCGACCACCGAGATCTACACCCTCACAGATACACTCTTTCCCTACACGACGCTCT	CAAGCAGAAGACGGCATACGAGATGCTCGGAACTGTGACTGGAGTTCAGACGTGTGCTCTT	CCTCACAGAT	AGTTCCGAGC
AATGATACGGCGACCACCGAGATCTACACCCAACAGATTACACTCTTTCCCTACACGACGCTCT	CAAGCAGAAGACGGCATACGAGATCCAACATAGAGTGACTGGAGTTCAGACGTGTGCTCTT	CCAACAGATT	TCTATGTTGG
AATGATACGGCGACCACCGAGATCTACACTAGAGTGAGAACACTCTTTCCCTACACGACGCTCT	CAAGCAGAAGACGGCATACGAGATCAGTGTGGCAGTGACTGGAGTTCAGACGTGTGCTCTT	TAGAGTGAGA	TGCCACACTG
AATGATACGGCGACCACCGAGATCTACACTAAGGTTCGTACACTCTTTCCCTACACGACGCTCT	CAAGCAGAAGACGGCATACGAGATTCGAAGCGGAGTGACTGGAGTTCAGACGTGTGCTCTT	TAAGGTTCGT	TCCGCTTCGA
AATGATACGGCGACCACCGAGATCTACACTCGTTCGTAAACACTCTTTCCCTACACGACGCTCT	CAAGCAGAAGACGGCATACGAGATGCACTACGGTGTGACTGGAGTTCAGACGTGTGCTCTT	TCGTTCGTAA	ACCGTAGTGC
AATGATACGGCGACCACCGAGATCTACACTCAGCAGAGGACACTCTTTCCCTACACGACGCTCT	CAAGCAGAAGACGGCATACGAGATGTCGACTATAGTGACTGGAGTTCAGACGTGTGCTCTT	TCAGCAGAGG	TATAGTCGAC
AATGATACGGCGACCACCGAGATCTACACGCTAACGGCAACACTCTTTCCCTACACGACGCTCT	CAAGCAGAAGACGGCATACGAGATGCAAGTACGAGTGACTGGAGTTCAGACGTGTGCTCTT	GCTAACGGCA	TCGTACTTGC
AATGATACGGCGACCACCGAGATCTACACACGCAAGAAGACACTCTTTCCCTACACGACGCTCT	CAAGCAGAAGACGGCATACGAGATAGTGTTGTCTGTGACTGGAGTTCAGACGTGTGCTCTT	ACGCAAGAAG	AGACAACACT
AATGATACGGCGACCACCGAGATCTACACTAGAGGCTTAACACTCTTTCCCTACACGACGCTCT	CAAGCAGAAGACGGCATACGAGATCGGTTGCCTAGTGACTGGAGTTCAGACGTGTGCTCTT	TAGAGGCTTA	TAGGCAACCG
AATGATACGGCGACCACCGAGATCTACACAGTAGAGTGTACACTCTTTCCCTACACGACGCTCT	CAAGCAGAAGACGGCATACGAGATCGAATGGTAAGTGACTGGAGTTCAGACGTGTGCTCTT	AGTAGAGTGT	TTACCATTCG
AATGATACGGCGACCACCGAGATCTACACTCCTTGTCGGACACTCTTTCCCTACACGACGCTCT	CAAGCAGAAGACGGCATACGAGATCCGCAATAGGGTGACTGGAGTTCAGACGTGTGCTCTT	TCCTTGTCGG	CCTATTGCGG
AATGATACGGCGACCACCGAGATCTACACCACTAATCAGACACTCTTTCCCTACACGACGCTCT	CAAGCAGAAGACGGCATACGAGATCTGTGCGTAGGTGACTGGAGTTCAGACGTGTGCTCTT	CACTAATCAG	CTACGCACAG
AATGATACGGCGACCACCGAGATCTACACAACCGAGATTACACTCTTTCCCTACACGACGCTCT	CAAGCAGAAGACGGCATACGAGATTTGTGGCGCAGTGACTGGAGTTCAGACGTGTGCTCTT	AACCGAGATT	TGCGCCACAA
AATGATACGGCGACCACCGAGATCTACACTACACGAAGAACACTCTTTCCCTACACGACGCTCT	CAAGCAGAAGACGGCATACGAGATAAGGCAAGTTGTGACTGGAGTTCAGACGTGTGCTCTT	TACACGAAGA	AACTTGCCTT
AATGATACGGCGACCACCGAGATCTACACAGCGACAGGTACACTCTTTCCCTACACGACGCTCT	CAAGCAGAAGACGGCATACGAGATTTCTAGATGGGTGACTGGAGTTCAGACGTGTGCTCTT	AGCGACAGGT	CCATCTAGAA
AATGATACGGCGACCACCGAGATCTACACGAGTAACCTTACACTCTTTCCCTACACGACGCTCT	CAAGCAGAAGACGGCATACGAGATGTGGAACAATGTGACTGGAGTTCAGACGTGTGCTCTT	GAGTAACCTT	ATTGTTCCAC
AATGATACGGCGACCACCGAGATCTACACACGGCTAGAGACACTCTTTCCCTACACGACGCTCT	CAAGCAGAAGACGGCATACGAGATTGTGGAGAGGGTGACTGGAGTTCAGACGTGTGCTCTT	ACGGCTAGAG	CCTCTCCACA
AATGATACGGCGACCACCGAGATCTACACCTACCGCGTTACACTCTTTCCCTACACGACGCTCT	CAAGCAGAAGACGGCATACGAGATTGCTGCAGTCGTGACTGGAGTTCAGACGTGTGCTCTT	CTACCGCGTT	GACTGCAGCA
AATGATACGGCGACCACCGAGATCTACACGCAAGAGATGACACTCTTTCCCTACACGACGCTCT	CAAGCAGAAGACGGCATACGAGATCAGCTACAGTGTGACTGGAGTTCAGACGTGTGCTCTT	GCAAGAGATG	ACTGTAGCTG
AATGATACGGCGACCACCGAGATCTACACTCCTACAGCGACACTCTTTCCCTACACGACGCTCT	CAAGCAGAAGACGGCATACGAGATGATTATCTGCGTGACTGGAGTTCAGACGTGTGCTCTT	TCCTACAGCG	GCAGATAATC
AATGATACGGCGACCACCGAGATCTACACCCAGTGACGAACACTCTTTCCCTACACGACGCTCT	CAAGCAGAAGACGGCATACGAGATCATGAGCGCAGTGACTGGAGTTCAGACGTGTGCTCTT	CCAGTGACGA	TGCGCTCATG
AATGATACGGCGACCACCGAGATCTACACGTGGCGGTTAACACTCTTTCCCTACACGACGCTCT	CAAGCAGAAGACGGCATACGAGATTCTTAGGCCTGTGACTGGAGTTCAGACGTGTGCTCTT	GTGGCGGTTA	AGGCCTAAGA
AATGATACGGCGACCACCGAGATCTACACAGACACTCTAACACTCTTTCCCTACACGACGCTCT	CAAGCAGAAGACGGCATACGAGATAAGCGGATTAGTGACTGGAGTTCAGACGTGTGCTCTT	AGACACTCTA	TAATCCGCTT
AATGATACGGCGACCACCGAGATCTACACCCAAGCACAAACACTCTTTCCCTACACGACGCTCT	CAAGCAGAAGACGGCATACGAGATCGACTAAGAAGTGACTGGAGTTCAGACGTGTGCTCTT	CCAAGCACAA	TTCTTAGTCG

AATGATACGGCGACCACCGAGATCTACACTATGTGCGCTACACTCTTTCCCTACACGACGCTCT	CAAGCAGAAGACGGCATACGAGATCAACAACTGAGTGACTGGAGTTCAGACGTGTGCTCTT	TATGTGCGCT	TCAGTTGTTG
AATGATACGGCGACCACCGAGATCTACACCTGTTAGGATACACTCTTTCCCTACACGACGCTCT	CAAGCAGAAGACGGCATACGAGATCTCGAAGGAAGTGACTGGAGTTCAGACGTGTGCTCTT	CTGTTAGGAT	TTCCTTCGAG
AATGATACGGCGACCACCGAGATCTACACCGCATCTTGAACACTCTTTCCCTACACGACGCTCT	CAAGCAGAAGACGGCATACGAGATGAAGCTTACTGTGACTGGAGTTCAGACGTGTGCTCTT	CGCATCTTGA	AGTAAGCTTC
AATGATACGGCGACCACCGAGATCTACACAGAGGAGCAGACACTCTTTCCCTACACGACGCTCT	CAAGCAGAAGACGGCATACGAGATATGGCGCCTTGTGACTGGAGTTCAGACGTGTGCTCTT	AGAGGAGCAG	AAGGCGCCAT
AATGATACGGCGACCACCGAGATCTACACAGGTCGATAAACACTCTTTCCCTACACGACGCTCT	CAAGCAGAAGACGGCATACGAGATTCCTATTAGGGTGACTGGAGTTCAGACGTGTGCTCTT	AGGTCGATAA	CCTAATAGGA
AATGATACGGCGACCACCGAGATCTACACAGCGTAGCAAACACTCTTTCCCTACACGACGCTCT	CAAGCAGAAGACGGCATACGAGATACAATCACGAGTGACTGGAGTTCAGACGTGTGCTCTT	AGCGTAGCAA	TCGTGATTGT
AATGATACGGCGACCACCGAGATCTACACCTCAACGGAGACACTCTTTCCCTACACGACGCTCT	CAAGCAGAAGACGGCATACGAGATTGACCACCAGGTGACTGGAGTTCAGACGTGTGCTCTT	CTCAACGGAG	CTGGTGGTCA
AATGATACGGCGACCACCGAGATCTACACCTGTGCATTAACACTCTTTCCCTACACGACGCTCT	CAAGCAGAAGACGGCATACGAGATAACGTAGGCTGTGACTGGAGTTCAGACGTGTGCTCTT	CTGTGCATTA	AGCCTACGTT
AATGATACGGCGACCACCGAGATCTACACGTGCGTCGTAACACTCTTTCCCTACACGACGCTCT	CAAGCAGAAGACGGCATACGAGATCTTGAAGGTTGTGACTGGAGTTCAGACGTGTGCTCTT	GTGCGTCGTA	AACCTTCAAG
AATGATACGGCGACCACCGAGATCTACACCCATCCGAGTACACTCTTTCCCTACACGACGCTCT	CAAGCAGAAGACGGCATACGAGATTTGACGTGAAGTGACTGGAGTTCAGACGTGTGCTCTT	CCATCCGAGT	TTCACGTCAA
AATGATACGGCGACCACCGAGATCTACACCGCCACAGAAACACTCTTTCCCTACACGACGCTCT	CAAGCAGAAGACGGCATACGAGATACAACGCCAAGTGACTGGAGTTCAGACGTGTGCTCTT	CGCCACAGAA	TTGGCGTTGT
AATGATACGGCGACCACCGAGATCTACACGCATCTTCTTACACTCTTTCCCTACACGACGCTCT	CAAGCAGAAGACGGCATACGAGATCCGGTCTAGAGTGACTGGAGTTCAGACGTGTGCTCTT	GCATCTTCTT	TCTAGACCGG
AATGATACGGCGACCACCGAGATCTACACAAGATACAGCACACTCTTTCCCTACACGACGCTCT	CAAGCAGAAGACGGCATACGAGATGATTATGAGGGTGACTGGAGTTCAGACGTGTGCTCTT	AAGATACAGC	CCTCATAATC
AATGATACGGCGACCACCGAGATCTACACTTCCTAGCCGACACTCTTTCCCTACACGACGCTCT	CAAGCAGAAGACGGCATACGAGATCTCTGTGAGGGTGACTGGAGTTCAGACGTGTGCTCTT	TTCCTAGCCG	CCTCACAGAG
AATGATACGGCGACCACCGAGATCTACACACCTTCCGGTACACTCTTTCCCTACACGACGCTCT	CAAGCAGAAGACGGCATACGAGATAAGGTGAAGAGTGACTGGAGTTCAGACGTGTGCTCTT	ACCTTCCGGT	TCTTCACCTT
AATGATACGGCGACCACCGAGATCTACACTTACTAGGCAACACTCTTTCCCTACACGACGCTCT	CAAGCAGAAGACGGCATACGAGATGAGAGGTATAGTGACTGGAGTTCAGACGTGTGCTCTT	TTACTAGGCA	TATACCTCTC
AATGATACGGCGACCACCGAGATCTACACGAGTGTCTCAACACTCTTTCCCTACACGACGCTCT	CAAGCAGAAGACGGCATACGAGATTGTTAACTGCGTGACTGGAGTTCAGACGTGTGCTCTT	GAGTGTCTCA	GCAGTTAACA
AATGATACGGCGACCACCGAGATCTACACCGCTACACTCACACTCTTTCCCTACACGACGCTCT	CAAGCAGAAGACGGCATACGAGATTACGCGATAAGTGACTGGAGTTCAGACGTGTGCTCTT	CGCTACACTC	TTATCGCGTA
AATGATACGGCGACCACCGAGATCTACACTCGGATGAGGACACTCTTTCCCTACACGACGCTCT	CAAGCAGAAGACGGCATACGAGATCCTGGCTATAGTGACTGGAGTTCAGACGTGTGCTCTT	TCGGATGAGG	TATAGCCAGG
AATGATACGGCGACCACCGAGATCTACACGAGGCCACATACACTCTTTCCCTACACGACGCTCT	CAAGCAGAAGACGGCATACGAGATAACGGATAAGGTGACTGGAGTTCAGACGTGTGCTCTT	GAGGCCACAT	CTTATCCGTT
AATGATACGGCGACCACCGAGATCTACACCCATAAGAGGACACTCTTTCCCTACACGACGCTCT	CAAGCAGAAGACGGCATACGAGATCTACCTATCTGTGACTGGAGTTCAGACGTGTGCTCTT	CCATAAGAGG	AGATAGGTAG
AATGATACGGCGACCACCGAGATCTACACTTGGCTAGGCACACTCTTTCCCTACACGACGCTCT	CAAGCAGAAGACGGCATACGAGATCCGTATTAAGGTGACTGGAGTTCAGACGTGTGCTCTT	TTGGCTAGGC	CTTAATACGG
AATGATACGGCGACCACCGAGATCTACACTTAGAATCGGACACTCTTTCCCTACACGACGCTCT	CAAGCAGAAGACGGCATACGAGATTAACACCGGCGTGACTGGAGTTCAGACGTGTGCTCTT	TTAGAATCGG	GCCGGTGTTA
AATGATACGGCGACCACCGAGATCTACACGAACGGACTAACACTCTTTCCCTACACGACGCTCT	CAAGCAGAAGACGGCATACGAGATCGGCCATATGGTGACTGGAGTTCAGACGTGTGCTCTT	GAACGGACTA	CATATGGCCG
AATGATACGGCGACCACCGAGATCTACACTAATATCCGCACACTCTTTCCCTACACGACGCTCT	CAAGCAGAAGACGGCATACGAGATACTTATCCGGGTGACTGGAGTTCAGACGTGTGCTCTT	TAATATCCGC	CCGGATAAGT
AATGATACGGCGACCACCGAGATCTACACGCATTCAGCGACACTCTTTCCCTACACGACGCTCT	CAAGCAGAAGACGGCATACGAGATCTGGTCTCAAGTGACTGGAGTTCAGACGTGTGCTCTT	GCATTCAGCG	TTGAGACCAG
AATGATACGGCGACCACCGAGATCTACACAGGCCTTATAACACTCTTTCCCTACACGACGCTCT	CAAGCAGAAGACGGCATACGAGATACACACCGGAGTGACTGGAGTTCAGACGTGTGCTCTT	AGGCCTTATA	TCCGGTGTGT
AATGATACGGCGACCACCGAGATCTACACAACGGATCGCACACTCTTTCCCTACACGACGCTCT	CAAGCAGAAGACGGCATACGAGATGATGAACATCGTGACTGGAGTTCAGACGTGTGCTCTT	AACGGATCGC	GATGTTCATC
AATGATACGGCGACCACCGAGATCTACACTACATGCCGTACACTCTTTCCCTACACGACGCTCT	CAAGCAGAAGACGGCATACGAGATAACCTAGCCAGTGACTGGAGTTCAGACGTGTGCTCTT	TACATGCCGT	TGGCTAGGTT
AATGATACGGCGACCACCGAGATCTACACCTAGATAACGACACTCTTTCCCTACACGACGCTCT	CAAGCAGAAGACGGCATACGAGATCGGAATAGTAGTGACTGGAGTTCAGACGTGTGCTCTT	CTAGATAACG	TACTATTCCG
AATGATACGGCGACCACCGAGATCTACACAGAACCGCTAACACTCTTTCCCTACACGACGCTCT	CAAGCAGAAGACGGCATACGAGATTTATACCGTGGTGACTGGAGTTCAGACGTGTGCTCTT	AGAACCGCTA	CACGGTATAA
AATGATACGGCGACCACCGAGATCTACACTTGTCTGTAGACACTCTTTCCCTACACGACGCTCT	CAAGCAGAAGACGGCATACGAGATTCTTGCAGATGTGACTGGAGTTCAGACGTGTGCTCTT	TTGTCTGTAG	ATCTGCAAGA
AATGATACGGCGACCACCGAGATCTACACCGCTCGTGTTACACTCTTTCCCTACACGACGCTCT	CAAGCAGAAGACGGCATACGAGATGCGTGCTAGTGTGACTGGAGTTCAGACGTGTGCTCTT	CGCTCGTGTT	ACTAGCACGC
AATGATACGGCGACCACCGAGATCTACACGATCATTCCTACACTCTTTCCCTACACGACGCTCT	CAAGCAGAAGACGGCATACGAGATCGCATTCGAGGTGACTGGAGTTCAGACGTGTGCTCTT	GATCATTCCT	CTCGAATGCG
AATGATACGGCGACCACCGAGATCTACACCTTGAATTCGACACTCTTTCCCTACACGACGCTCT	CAAGCAGAAGACGGCATACGAGATCCTACCAGCTGTGACTGGAGTTCAGACGTGTGCTCTT	CTTGAATTCG	AGCTGGTAGG
AATGATACGGCGACCACCGAGATCTACACCCGTGTTCTCACACTCTTTCCCTACACGACGCTCT	CAAGCAGAAGACGGCATACGAGATCTTGATCCTAGTGACTGGAGTTCAGACGTGTGCTCTT	CCGTGTTCTC	TAGGATCAAG
AATGATACGGCGACCACCGAGATCTACACAGGCAGCTTAACACTCTTTCCCTACACGACGCTCT	CAAGCAGAAGACGGCATACGAGATCATCGACCGAGTGACTGGAGTTCAGACGTGTGCTCTT	AGGCAGCTTA	TCGGTCGATG
AATGATACGGCGACCACCGAGATCTACACTGACCTGCTGACACTCTTTCCCTACACGACGCTCT	CAAGCAGAAGACGGCATACGAGATGTCCGAGGAAGTGACTGGAGTTCAGACGTGTGCTCTT	TGACCTGCTG	TTCCTCGGAC
AATGATACGGCGACCACCGAGATCTACACCCTATTCAGTACACTCTTTCCCTACACGACGCTCT	CAAGCAGAAGACGGCATACGAGATCGTAACACTTGTGACTGGAGTTCAGACGTGTGCTCTT	CCTATTCAGT	AAGTGTTACG
AATGATACGGCGACCACCGAGATCTACACGTGTACACTAACACTCTTTCCCTACACGACGCTCT	CAAGCAGAAGACGGCATACGAGATTCACCTCTCGGTGACTGGAGTTCAGACGTGTGCTCTT	GTGTACACTA	CGAGAGGTGA
AATGATACGGCGACCACCGAGATCTACACTCGCACTCCAACACTCTTTCCCTACACGACGCTCT	CAAGCAGAAGACGGCATACGAGATAATAGGCTCTGTGACTGGAGTTCAGACGTGTGCTCTT	TCGCACTCCA	AGAGCCTATT
AATGATACGGCGACCACCGAGATCTACACCTGTGAATGTACACTCTTTCCCTACACGACGCTCT	CAAGCAGAAGACGGCATACGAGATGATGGAACTAGTGACTGGAGTTCAGACGTGTGCTCTT	CTGTGAATGT	TAGTTCCATC
AATGATACGGCGACCACCGAGATCTACACAGGTTACCTTACACTCTTTCCCTACACGACGCTCT	CAAGCAGAAGACGGCATACGAGATTCCGAAGTGGGTGACTGGAGTTCAGACGTGTGCTCTT	AGGTTACCTT	CCACTTCGGA
AATGATACGGCGACCACCGAGATCTACACTTGCCATCAGACACTCTTTCCCTACACGACGCTCT	CAAGCAGAAGACGGCATACGAGATGTATGACTTCGTGACTGGAGTTCAGACGTGTGCTCTT	TTGCCATCAG	GAAGTCATAC
AATGATACGGCGACCACCGAGATCTACACTTGTAGGACAACACTCTTTCCCTACACGACGCTCT	CAAGCAGAAGACGGCATACGAGATTGCAAGATGAGTGACTGGAGTTCAGACGTGTGCTCTT	TTGTAGGACA	TCATCTTGCA
AATGATACGGCGACCACCGAGATCTACACGCTACTTGAAACACTCTTTCCCTACACGACGCTCT	CAAGCAGAAGACGGCATACGAGATGTCAAGAACTGTGACTGGAGTTCAGACGTGTGCTCTT	GCTACTTGAA	AGTTCTTGAC
AATGATACGGCGACCACCGAGATCTACACACGCAAGTTCACACTCTTTCCCTACACGACGCTCT	CAAGCAGAAGACGGCATACGAGATCTAATCCGTTGTGACTGGAGTTCAGACGTGTGCTCTT	ACGCAAGTTC	AACGGATTAG
AATGATACGGCGACCACCGAGATCTACACCCTGCTAGGAACACTCTTTCCCTACACGACGCTCT	CAAGCAGAAGACGGCATACGAGATTTGTCTCTCTGTGACTGGAGTTCAGACGTGTGCTCTT	CCTGCTAGGA	AGAGAGACAA
AATGATACGGCGACCACCGAGATCTACACTGCGGACACAACACTCTTTCCCTACACGACGCTCT	CAAGCAGAAGACGGCATACGAGATCTACCGTTGAGTGACTGGAGTTCAGACGTGTGCTCTT	TGCGGACACA	TCAACGGTAG
AATGATACGGCGACCACCGAGATCTACACAGGACACATAACACTCTTTCCCTACACGACGCTCT	CAAGCAGAAGACGGCATACGAGATTGTGAATTGGGTGACTGGAGTTCAGACGTGTGCTCTT	AGGACACATA	CCAATTCACA
AATGATACGGCGACCACCGAGATCTACACGCAAGTATTGACACTCTTTCCCTACACGACGCTCT	CAAGCAGAAGACGGCATACGAGATGTGGCACAGAGTGACTGGAGTTCAGACGTGTGCTCTT	GCAAGTATTG	TCTGTGCCAC
AATGATACGGCGACCACCGAGATCTACACTGAATCGCGTACACTCTTTCCCTACACGACGCTCT	CAAGCAGAAGACGGCATACGAGATTAACCGCTAGGTGACTGGAGTTCAGACGTGTGCTCTT	TGAATCGCGT	CTAGCGGTTA
AATGATACGGCGACCACCGAGATCTACACCCAATCCTAAACACTCTTTCCCTACACGACGCTCT	CAAGCAGAAGACGGCATACGAGATACCAATTCGGGTGACTGGAGTTCAGACGTGTGCTCTT	CCAATCCTAA	CCGAATTGGT
AATGATACGGCGACCACCGAGATCTACACTTGGCTGCGTACACTCTTTCCCTACACGACGCTCT	CAAGCAGAAGACGGCATACGAGATGTCATGTGAAGTGACTGGAGTTCAGACGTGTGCTCTT	TTGGCTGCGT	TTCACATGAC
AATGATACGGCGACCACCGAGATCTACACGAGACAAGGAACACTCTTTCCCTACACGACGCTCT	CAAGCAGAAGACGGCATACGAGATTGCGTCATGGGTGACTGGAGTTCAGACGTGTGCTCTT	GAGACAAGGA	CCATGACGCA
AATGATACGGCGACCACCGAGATCTACACCCGGCCATTAACACTCTTTCCCTACACGACGCTCT	CAAGCAGAAGACGGCATACGAGATTATAGGCCGAGTGACTGGAGTTCAGACGTGTGCTCTT	CCGGCCATTA	TCGGCCTATA
AATGATACGGCGACCACCGAGATCTACACCCGTAGTTGGACACTCTTTCCCTACACGACGCTCT	CAAGCAGAAGACGGCATACGAGATCCATCAAGGAGTGACTGGAGTTCAGACGTGTGCTCTT	CCGTAGTTGG	TCCTTGATGG
AATGATACGGCGACCACCGAGATCTACACGTGCAGTAATACACTCTTTCCCTACACGACGCTCT	CAAGCAGAAGACGGCATACGAGATGCCGCAGATTGTGACTGGAGTTCAGACGTGTGCTCTT	GTGCAGTAAT	AATCTGCGGC
AATGATACGGCGACCACCGAGATCTACACTAACTCAGCAACACTCTTTCCCTACACGACGCTCT	CAAGCAGAAGACGGCATACGAGATTGCCTGAAGTGTGACTGGAGTTCAGACGTGTGCTCTT	TAACTCAGCA	ACTTCAGGCA
AATGATACGGCGACCACCGAGATCTACACTTCTGTTCTGACACTCTTTCCCTACACGACGCTCT	CAAGCAGAAGACGGCATACGAGATCCGAGTCTCTGTGACTGGAGTTCAGACGTGTGCTCTT	TTCTGTTCTG	AGAGACTCGG
AATGATACGGCGACCACCGAGATCTACACGCTTGTGCCAACACTCTTTCCCTACACGACGCTCT	CAAGCAGAAGACGGCATACGAGATCTAACGCCATGTGACTGGAGTTCAGACGTGTGCTCTT	GCTTGTGCCA	ATGGCGTTAG
AATGATACGGCGACCACCGAGATCTACACCGATAACCAAACACTCTTTCCCTACACGACGCTCT	CAAGCAGAAGACGGCATACGAGATTCACACTCTTGTGACTGGAGTTCAGACGTGTGCTCTT	CGATAACCAA	AAGAGTGTGA
AATGATACGGCGACCACCGAGATCTACACGATATACCTCACACTCTTTCCCTACACGACGCTCT	CAAGCAGAAGACGGCATACGAGATGCAATTAAGGGTGACTGGAGTTCAGACGTGTGCTCTT	GATATACCTC	CCTTAATTGC
AATGATACGGCGACCACCGAGATCTACACCACCAATAGTACACTCTTTCCCTACACGACGCTCT	CAAGCAGAAGACGGCATACGAGATGTCCAACCGTGTGACTGGAGTTCAGACGTGTGCTCTT	CACCAATAGT	ACGGTTGGAC
AATGATACGGCGACCACCGAGATCTACACTTCGTCAACTACACTCTTTCCCTACACGACGCTCT	CAAGCAGAAGACGGCATACGAGATGCAACAAGTTGTGACTGGAGTTCAGACGTGTGCTCTT	TTCGTCAACT	AACTTGTTGC
AATGATACGGCGACCACCGAGATCTACACCGTGTTCTTAACACTCTTTCCCTACACGACGCTCT	CAAGCAGAAGACGGCATACGAGATTGCCATCACAGTGACTGGAGTTCAGACGTGTGCTCTT	CGTGTTCTTA	TGTGATGGCA
AATGATACGGCGACCACCGAGATCTACACTCACAGCTGCACACTCTTTCCCTACACGACGCTCT	CAAGCAGAAGACGGCATACGAGATAGTGTGCAGAGTGACTGGAGTTCAGACGTGTGCTCTT	TCACAGCTGC	TCTGCACACT
AATGATACGGCGACCACCGAGATCTACACTGAACCACAAACACTCTTTCCCTACACGACGCTCT	CAAGCAGAAGACGGCATACGAGATGAATTCGACTGTGACTGGAGTTCAGACGTGTGCTCTT	TGAACCACAA	AGTCGAATTC
AATGATACGGCGACCACCGAGATCTACACCCGCTAGCTTACACTCTTTCCCTACACGACGCTCT	CAAGCAGAAGACGGCATACGAGATCGAGTCCTGAGTGACTGGAGTTCAGACGTGTGCTCTT	CCGCTAGCTT	TCAGGACTCG
AATGATACGGCGACCACCGAGATCTACACGCCTAAGAAGACACTCTTTCCCTACACGACGCTCT	CAAGCAGAAGACGGCATACGAGATCTAGCAACCTGTGACTGGAGTTCAGACGTGTGCTCTT	GCCTAAGAAG	AGGTTGCTAG
AATGATACGGCGACCACCGAGATCTACACTGTTGCACTAACACTCTTTCCCTACACGACGCTCT	CAAGCAGAAGACGGCATACGAGATGAGGATAACTGTGACTGGAGTTCAGACGTGTGCTCTT	TGTTGCACTA	AGTTATCCTC
AATGATACGGCGACCACCGAGATCTACACCCAGCTATAAACACTCTTTCCCTACACGACGCTCT	CAAGCAGAAGACGGCATACGAGATGTAAGATGAGGTGACTGGAGTTCAGACGTGTGCTCTT	CCAGCTATAA	CTCATCTTAC
AATGATACGGCGACCACCGAGATCTACACTGGCATCTGGACACTCTTTCCCTACACGACGCTCT	CAAGCAGAAGACGGCATACGAGATTGCCAGCTAAGTGACTGGAGTTCAGACGTGTGCTCTT	TGGCATCTGG	TTAGCTGGCA
AATGATACGGCGACCACCGAGATCTACACGTCTTATCAGACACTCTTTCCCTACACGACGCTCT	CAAGCAGAAGACGGCATACGAGATACCAGAAGTAGTGACTGGAGTTCAGACGTGTGCTCTT	GTCTTATCAG	TACTTCTGGT
AATGATACGGCGACCACCGAGATCTACACCAAGAGGAGGACACTCTTTCCCTACACGACGCTCT	CAAGCAGAAGACGGCATACGAGATCTCACACTTAGTGACTGGAGTTCAGACGTGTGCTCTT	CAAGAGGAGG	TAAGTGTGAG
AATGATACGGCGACCACCGAGATCTACACGCAATCGCAAACACTCTTTCCCTACACGACGCTCT	CAAGCAGAAGACGGCATACGAGATTAGCGCCAGTGTGACTGGAGTTCAGACGTGTGCTCTT	GCAATCGCAA	ACTGGCGCTA
AATGATACGGCGACCACCGAGATCTACACTCGGTGAATAACACTCTTTCCCTACACGACGCTCT	CAAGCAGAAGACGGCATACGAGATCTAAGAGCTAGTGACTGGAGTTCAGACGTGTGCTCTT	TCGGTGAATA	TAGCTCTTAG
AATGATACGGCGACCACCGAGATCTACACCGTAACATAGACACTCTTTCCCTACACGACGCTCT	CAAGCAGAAGACGGCATACGAGATTGCCAGAGCTGTGACTGGAGTTCAGACGTGTGCTCTT	CGTAACATAG	AGCTCTGGCA
AATGATACGGCGACCACCGAGATCTACACAAGCTAATGGACACTCTTTCCCTACACGACGCTCT	CAAGCAGAAGACGGCATACGAGATGACTATTCGTGTGACTGGAGTTCAGACGTGTGCTCTT	AAGCTAATGG	ACGAATAGTC
AATGATACGGCGACCACCGAGATCTACACCAATACCTTCACACTCTTTCCCTACACGACGCTCT	CAAGCAGAAGACGGCATACGAGATGCAGTAACATGTGACTGGAGTTCAGACGTGTGCTCTT	CAATACCTTC	ATGTTACTGC
AATGATACGGCGACCACCGAGATCTACACCTCATGATGAACACTCTTTCCCTACACGACGCTCT	CAAGCAGAAGACGGCATACGAGATACACCATGAAGTGACTGGAGTTCAGACGTGTGCTCTT	CTCATGATGA	TTCATGGTGT
AATGATACGGCGACCACCGAGATCTACACAACGAGCCTCACACTCTTTCCCTACACGACGCTCT	CAAGCAGAAGACGGCATACGAGATCGTTAGCACTGTGACTGGAGTTCAGACGTGTGCTCTT	AACGAGCCTC	AGTGCTAACG
AATGATACGGCGACCACCGAGATCTACACATGACTGCCGACACTCTTTCCCTACACGACGCTCT	CAAGCAGAAGACGGCATACGAGATGTAACTGCATGTGACTGGAGTTCAGACGTGTGCTCTT	ATGACTGCCG	ATGCAGTTAC
AATGATACGGCGACCACCGAGATCTACACGACATGAGTTACACTCTTTCCCTACACGACGCTCT	CAAGCAGAAGACGGCATACGAGATCTGGCAACTCGTGACTGGAGTTCAGACGTGTGCTCTT	GACATGAGTT	GAGTTGCCAG
AATGATACGGCGACCACCGAGATCTACACCTTAGTTGCAACACTCTTTCCCTACACGACGCTCT	CAAGCAGAAGACGGCATACGAGATGCTTAGTATGGTGACTGGAGTTCAGACGTGTGCTCTT	CTTAGTTGCA	CATACTAAGC
AATGATACGGCGACCACCGAGATCTACACCAGGCGTCAAACACTCTTTCCCTACACGACGCTCT	CAAGCAGAAGACGGCATACGAGATGTTAGTCGTCGTGACTGGAGTTCAGACGTGTGCTCTT	CAGGCGTCAA	GACGACTAAC
AATGATACGGCGACCACCGAGATCTACACGAAGCAACAAACACTCTTTCCCTACACGACGCTCT	CAAGCAGAAGACGGCATACGAGATTACGATAAGCGTGACTGGAGTTCAGACGTGTGCTCTT	GAAGCAACAA	GCTTATCGTA
AATGATACGGCGACCACCGAGATCTACACTAACTCGTGGACACTCTTTCCCTACACGACGCTCT	CAAGCAGAAGACGGCATACGAGATCGGCTACTTCGTGACTGGAGTTCAGACGTGTGCTCTT	TAACTCGTGG	GAAGTAGCCG
AATGATACGGCGACCACCGAGATCTACACGACATGTAGAACACTCTTTCCCTACACGACGCTCT	CAAGCAGAAGACGGCATACGAGATACAGCAGTGCGTGACTGGAGTTCAGACGTGTGCTCTT	GACATGTAGA	GCACTGCTGT
AATGATACGGCGACCACCGAGATCTACACTGCTGCAGTCACACTCTTTCCCTACACGACGCTCT	CAAGCAGAAGACGGCATACGAGATCTACCGCGTTGTGACTGGAGTTCAGACGTGTGCTCTT	TGCTGCAGTC	AACGCGGTAG
AATGATACGGCGACCACCGAGATCTACACCAGCTACAGTACACTCTTTCCCTACACGACGCTCT	CAAGCAGAAGACGGCATACGAGATGCAAGAGATGGTGACTGGAGTTCAGACGTGTGCTCTT	CAGCTACAGT	CATCTCTTGC
AATGATACGGCGACCACCGAGATCTACACGATTATCTGCACACTCTTTCCCTACACGACGCTCT	CAAGCAGAAGACGGCATACGAGATTCCTACAGCGGTGACTGGAGTTCAGACGTGTGCTCTT	GATTATCTGC	CGCTGTAGGA
AATGATACGGCGACCACCGAGATCTACACCATGAGCGCAACACTCTTTCCCTACACGACGCTCT	CAAGCAGAAGACGGCATACGAGATCCAGTGACGAGTGACTGGAGTTCAGACGTGTGCTCTT	CATGAGCGCA	TCGTCACTGG
AATGATACGGCGACCACCGAGATCTACACTCTTAGGCCTACACTCTTTCCCTACACGACGCTCT	CAAGCAGAAGACGGCATACGAGATGTGGCGGTTAGTGACTGGAGTTCAGACGTGTGCTCTT	TCTTAGGCCT	TAACCGCCAC
AATGATACGGCGACCACCGAGATCTACACAAGCGGATTAACACTCTTTCCCTACACGACGCTCT	CAAGCAGAAGACGGCATACGAGATAGACACTCTAGTGACTGGAGTTCAGACGTGTGCTCTT	AAGCGGATTA	TAGAGTGTCT
AATGATACGGCGACCACCGAGATCTACACCGACTAAGAAACACTCTTTCCCTACACGACGCTCT	CAAGCAGAAGACGGCATACGAGATCCAAGCACAAGTGACTGGAGTTCAGACGTGTGCTCTT	CGACTAAGAA	TTGTGCTTGG
AATGATACGGCGACCACCGAGATCTACACCAACAACTGAACACTCTTTCCCTACACGACGCTCT	CAAGCAGAAGACGGCATACGAGATTATGTGCGCTGTGACTGGAGTTCAGACGTGTGCTCTT	CAACAACTGA	AGCGCACATA
AATGATACGGCGACCACCGAGATCTACACCTCGAAGGAAACACTCTTTCCCTACACGACGCTCT	CAAGCAGAAGACGGCATACGAGATCTGTTAGGATGTGACTGGAGTTCAGACGTGTGCTCTT	CTCGAAGGAA	ATCCTAACAG

AATGATACGGCGACCACCGAGATCTACACGAAGCTTACTACACTCTTTCCCTACACGACGCTCT	CAAGCAGAAGACGGCATACGAGATCGCATCTTGAGTGACTGGAGTTCAGACGTGTGCTCTT	GAAGCTTACT	TCAAGATGCG
AATGATACGGCGACCACCGAGATCTACACATGGCGCCTTACACTCTTTCCCTACACGACGCTCT	CAAGCAGAAGACGGCATACGAGATAGAGGAGCAGGTGACTGGAGTTCAGACGTGTGCTCTT	ATGGCGCCTT	CTGCTCCTCT
AATGATACGGCGACCACCGAGATCTACACTCCTATTAGGACACTCTTTCCCTACACGACGCTCT	CAAGCAGAAGACGGCATACGAGATAGGTCGATAAGTGACTGGAGTTCAGACGTGTGCTCTT	TCCTATTAGG	TTATCGACCT
AATGATACGGCGACCACCGAGATCTACACACAATCACGAACACTCTTTCCCTACACGACGCTCT	CAAGCAGAAGACGGCATACGAGATAGCGTAGCAAGTGACTGGAGTTCAGACGTGTGCTCTT	ACAATCACGA	TTGCTACGCT
AATGATACGGCGACCACCGAGATCTACACTGACCACCAGACACTCTTTCCCTACACGACGCTCT	CAAGCAGAAGACGGCATACGAGATCTCAACGGAGGTGACTGGAGTTCAGACGTGTGCTCTT	TGACCACCAG	CTCCGTTGAG
AATGATACGGCGACCACCGAGATCTACACAACGTAGGCTACACTCTTTCCCTACACGACGCTCT	CAAGCAGAAGACGGCATACGAGATCTGTGCATTAGTGACTGGAGTTCAGACGTGTGCTCTT	AACGTAGGCT	TAATGCACAG
AATGATACGGCGACCACCGAGATCTACACCTTGAAGGTTACACTCTTTCCCTACACGACGCTCT	CAAGCAGAAGACGGCATACGAGATGTGCGTCGTAGTGACTGGAGTTCAGACGTGTGCTCTT	CTTGAAGGTT	TACGACGCAC
AATGATACGGCGACCACCGAGATCTACACTTGACGTGAAACACTCTTTCCCTACACGACGCTCT	CAAGCAGAAGACGGCATACGAGATCCATCCGAGTGTGACTGGAGTTCAGACGTGTGCTCTT	TTGACGTGAA	ACTCGGATGG
AATGATACGGCGACCACCGAGATCTACACACAACGCCAAACACTCTTTCCCTACACGACGCTCT	CAAGCAGAAGACGGCATACGAGATCGCCACAGAAGTGACTGGAGTTCAGACGTGTGCTCTT	ACAACGCCAA	TTCTGTGGCG
AATGATACGGCGACCACCGAGATCTACACCCGGTCTAGAACACTCTTTCCCTACACGACGCTCT	CAAGCAGAAGACGGCATACGAGATGCATCTTCTTGTGACTGGAGTTCAGACGTGTGCTCTT	CCGGTCTAGA	AAGAAGATGC
AATGATACGGCGACCACCGAGATCTACACGATTATGAGGACACTCTTTCCCTACACGACGCTCT	CAAGCAGAAGACGGCATACGAGATAAGATACAGCGTGACTGGAGTTCAGACGTGTGCTCTT	GATTATGAGG	GCTGTATCTT
AATGATACGGCGACCACCGAGATCTACACCTCTGTGAGGACACTCTTTCCCTACACGACGCTCT	CAAGCAGAAGACGGCATACGAGATTTCCTAGCCGGTGACTGGAGTTCAGACGTGTGCTCTT	CTCTGTGAGG	CGGCTAGGAA
AATGATACGGCGACCACCGAGATCTACACAAGGTGAAGAACACTCTTTCCCTACACGACGCTCT	CAAGCAGAAGACGGCATACGAGATACCTTCCGGTGTGACTGGAGTTCAGACGTGTGCTCTT	AAGGTGAAGA	ACCGGAAGGT
AATGATACGGCGACCACCGAGATCTACACGAGAGGTATAACACTCTTTCCCTACACGACGCTCT	CAAGCAGAAGACGGCATACGAGATTTACTAGGCAGTGACTGGAGTTCAGACGTGTGCTCTT	GAGAGGTATA	TGCCTAGTAA
AATGATACGGCGACCACCGAGATCTACACTGTTAACTGCACACTCTTTCCCTACACGACGCTCT	CAAGCAGAAGACGGCATACGAGATGAGTGTCTCAGTGACTGGAGTTCAGACGTGTGCTCTT	TGTTAACTGC	TGAGACACTC
AATGATACGGCGACCACCGAGATCTACACTACGCGATAAACACTCTTTCCCTACACGACGCTCT	CAAGCAGAAGACGGCATACGAGATCGCTACACTCGTGACTGGAGTTCAGACGTGTGCTCTT	TACGCGATAA	GAGTGTAGCG
AATGATACGGCGACCACCGAGATCTACACCCTGGCTATAACACTCTTTCCCTACACGACGCTCT	CAAGCAGAAGACGGCATACGAGATTCGGATGAGGGTGACTGGAGTTCAGACGTGTGCTCTT	CCTGGCTATA	CCTCATCCGA
AATGATACGGCGACCACCGAGATCTACACAACGGATAAGACACTCTTTCCCTACACGACGCTCT	CAAGCAGAAGACGGCATACGAGATGAGGCCACATGTGACTGGAGTTCAGACGTGTGCTCTT	AACGGATAAG	ATGTGGCCTC
AATGATACGGCGACCACCGAGATCTACACCTACCTATCTACACTCTTTCCCTACACGACGCTCT	CAAGCAGAAGACGGCATACGAGATCCATAAGAGGGTGACTGGAGTTCAGACGTGTGCTCTT	CTACCTATCT	CCTCTTATGG
AATGATACGGCGACCACCGAGATCTACACCCGTATTAAGACACTCTTTCCCTACACGACGCTCT	CAAGCAGAAGACGGCATACGAGATTTGGCTAGGCGTGACTGGAGTTCAGACGTGTGCTCTT	CCGTATTAAG	GCCTAGCCAA
AATGATACGGCGACCACCGAGATCTACACTAACACCGGCACACTCTTTCCCTACACGACGCTCT	CAAGCAGAAGACGGCATACGAGATTTAGAATCGGGTGACTGGAGTTCAGACGTGTGCTCTT	TAACACCGGC	CCGATTCTAA
AATGATACGGCGACCACCGAGATCTACACCGGCCATATGACACTCTTTCCCTACACGACGCTCT	CAAGCAGAAGACGGCATACGAGATGAACGGACTAGTGACTGGAGTTCAGACGTGTGCTCTT	CGGCCATATG	TAGTCCGTTC
AATGATACGGCGACCACCGAGATCTACACACTTATCCGGACACTCTTTCCCTACACGACGCTCT	CAAGCAGAAGACGGCATACGAGATTAATATCCGCGTGACTGGAGTTCAGACGTGTGCTCTT	ACTTATCCGG	GCGGATATTA
AATGATACGGCGACCACCGAGATCTACACCTGGTCTCAAACACTCTTTCCCTACACGACGCTCT	CAAGCAGAAGACGGCATACGAGATGCATTCAGCGGTGACTGGAGTTCAGACGTGTGCTCTT	CTGGTCTCAA	CGCTGAATGC
AATGATACGGCGACCACCGAGATCTACACACACACCGGAACACTCTTTCCCTACACGACGCTCT	CAAGCAGAAGACGGCATACGAGATAGGCCTTATAGTGACTGGAGTTCAGACGTGTGCTCTT	ACACACCGGA	TATAAGGCCT
AATGATACGGCGACCACCGAGATCTACACGATGAACATCACACTCTTTCCCTACACGACGCTCT	CAAGCAGAAGACGGCATACGAGATAACGGATCGCGTGACTGGAGTTCAGACGTGTGCTCTT	GATGAACATC	GCGATCCGTT
AATGATACGGCGACCACCGAGATCTACACAACCTAGCCAACACTCTTTCCCTACACGACGCTCT	CAAGCAGAAGACGGCATACGAGATTACATGCCGTGTGACTGGAGTTCAGACGTGTGCTCTT	AACCTAGCCA	ACGGCATGTA
AATGATACGGCGACCACCGAGATCTACACCGGAATAGTAACACTCTTTCCCTACACGACGCTCT	CAAGCAGAAGACGGCATACGAGATCTAGATAACGGTGACTGGAGTTCAGACGTGTGCTCTT	CGGAATAGTA	CGTTATCTAG
AATGATACGGCGACCACCGAGATCTACACTTATACCGTGACACTCTTTCCCTACACGACGCTCT	CAAGCAGAAGACGGCATACGAGATAGAACCGCTAGTGACTGGAGTTCAGACGTGTGCTCTT	TTATACCGTG	TAGCGGTTCT
AATGATACGGCGACCACCGAGATCTACACTCTTGCAGATACACTCTTTCCCTACACGACGCTCT	CAAGCAGAAGACGGCATACGAGATTTGTCTGTAGGTGACTGGAGTTCAGACGTGTGCTCTT	TCTTGCAGAT	CTACAGACAA
AATGATACGGCGACCACCGAGATCTACACGCGTGCTAGTACACTCTTTCCCTACACGACGCTCT	CAAGCAGAAGACGGCATACGAGATCGCTCGTGTTGTGACTGGAGTTCAGACGTGTGCTCTT	GCGTGCTAGT	AACACGAGCG
AATGATACGGCGACCACCGAGATCTACACCGCATTCGAGACACTCTTTCCCTACACGACGCTCT	CAAGCAGAAGACGGCATACGAGATGATCATTCCTGTGACTGGAGTTCAGACGTGTGCTCTT	CGCATTCGAG	AGGAATGATC
AATGATACGGCGACCACCGAGATCTACACCCTACCAGCTACACTCTTTCCCTACACGACGCTCT	CAAGCAGAAGACGGCATACGAGATCTTGAATTCGGTGACTGGAGTTCAGACGTGTGCTCTT	CCTACCAGCT	CGAATTCAAG
AATGATACGGCGACCACCGAGATCTACACCTTGATCCTAACACTCTTTCCCTACACGACGCTCT	CAAGCAGAAGACGGCATACGAGATCCGTGTTCTCGTGACTGGAGTTCAGACGTGTGCTCTT	CTTGATCCTA	GAGAACACGG
AATGATACGGCGACCACCGAGATCTACACCATCGACCGAACACTCTTTCCCTACACGACGCTCT	CAAGCAGAAGACGGCATACGAGATAGGCAGCTTAGTGACTGGAGTTCAGACGTGTGCTCTT	CATCGACCGA	TAAGCTGCCT
AATGATACGGCGACCACCGAGATCTACACGTCCGAGGAAACACTCTTTCCCTACACGACGCTCT	CAAGCAGAAGACGGCATACGAGATTGACCTGCTGGTGACTGGAGTTCAGACGTGTGCTCTT	GTCCGAGGAA	CAGCAGGTCA
AATGATACGGCGACCACCGAGATCTACACCGTAACACTTACACTCTTTCCCTACACGACGCTCT	CAAGCAGAAGACGGCATACGAGATCCTATTCAGTGTGACTGGAGTTCAGACGTGTGCTCTT	CGTAACACTT	ACTGAATAGG
AATGATACGGCGACCACCGAGATCTACACTCACCTCTCGACACTCTTTCCCTACACGACGCTCT	CAAGCAGAAGACGGCATACGAGATGTGTACACTAGTGACTGGAGTTCAGACGTGTGCTCTT	TCACCTCTCG	TAGTGTACAC
AATGATACGGCGACCACCGAGATCTACACAATAGGCTCTACACTCTTTCCCTACACGACGCTCT	CAAGCAGAAGACGGCATACGAGATTCGCACTCCAGTGACTGGAGTTCAGACGTGTGCTCTT	AATAGGCTCT	TGGAGTGCGA
AATGATACGGCGACCACCGAGATCTACACGATGGAACTAACACTCTTTCCCTACACGACGCTCT	CAAGCAGAAGACGGCATACGAGATCTGTGAATGTGTGACTGGAGTTCAGACGTGTGCTCTT	GATGGAACTA	ACATTCACAG
AATGATACGGCGACCACCGAGATCTACACTCCGAAGTGGACACTCTTTCCCTACACGACGCTCT	CAAGCAGAAGACGGCATACGAGATAGGTTACCTTGTGACTGGAGTTCAGACGTGTGCTCTT	TCCGAAGTGG	AAGGTAACCT
AATGATACGGCGACCACCGAGATCTACACGTATGACTTCACACTCTTTCCCTACACGACGCTCT	CAAGCAGAAGACGGCATACGAGATTTGCCATCAGGTGACTGGAGTTCAGACGTGTGCTCTT	GTATGACTTC	CTGATGGCAA
AATGATACGGCGACCACCGAGATCTACACTGCAAGATGAACACTCTTTCCCTACACGACGCTCT	CAAGCAGAAGACGGCATACGAGATTTGTAGGACAGTGACTGGAGTTCAGACGTGTGCTCTT	TGCAAGATGA	TGTCCTACAA
AATGATACGGCGACCACCGAGATCTACACGTCAAGAACTACACTCTTTCCCTACACGACGCTCT	CAAGCAGAAGACGGCATACGAGATGCTACTTGAAGTGACTGGAGTTCAGACGTGTGCTCTT	GTCAAGAACT	TTCAAGTAGC
AATGATACGGCGACCACCGAGATCTACACCTAATCCGTTACACTCTTTCCCTACACGACGCTCT	CAAGCAGAAGACGGCATACGAGATACGCAAGTTCGTGACTGGAGTTCAGACGTGTGCTCTT	CTAATCCGTT	GAACTTGCGT
AATGATACGGCGACCACCGAGATCTACACTTGTCTCTCTACACTCTTTCCCTACACGACGCTCT	CAAGCAGAAGACGGCATACGAGATCCTGCTAGGAGTGACTGGAGTTCAGACGTGTGCTCTT	TTGTCTCTCT	TCCTAGCAGG
AATGATACGGCGACCACCGAGATCTACACCTACCGTTGAACACTCTTTCCCTACACGACGCTCT	CAAGCAGAAGACGGCATACGAGATTGCGGACACAGTGACTGGAGTTCAGACGTGTGCTCTT	CTACCGTTGA	TGTGTCCGCA
AATGATACGGCGACCACCGAGATCTACACTGTGAATTGGACACTCTTTCCCTACACGACGCTCT	CAAGCAGAAGACGGCATACGAGATAGGACACATAGTGACTGGAGTTCAGACGTGTGCTCTT	TGTGAATTGG	TATGTGTCCT
AATGATACGGCGACCACCGAGATCTACACGTGGCACAGAACACTCTTTCCCTACACGACGCTCT	CAAGCAGAAGACGGCATACGAGATGCAAGTATTGGTGACTGGAGTTCAGACGTGTGCTCTT	GTGGCACAGA	CAATACTTGC
AATGATACGGCGACCACCGAGATCTACACTAACCGCTAGACACTCTTTCCCTACACGACGCTCT	CAAGCAGAAGACGGCATACGAGATTGAATCGCGTGTGACTGGAGTTCAGACGTGTGCTCTT	TAACCGCTAG	ACGCGATTCA
AATGATACGGCGACCACCGAGATCTACACACCAATTCGGACACTCTTTCCCTACACGACGCTCT	CAAGCAGAAGACGGCATACGAGATCCAATCCTAAGTGACTGGAGTTCAGACGTGTGCTCTT	ACCAATTCGG	TTAGGATTGG
AATGATACGGCGACCACCGAGATCTACACGTCATGTGAAACACTCTTTCCCTACACGACGCTCT	CAAGCAGAAGACGGCATACGAGATTTGGCTGCGTGTGACTGGAGTTCAGACGTGTGCTCTT	GTCATGTGAA	ACGCAGCCAA
AATGATACGGCGACCACCGAGATCTACACTGCGTCATGGACACTCTTTCCCTACACGACGCTCT	CAAGCAGAAGACGGCATACGAGATGAGACAAGGAGTGACTGGAGTTCAGACGTGTGCTCTT	TGCGTCATGG	TCCTTGTCTC
AATGATACGGCGACCACCGAGATCTACACTATAGGCCGAACACTCTTTCCCTACACGACGCTCT	CAAGCAGAAGACGGCATACGAGATCCGGCCATTAGTGACTGGAGTTCAGACGTGTGCTCTT	TATAGGCCGA	TAATGGCCGG
AATGATACGGCGACCACCGAGATCTACACCCATCAAGGAACACTCTTTCCCTACACGACGCTCT	CAAGCAGAAGACGGCATACGAGATCCGTAGTTGGGTGACTGGAGTTCAGACGTGTGCTCTT	CCATCAAGGA	CCAACTACGG
AATGATACGGCGACCACCGAGATCTACACGCCGCAGATTACACTCTTTCCCTACACGACGCTCT	CAAGCAGAAGACGGCATACGAGATGTGCAGTAATGTGACTGGAGTTCAGACGTGTGCTCTT	GCCGCAGATT	ATTACTGCAC
AATGATACGGCGACCACCGAGATCTACACTGCCTGAAGTACACTCTTTCCCTACACGACGCTCT	CAAGCAGAAGACGGCATACGAGATTAACTCAGCAGTGACTGGAGTTCAGACGTGTGCTCTT	TGCCTGAAGT	TGCTGAGTTA
AATGATACGGCGACCACCGAGATCTACACCCGAGTCTCTACACTCTTTCCCTACACGACGCTCT	CAAGCAGAAGACGGCATACGAGATTTCTGTTCTGGTGACTGGAGTTCAGACGTGTGCTCTT	CCGAGTCTCT	CAGAACAGAA
AATGATACGGCGACCACCGAGATCTACACCTAACGCCATACACTCTTTCCCTACACGACGCTCT	CAAGCAGAAGACGGCATACGAGATGCTTGTGCCAGTGACTGGAGTTCAGACGTGTGCTCTT	CTAACGCCAT	TGGCACAAGC
AATGATACGGCGACCACCGAGATCTACACTCACACTCTTACACTCTTTCCCTACACGACGCTCT	CAAGCAGAAGACGGCATACGAGATCGATAACCAAGTGACTGGAGTTCAGACGTGTGCTCTT	TCACACTCTT	TTGGTTATCG
AATGATACGGCGACCACCGAGATCTACACGCAATTAAGGACACTCTTTCCCTACACGACGCTCT	CAAGCAGAAGACGGCATACGAGATGATATACCTCGTGACTGGAGTTCAGACGTGTGCTCTT	GCAATTAAGG	GAGGTATATC
AATGATACGGCGACCACCGAGATCTACACGTCCAACCGTACACTCTTTCCCTACACGACGCTCT	CAAGCAGAAGACGGCATACGAGATCACCAATAGTGTGACTGGAGTTCAGACGTGTGCTCTT	GTCCAACCGT	ACTATTGGTG
AATGATACGGCGACCACCGAGATCTACACGCAACAAGTTACACTCTTTCCCTACACGACGCTCT	CAAGCAGAAGACGGCATACGAGATTTCGTCAACTGTGACTGGAGTTCAGACGTGTGCTCTT	GCAACAAGTT	AGTTGACGAA
AATGATACGGCGACCACCGAGATCTACACTGCCATCACAACACTCTTTCCCTACACGACGCTCT	CAAGCAGAAGACGGCATACGAGATCGTGTTCTTAGTGACTGGAGTTCAGACGTGTGCTCTT	TGCCATCACA	TAAGAACACG
AATGATACGGCGACCACCGAGATCTACACAGTGTGCAGAACACTCTTTCCCTACACGACGCTCT	CAAGCAGAAGACGGCATACGAGATTCACAGCTGCGTGACTGGAGTTCAGACGTGTGCTCTT	AGTGTGCAGA	GCAGCTGTGA
AATGATACGGCGACCACCGAGATCTACACGAATTCGACTACACTCTTTCCCTACACGACGCTCT	CAAGCAGAAGACGGCATACGAGATTGAACCACAAGTGACTGGAGTTCAGACGTGTGCTCTT	GAATTCGACT	TTGTGGTTCA
AATGATACGGCGACCACCGAGATCTACACCGAGTCCTGAACACTCTTTCCCTACACGACGCTCT	CAAGCAGAAGACGGCATACGAGATCCGCTAGCTTGTGACTGGAGTTCAGACGTGTGCTCTT	CGAGTCCTGA	AAGCTAGCGG
AATGATACGGCGACCACCGAGATCTACACCTAGCAACCTACACTCTTTCCCTACACGACGCTCT	CAAGCAGAAGACGGCATACGAGATGCCTAAGAAGGTGACTGGAGTTCAGACGTGTGCTCTT	CTAGCAACCT	CTTCTTAGGC
AATGATACGGCGACCACCGAGATCTACACGAGGATAACTACACTCTTTCCCTACACGACGCTCT	CAAGCAGAAGACGGCATACGAGATTGTTGCACTAGTGACTGGAGTTCAGACGTGTGCTCTT	GAGGATAACT	TAGTGCAACA
AATGATACGGCGACCACCGAGATCTACACGTAAGATGAGACACTCTTTCCCTACACGACGCTCT	CAAGCAGAAGACGGCATACGAGATCCAGCTATAAGTGACTGGAGTTCAGACGTGTGCTCTT	GTAAGATGAG	TTATAGCTGG
AATGATACGGCGACCACCGAGATCTACACTGCCAGCTAAACACTCTTTCCCTACACGACGCTCT	CAAGCAGAAGACGGCATACGAGATTGGCATCTGGGTGACTGGAGTTCAGACGTGTGCTCTT	TGCCAGCTAA	CCAGATGCCA
AATGATACGGCGACCACCGAGATCTACACACCAGAAGTAACACTCTTTCCCTACACGACGCTCT	CAAGCAGAAGACGGCATACGAGATGTCTTATCAGGTGACTGGAGTTCAGACGTGTGCTCTT	ACCAGAAGTA	CTGATAAGAC
AATGATACGGCGACCACCGAGATCTACACCTCACACTTAACACTCTTTCCCTACACGACGCTCT	CAAGCAGAAGACGGCATACGAGATCAAGAGGAGGGTGACTGGAGTTCAGACGTGTGCTCTT	CTCACACTTA	CCTCCTCTTG
AATGATACGGCGACCACCGAGATCTACACTAGCGCCAGTACACTCTTTCCCTACACGACGCTCT	CAAGCAGAAGACGGCATACGAGATGCAATCGCAAGTGACTGGAGTTCAGACGTGTGCTCTT	TAGCGCCAGT	TTGCGATTGC
AATGATACGGCGACCACCGAGATCTACACCTAAGAGCTAACACTCTTTCCCTACACGACGCTCT	CAAGCAGAAGACGGCATACGAGATTCGGTGAATAGTGACTGGAGTTCAGACGTGTGCTCTT	CTAAGAGCTA	TATTCACCGA
AATGATACGGCGACCACCGAGATCTACACTGCCAGAGCTACACTCTTTCCCTACACGACGCTCT	CAAGCAGAAGACGGCATACGAGATCGTAACATAGGTGACTGGAGTTCAGACGTGTGCTCTT	TGCCAGAGCT	CTATGTTACG
AATGATACGGCGACCACCGAGATCTACACGACTATTCGTACACTCTTTCCCTACACGACGCTCT	CAAGCAGAAGACGGCATACGAGATAAGCTAATGGGTGACTGGAGTTCAGACGTGTGCTCTT	GACTATTCGT	CCATTAGCTT
AATGATACGGCGACCACCGAGATCTACACGCAGTAACATACACTCTTTCCCTACACGACGCTCT	CAAGCAGAAGACGGCATACGAGATCAATACCTTCGTGACTGGAGTTCAGACGTGTGCTCTT	GCAGTAACAT	GAAGGTATTG
AATGATACGGCGACCACCGAGATCTACACACACCATGAAACACTCTTTCCCTACACGACGCTCT	CAAGCAGAAGACGGCATACGAGATCTCATGATGAGTGACTGGAGTTCAGACGTGTGCTCTT	ACACCATGAA	TCATCATGAG
AATGATACGGCGACCACCGAGATCTACACCGTTAGCACTACACTCTTTCCCTACACGACGCTCT	CAAGCAGAAGACGGCATACGAGATAACGAGCCTCGTGACTGGAGTTCAGACGTGTGCTCTT	CGTTAGCACT	GAGGCTCGTT
AATGATACGGCGACCACCGAGATCTACACGTAACTGCATACACTCTTTCCCTACACGACGCTCT	CAAGCAGAAGACGGCATACGAGATATGACTGCCGGTGACTGGAGTTCAGACGTGTGCTCTT	GTAACTGCAT	CGGCAGTCAT
AATGATACGGCGACCACCGAGATCTACACCTGGCAACTCACACTCTTTCCCTACACGACGCTCT	CAAGCAGAAGACGGCATACGAGATGACATGAGTTGTGACTGGAGTTCAGACGTGTGCTCTT	CTGGCAACTC	AACTCATGTC
AATGATACGGCGACCACCGAGATCTACACGCTTAGTATGACACTCTTTCCCTACACGACGCTCT	CAAGCAGAAGACGGCATACGAGATCTTAGTTGCAGTGACTGGAGTTCAGACGTGTGCTCTT	GCTTAGTATG	TGCAACTAAG
AATGATACGGCGACCACCGAGATCTACACGTTAGTCGTCACACTCTTTCCCTACACGACGCTCT	CAAGCAGAAGACGGCATACGAGATCAGGCGTCAAGTGACTGGAGTTCAGACGTGTGCTCTT	GTTAGTCGTC	TTGACGCCTG
AATGATACGGCGACCACCGAGATCTACACTACGATAAGCACACTCTTTCCCTACACGACGCTCT	CAAGCAGAAGACGGCATACGAGATGAAGCAACAAGTGACTGGAGTTCAGACGTGTGCTCTT	TACGATAAGC	TTGTTGCTTC
AATGATACGGCGACCACCGAGATCTACACCGGCTACTTCACACTCTTTCCCTACACGACGCTCT	CAAGCAGAAGACGGCATACGAGATTAACTCGTGGGTGACTGGAGTTCAGACGTGTGCTCTT	CGGCTACTTC	CCACGAGTTA
AATGATACGGCGACCACCGAGATCTACACACAGCAGTGCACACTCTTTCCCTACACGACGCTCT	CAAGCAGAAGACGGCATACGAGATGACATGTAGAGTGACTGGAGTTCAGACGTGTGCTCTT	ACAGCAGTGC	TCTACATGTC
AATGATACGGCGACCACCGAGATCTACACCCGCTAACAGACACTCTTTCCCTACACGACGCTCT	CAAGCAGAAGACGGCATACGAGATCGTAGAACAGGTGACTGGAGTTCAGACGTGTGCTCTT	CCGCTAACAG	CTGTTCTACG
AATGATACGGCGACCACCGAGATCTACACTTATCTTCCGACACTCTTTCCCTACACGACGCTCT	CAAGCAGAAGACGGCATACGAGATCTGGCATATTGTGACTGGAGTTCAGACGTGTGCTCTT	TTATCTTCCG	AATATGCCAG
AATGATACGGCGACCACCGAGATCTACACAACGAAGTGTACACTCTTTCCCTACACGACGCTCT	CAAGCAGAAGACGGCATACGAGATAGAACCTCGCGTGACTGGAGTTCAGACGTGTGCTCTT	AACGAAGTGT	GCGAGGTTCT
AATGATACGGCGACCACCGAGATCTACACTTGAGCGACGACACTCTTTCCCTACACGACGCTCT	CAAGCAGAAGACGGCATACGAGATTGTCGTTAAGGTGACTGGAGTTCAGACGTGTGCTCTT	TTGAGCGACG	CTTAACGACA
AATGATACGGCGACCACCGAGATCTACACTCGGCCAAGTACACTCTTTCCCTACACGACGCTCT	CAAGCAGAAGACGGCATACGAGATCATACCGCTGGTGACTGGAGTTCAGACGTGTGCTCTT	TCGGCCAAGT	CAGCGGTATG
AATGATACGGCGACCACCGAGATCTACACGCAATCAATCACACTCTTTCCCTACACGACGCTCT	CAAGCAGAAGACGGCATACGAGATCTTCAATAGCGTGACTGGAGTTCAGACGTGTGCTCTT	GCAATCAATC	GCTATTGAAG
AATGATACGGCGACCACCGAGATCTACACACCGGAGTAAACACTCTTTCCCTACACGACGCTCT	CAAGCAGAAGACGGCATACGAGATCTCAGTCCGTGTGACTGGAGTTCAGACGTGTGCTCTT	ACCGGAGTAA	ACGGACTGAG
AATGATACGGCGACCACCGAGATCTACACTGCAAGCGTGACACTCTTTCCCTACACGACGCTCT	CAAGCAGAAGACGGCATACGAGATCGCTTACAATGTGACTGGAGTTCAGACGTGTGCTCTT	TGCAAGCGTG	ATTGTAAGCG
AATGATACGGCGACCACCGAGATCTACACGTTCTGTCTAACACTCTTTCCCTACACGACGCTCT	CAAGCAGAAGACGGCATACGAGATAGAGAGACCGGTGACTGGAGTTCAGACGTGTGCTCTT	GTTCTGTCTA	CGGTCTCTCT
AATGATACGGCGACCACCGAGATCTACACGAAGAGCAATACACTCTTTCCCTACACGACGCTCT	CAAGCAGAAGACGGCATACGAGATTAGCACACTTGTGACTGGAGTTCAGACGTGTGCTCTT	GAAGAGCAAT	AAGTGTGCTA
AATGATACGGCGACCACCGAGATCTACACTATGTGTGTCACACTCTTTCCCTACACGACGCTCT	CAAGCAGAAGACGGCATACGAGATCGGAGCATCTGTGACTGGAGTTCAGACGTGTGCTCTT	TATGTGTGTC	AGATGCTCCG

AATGATACGGCGACCACCGAGATCTACACGCCGTTGAAGACACTCTTTCCCTACACGACGCTCT	CAAGCAGAAGACGGCATACGAGATCGCTTGGTTCGTGACTGGAGTTCAGACGTGTGCTCTT	GCCGTTGAAG	GAACCAAGCG
AATGATACGGCGACCACCGAGATCTACACTAGCCTGTGGACACTCTTTCCCTACACGACGCTCT	CAAGCAGAAGACGGCATACGAGATCTTGAAGAGGGTGACTGGAGTTCAGACGTGTGCTCTT	TAGCCTGTGG	CCTCTTCAAG
AATGATACGGCGACCACCGAGATCTACACTATGCCGGCAACACTCTTTCCCTACACGACGCTCT	CAAGCAGAAGACGGCATACGAGATAACGCCATTCGTGACTGGAGTTCAGACGTGTGCTCTT	TATGCCGGCA	GAATGGCGTT
AATGATACGGCGACCACCGAGATCTACACAGACGTTAGTACACTCTTTCCCTACACGACGCTCT	CAAGCAGAAGACGGCATACGAGATACAATTGGTCGTGACTGGAGTTCAGACGTGTGCTCTT	AGACGTTAGT	GACCAATTGT
AATGATACGGCGACCACCGAGATCTACACTAATCCGGAAACACTCTTTCCCTACACGACGCTCT	CAAGCAGAAGACGGCATACGAGATTAGTGTTACGGTGACTGGAGTTCAGACGTGTGCTCTT	TAATCCGGAA	CGTAACACTA
AATGATACGGCGACCACCGAGATCTACACAACACGAGAAACACTCTTTCCCTACACGACGCTCT	CAAGCAGAAGACGGCATACGAGATCTTCCTAGGCGTGACTGGAGTTCAGACGTGTGCTCTT	AACACGAGAA	GCCTAGGAAG
AATGATACGGCGACCACCGAGATCTACACACAACTCGAGACACTCTTTCCCTACACGACGCTCT	CAAGCAGAAGACGGCATACGAGATCTTCTCCAGCGTGACTGGAGTTCAGACGTGTGCTCTT	ACAACTCGAG	GCTGGAGAAG
AATGATACGGCGACCACCGAGATCTACACCGGCACATGAACACTCTTTCCCTACACGACGCTCT	CAAGCAGAAGACGGCATACGAGATTAGCGCAATAGTGACTGGAGTTCAGACGTGTGCTCTT	CGGCACATGA	TATTGCGCTA
AATGATACGGCGACCACCGAGATCTACACCTATCTCTCAACACTCTTTCCCTACACGACGCTCT	CAAGCAGAAGACGGCATACGAGATTTAGACCACAGTGACTGGAGTTCAGACGTGTGCTCTT	CTATCTCTCA	TGTGGTCTAA
AATGATACGGCGACCACCGAGATCTACACAAGTTACCAGACACTCTTTCCCTACACGACGCTCT	CAAGCAGAAGACGGCATACGAGATGACCAAGCATGTGACTGGAGTTCAGACGTGTGCTCTT	AAGTTACCAG	ATGCTTGGTC
AATGATACGGCGACCACCGAGATCTACACTACCTTATGCACACTCTTTCCCTACACGACGCTCT	CAAGCAGAAGACGGCATACGAGATTATGCAACTCGTGACTGGAGTTCAGACGTGTGCTCTT	TACCTTATGC	GAGTTGCATA
AATGATACGGCGACCACCGAGATCTACACTGTTAACCAGACACTCTTTCCCTACACGACGCTCT	CAAGCAGAAGACGGCATACGAGATTAACGCAGTCGTGACTGGAGTTCAGACGTGTGCTCTT	TGTTAACCAG	GACTGCGTTA
AATGATACGGCGACCACCGAGATCTACACTTGTACTCGCACACTCTTTCCCTACACGACGCTCT	CAAGCAGAAGACGGCATACGAGATCCATAAGCCAGTGACTGGAGTTCAGACGTGTGCTCTT	TTGTACTCGC	TGGCTTATGG
AATGATACGGCGACCACCGAGATCTACACAACCATCAAGACACTCTTTCCCTACACGACGCTCT	CAAGCAGAAGACGGCATACGAGATCGGAACCAGTGTGACTGGAGTTCAGACGTGTGCTCTT	AACCATCAAG	ACTGGTTCCG
AATGATACGGCGACCACCGAGATCTACACTCCTTCACTAACACTCTTTCCCTACACGACGCTCT	CAAGCAGAAGACGGCATACGAGATCGCCATCAATGTGACTGGAGTTCAGACGTGTGCTCTT	TCCTTCACTA	ATTGATGGCG
AATGATACGGCGACCACCGAGATCTACACGTACACCATGACACTCTTTCCCTACACGACGCTCT	CAAGCAGAAGACGGCATACGAGATAGGACCTGTAGTGACTGGAGTTCAGACGTGTGCTCTT	GTACACCATG	TACAGGTCCT
AATGATACGGCGACCACCGAGATCTACACTCTCGCATCGACACTCTTTCCCTACACGACGCTCT	CAAGCAGAAGACGGCATACGAGATCTAGACACTCGTGACTGGAGTTCAGACGTGTGCTCTT	TCTCGCATCG	GAGTGTCTAG
AATGATACGGCGACCACCGAGATCTACACTACGGCAGAAACACTCTTTCCCTACACGACGCTCT	CAAGCAGAAGACGGCATACGAGATACATGGCCTGGTGACTGGAGTTCAGACGTGTGCTCTT	TACGGCAGAA	CAGGCCATGT
AATGATACGGCGACCACCGAGATCTACACCCATACCGCAACACTCTTTCCCTACACGACGCTCT	CAAGCAGAAGACGGCATACGAGATTCCAGCTCATGTGACTGGAGTTCAGACGTGTGCTCTT	CCATACCGCA	ATGAGCTGGA
AATGATACGGCGACCACCGAGATCTACACAGTCGCATGGACACTCTTTCCCTACACGACGCTCT	CAAGCAGAAGACGGCATACGAGATCACCAGCACAGTGACTGGAGTTCAGACGTGTGCTCTT	AGTCGCATGG	TGTGCTGGTG
AATGATACGGCGACCACCGAGATCTACACACCACTGTTAACACTCTTTCCCTACACGACGCTCT	CAAGCAGAAGACGGCATACGAGATCCGCGATTAGGTGACTGGAGTTCAGACGTGTGCTCTT	ACCACTGTTA	CTAATCGCGG
AATGATACGGCGACCACCGAGATCTACACTAGTAACCGAACACTCTTTCCCTACACGACGCTCT	CAAGCAGAAGACGGCATACGAGATAGTGCCGATGGTGACTGGAGTTCAGACGTGTGCTCTT	TAGTAACCGA	CATCGGCACT
AATGATACGGCGACCACCGAGATCTACACGCACCGCTATACACTCTTTCCCTACACGACGCTCT	CAAGCAGAAGACGGCATACGAGATGATCTATTGGGTGACTGGAGTTCAGACGTGTGCTCTT	GCACCGCTAT	CCAATAGATC
AATGATACGGCGACCACCGAGATCTACACTTGCTCACCAACACTCTTTCCCTACACGACGCTCT	CAAGCAGAAGACGGCATACGAGATCATATTGGAGGTGACTGGAGTTCAGACGTGTGCTCTT	TTGCTCACCA	CTCCAATATG
AATGATACGGCGACCACCGAGATCTACACTGGAACGGAAACACTCTTTCCCTACACGACGCTCT	CAAGCAGAAGACGGCATACGAGATAAGTCCATAGGTGACTGGAGTTCAGACGTGTGCTCTT	TGGAACGGAA	CTATGGACTT
AATGATACGGCGACCACCGAGATCTACACAATTAAGCGGACACTCTTTCCCTACACGACGCTCT	CAAGCAGAAGACGGCATACGAGATAGTACCATGAGTGACTGGAGTTCAGACGTGTGCTCTT	AATTAAGCGG	TCATGGTACT
AATGATACGGCGACCACCGAGATCTACACAGACGAGGTCACACTCTTTCCCTACACGACGCTCT	CAAGCAGAAGACGGCATACGAGATGCCGGTAAGTGTGACTGGAGTTCAGACGTGTGCTCTT	AGACGAGGTC	ACTTACCGGC
AATGATACGGCGACCACCGAGATCTACACCTAATAGGCTACACTCTTTCCCTACACGACGCTCT	CAAGCAGAAGACGGCATACGAGATTACCAGAACGGTGACTGGAGTTCAGACGTGTGCTCTT	CTAATAGGCT	CGTTCTGGTA
AATGATACGGCGACCACCGAGATCTACACGCCTACCGATACACTCTTTCCCTACACGACGCTCT	CAAGCAGAAGACGGCATACGAGATCATTGGCCAAGTGACTGGAGTTCAGACGTGTGCTCTT	GCCTACCGAT	TTGGCCAATG
AATGATACGGCGACCACCGAGATCTACACCTTATCAGGTACACTCTTTCCCTACACGACGCTCT	CAAGCAGAAGACGGCATACGAGATGCTGGCCAATGTGACTGGAGTTCAGACGTGTGCTCTT	CTTATCAGGT	ATTGGCCAGC
AATGATACGGCGACCACCGAGATCTACACTGCATATAGGACACTCTTTCCCTACACGACGCTCT	CAAGCAGAAGACGGCATACGAGATCTACGTGGTAGTGACTGGAGTTCAGACGTGTGCTCTT	TGCATATAGG	TACCACGTAG
AATGATACGGCGACCACCGAGATCTACACGCACCTGATCACACTCTTTCCCTACACGACGCTCT	CAAGCAGAAGACGGCATACGAGATCTTATTCGGAGTGACTGGAGTTCAGACGTGTGCTCTT	GCACCTGATC	TCCGAATAAG
AATGATACGGCGACCACCGAGATCTACACGTACTCGTGAACACTCTTTCCCTACACGACGCTCT	CAAGCAGAAGACGGCATACGAGATCTGGCATCCAGTGACTGGAGTTCAGACGTGTGCTCTT	GTACTCGTGA	TGGATGCCAG
AATGATACGGCGACCACCGAGATCTACACCACATATGCAACACTCTTTCCCTACACGACGCTCT	CAAGCAGAAGACGGCATACGAGATCCTAGCAATTGTGACTGGAGTTCAGACGTGTGCTCTT	CACATATGCA	AATTGCTAGG
AATGATACGGCGACCACCGAGATCTACACCGTACTATACACACTCTTTCCCTACACGACGCTCT	CAAGCAGAAGACGGCATACGAGATCCTCCTCTTGGTGACTGGAGTTCAGACGTGTGCTCTT	CGTACTATAC	CAAGAGGAGG
AATGATACGGCGACCACCGAGATCTACACTATCAGCCAAACACTCTTTCCCTACACGACGCTCT	CAAGCAGAAGACGGCATACGAGATTCGTTGTAGCGTGACTGGAGTTCAGACGTGTGCTCTT	TATCAGCCAA	GCTACAACGA
AATGATACGGCGACCACCGAGATCTACACCCAAGCCATCACACTCTTTCCCTACACGACGCTCT	CAAGCAGAAGACGGCATACGAGATGCAGACTTCAGTGACTGGAGTTCAGACGTGTGCTCTT	CCAAGCCATC	TGAAGTCTGC
AATGATACGGCGACCACCGAGATCTACACGATCGCCTCAACACTCTTTCCCTACACGACGCTCT	CAAGCAGAAGACGGCATACGAGATCCTTAAGCAGGTGACTGGAGTTCAGACGTGTGCTCTT	GATCGCCTCA	CTGCTTAAGG
AATGATACGGCGACCACCGAGATCTACACGCTCTGAAGGACACTCTTTCCCTACACGACGCTCT	CAAGCAGAAGACGGCATACGAGATCCTCATAACTGTGACTGGAGTTCAGACGTGTGCTCTT	GCTCTGAAGG	AGTTATGAGG
AATGATACGGCGACCACCGAGATCTACACCTTGGCCTCTACACTCTTTCCCTACACGACGCTCT	CAAGCAGAAGACGGCATACGAGATTTCATGGTGCGTGACTGGAGTTCAGACGTGTGCTCTT	CTTGGCCTCT	GCACCATGAA
AATGATACGGCGACCACCGAGATCTACACTCATCGGAATACACTCTTTCCCTACACGACGCTCT	CAAGCAGAAGACGGCATACGAGATCGGACGGAATGTGACTGGAGTTCAGACGTGTGCTCTT	TCATCGGAAT	ATTCCGTCCG
AATGATACGGCGACCACCGAGATCTACACTCACCGTATAACACTCTTTCCCTACACGACGCTCT	CAAGCAGAAGACGGCATACGAGATCCGTGAGCAAGTGACTGGAGTTCAGACGTGTGCTCTT	TCACCGTATA	TTGCTCACGG
AATGATACGGCGACCACCGAGATCTACACTGTTACCTCAACACTCTTTCCCTACACGACGCTCT	CAAGCAGAAGACGGCATACGAGATTTGGCATCGCGTGACTGGAGTTCAGACGTGTGCTCTT	TGTTACCTCA	GCGATGCCAA
AATGATACGGCGACCACCGAGATCTACACGAAGGCCTAAACACTCTTTCCCTACACGACGCTCT	CAAGCAGAAGACGGCATACGAGATGATCTGGTTAGTGACTGGAGTTCAGACGTGTGCTCTT	GAAGGCCTAA	TAACCAGATC
AATGATACGGCGACCACCGAGATCTACACTACGAATCGAACACTCTTTCCCTACACGACGCTCT	CAAGCAGAAGACGGCATACGAGATCCTATAATGCGTGACTGGAGTTCAGACGTGTGCTCTT	TACGAATCGA	GCATTATAGG
AATGATACGGCGACCACCGAGATCTACACTGTTAGAGGTACACTCTTTCCCTACACGACGCTCT	CAAGCAGAAGACGGCATACGAGATGTAAGGAATCGTGACTGGAGTTCAGACGTGTGCTCTT	TGTTAGAGGT	GATTCCTTAC
AATGATACGGCGACCACCGAGATCTACACGTTCCTCATTACACTCTTTCCCTACACGACGCTCT	CAAGCAGAAGACGGCATACGAGATAAGTAACGTCGTGACTGGAGTTCAGACGTGTGCTCTT	GTTCCTCATT	GACGTTACTT
AATGATACGGCGACCACCGAGATCTACACCTGGTATTAGACACTCTTTCCCTACACGACGCTCT	CAAGCAGAAGACGGCATACGAGATTCCAGTTCTCGTGACTGGAGTTCAGACGTGTGCTCTT	CTGGTATTAG	GAGAACTGGA
AATGATACGGCGACCACCGAGATCTACACAGGCCTCCAAACACTCTTTCCCTACACGACGCTCT	CAAGCAGAAGACGGCATACGAGATCTGTAGCGCTGTGACTGGAGTTCAGACGTGTGCTCTT	AGGCCTCCAA	AGCGCTACAG
AATGATACGGCGACCACCGAGATCTACACTCGGCTATGAACACTCTTTCCCTACACGACGCTCT	CAAGCAGAAGACGGCATACGAGATAGACTGATCAGTGACTGGAGTTCAGACGTGTGCTCTT	TCGGCTATGA	TGATCAGTCT
AATGATACGGCGACCACCGAGATCTACACGCGAGCCATAACACTCTTTCCCTACACGACGCTCT	CAAGCAGAAGACGGCATACGAGATCGGTTGAACGGTGACTGGAGTTCAGACGTGTGCTCTT	GCGAGCCATA	CGTTCAACCG
AATGATACGGCGACCACCGAGATCTACACCCTTCATAGGACACTCTTTCCCTACACGACGCTCT	CAAGCAGAAGACGGCATACGAGATAGAATGGAGGGTGACTGGAGTTCAGACGTGTGCTCTT	CCTTCATAGG	CCTCCATTCT
AATGATACGGCGACCACCGAGATCTACACGCTTGAGACGACACTCTTTCCCTACACGACGCTCT	CAAGCAGAAGACGGCATACGAGATGCGCGAACATGTGACTGGAGTTCAGACGTGTGCTCTT	GCTTGAGACG	ATGTTCGCGC
AATGATACGGCGACCACCGAGATCTACACGAGGAGTATGACACTCTTTCCCTACACGACGCTCT	CAAGCAGAAGACGGCATACGAGATACCGGCGAATGTGACTGGAGTTCAGACGTGTGCTCTT	GAGGAGTATG	ATTCGCCGGT
AATGATACGGCGACCACCGAGATCTACACGCTTACACTTACACTCTTTCCCTACACGACGCTCT	CAAGCAGAAGACGGCATACGAGATGCCAATTGCAGTGACTGGAGTTCAGACGTGTGCTCTT	GCTTACACTT	TGCAATTGGC
AATGATACGGCGACCACCGAGATCTACACGTCGATGATTACACTCTTTCCCTACACGACGCTCT	CAAGCAGAAGACGGCATACGAGATCCTGCAGCATGTGACTGGAGTTCAGACGTGTGCTCTT	GTCGATGATT	ATGCTGCAGG
AATGATACGGCGACCACCGAGATCTACACACGCGAGTCAACACTCTTTCCCTACACGACGCTCT	CAAGCAGAAGACGGCATACGAGATCCAGAGGAATGTGACTGGAGTTCAGACGTGTGCTCTT	ACGCGAGTCA	ATTCCTCTGG
AATGATACGGCGACCACCGAGATCTACACCTGTGAAGAAACACTCTTTCCCTACACGACGCTCT	CAAGCAGAAGACGGCATACGAGATCCAATCAAGTGTGACTGGAGTTCAGACGTGTGCTCTT	CTGTGAAGAA	ACTTGATTGG
AATGATACGGCGACCACCGAGATCTACACTACGCAACGGACACTCTTTCCCTACACGACGCTCT	CAAGCAGAAGACGGCATACGAGATCACGTTACTCGTGACTGGAGTTCAGACGTGTGCTCTT	TACGCAACGG	GAGTAACGTG
AATGATACGGCGACCACCGAGATCTACACTCCACTGCGTACACTCTTTCCCTACACGACGCTCT	CAAGCAGAAGACGGCATACGAGATCTACGTTCCAGTGACTGGAGTTCAGACGTGTGCTCTT	TCCACTGCGT	TGGAACGTAG
AATGATACGGCGACCACCGAGATCTACACTTGAGCCTGGACACTCTTTCCCTACACGACGCTCT	CAAGCAGAAGACGGCATACGAGATTAAGGTGACAGTGACTGGAGTTCAGACGTGTGCTCTT	TTGAGCCTGG	TGTCACCTTA
AATGATACGGCGACCACCGAGATCTACACTAGTGAATCGACACTCTTTCCCTACACGACGCTCT	CAAGCAGAAGACGGCATACGAGATAAGCAGGCTCGTGACTGGAGTTCAGACGTGTGCTCTT	TAGTGAATCG	GAGCCTGCTT
AATGATACGGCGACCACCGAGATCTACACGCCTGTACTGACACTCTTTCCCTACACGACGCTCT	CAAGCAGAAGACGGCATACGAGATGCTACACGAGGTGACTGGAGTTCAGACGTGTGCTCTT	GCCTGTACTG	CTCGTGTAGC
AATGATACGGCGACCACCGAGATCTACACGTGATAAGCTACACTCTTTCCCTACACGACGCTCT	CAAGCAGAAGACGGCATACGAGATTGCTATGCAGGTGACTGGAGTTCAGACGTGTGCTCTT	GTGATAAGCT	CTGCATAGCA
AATGATACGGCGACCACCGAGATCTACACCTACCACGAAACACTCTTTCCCTACACGACGCTCT	CAAGCAGAAGACGGCATACGAGATACCGCTAGCAGTGACTGGAGTTCAGACGTGTGCTCTT	CTACCACGAA	TGCTAGCGGT
AATGATACGGCGACCACCGAGATCTACACCCATATACGAACACTCTTTCCCTACACGACGCTCT	CAAGCAGAAGACGGCATACGAGATAGACAGTGGCGTGACTGGAGTTCAGACGTGTGCTCTT	CCATATACGA	GCCACTGTCT
AATGATACGGCGACCACCGAGATCTACACGAACGGCCATACACTCTTTCCCTACACGACGCTCT	CAAGCAGAAGACGGCATACGAGATGTGCAGATTAGTGACTGGAGTTCAGACGTGTGCTCTT	GAACGGCCAT	TAATCTGCAC
AATGATACGGCGACCACCGAGATCTACACAGTACGTGAAACACTCTTTCCCTACACGACGCTCT	CAAGCAGAAGACGGCATACGAGATCTTACGAGTAGTGACTGGAGTTCAGACGTGTGCTCTT	AGTACGTGAA	TACTCGTAAG
AATGATACGGCGACCACCGAGATCTACACGCTCGGAACTACACTCTTTCCCTACACGACGCTCT	CAAGCAGAAGACGGCATACGAGATCCTCACAGATGTGACTGGAGTTCAGACGTGTGCTCTT	GCTCGGAACT	ATCTGTGAGG
AATGATACGGCGACCACCGAGATCTACACCCAACATAGAACACTCTTTCCCTACACGACGCTCT	CAAGCAGAAGACGGCATACGAGATCCAACAGATTGTGACTGGAGTTCAGACGTGTGCTCTT	CCAACATAGA	AATCTGTTGG
AATGATACGGCGACCACCGAGATCTACACCAGTGTGGCAACACTCTTTCCCTACACGACGCTCT	CAAGCAGAAGACGGCATACGAGATTAGAGTGAGAGTGACTGGAGTTCAGACGTGTGCTCTT	CAGTGTGGCA	TCTCACTCTA
AATGATACGGCGACCACCGAGATCTACACTCGAAGCGGAACACTCTTTCCCTACACGACGCTCT	CAAGCAGAAGACGGCATACGAGATTAAGGTTCGTGTGACTGGAGTTCAGACGTGTGCTCTT	TCGAAGCGGA	ACGAACCTTA
AATGATACGGCGACCACCGAGATCTACACGCACTACGGTACACTCTTTCCCTACACGACGCTCT	CAAGCAGAAGACGGCATACGAGATTCGTTCGTAAGTGACTGGAGTTCAGACGTGTGCTCTT	GCACTACGGT	TTACGAACGA
AATGATACGGCGACCACCGAGATCTACACGTCGACTATAACACTCTTTCCCTACACGACGCTCT	CAAGCAGAAGACGGCATACGAGATTCAGCAGAGGGTGACTGGAGTTCAGACGTGTGCTCTT	GTCGACTATA	CCTCTGCTGA
AATGATACGGCGACCACCGAGATCTACACGCAAGTACGAACACTCTTTCCCTACACGACGCTCT	CAAGCAGAAGACGGCATACGAGATGCTAACGGCAGTGACTGGAGTTCAGACGTGTGCTCTT	GCAAGTACGA	TGCCGTTAGC
AATGATACGGCGACCACCGAGATCTACACAGTGTTGTCTACACTCTTTCCCTACACGACGCTCT	CAAGCAGAAGACGGCATACGAGATACGCAAGAAGGTGACTGGAGTTCAGACGTGTGCTCTT	AGTGTTGTCT	CTTCTTGCGT
AATGATACGGCGACCACCGAGATCTACACCGGTTGCCTAACACTCTTTCCCTACACGACGCTCT	CAAGCAGAAGACGGCATACGAGATTAGAGGCTTAGTGACTGGAGTTCAGACGTGTGCTCTT	CGGTTGCCTA	TAAGCCTCTA
AATGATACGGCGACCACCGAGATCTACACCGAATGGTAAACACTCTTTCCCTACACGACGCTCT	CAAGCAGAAGACGGCATACGAGATAGTAGAGTGTGTGACTGGAGTTCAGACGTGTGCTCTT	CGAATGGTAA	ACACTCTACT
AATGATACGGCGACCACCGAGATCTACACCCGCAATAGGACACTCTTTCCCTACACGACGCTCT	CAAGCAGAAGACGGCATACGAGATTCCTTGTCGGGTGACTGGAGTTCAGACGTGTGCTCTT	CCGCAATAGG	CCGACAAGGA
AATGATACGGCGACCACCGAGATCTACACCTGTGCGTAGACACTCTTTCCCTACACGACGCTCT	CAAGCAGAAGACGGCATACGAGATCACTAATCAGGTGACTGGAGTTCAGACGTGTGCTCTT	CTGTGCGTAG	CTGATTAGTG
AATGATACGGCGACCACCGAGATCTACACTTGTGGCGCAACACTCTTTCCCTACACGACGCTCT	CAAGCAGAAGACGGCATACGAGATAACCGAGATTGTGACTGGAGTTCAGACGTGTGCTCTT	TTGTGGCGCA	AATCTCGGTT
AATGATACGGCGACCACCGAGATCTACACAAGGCAAGTTACACTCTTTCCCTACACGACGCTCT	CAAGCAGAAGACGGCATACGAGATTACACGAAGAGTGACTGGAGTTCAGACGTGTGCTCTT	AAGGCAAGTT	TCTTCGTGTA
AATGATACGGCGACCACCGAGATCTACACTTCTAGATGGACACTCTTTCCCTACACGACGCTCT	CAAGCAGAAGACGGCATACGAGATAGCGACAGGTGTGACTGGAGTTCAGACGTGTGCTCTT	TTCTAGATGG	ACCTGTCGCT
AATGATACGGCGACCACCGAGATCTACACGTGGAACAATACACTCTTTCCCTACACGACGCTCT	CAAGCAGAAGACGGCATACGAGATGAGTAACCTTGTGACTGGAGTTCAGACGTGTGCTCTT	GTGGAACAAT	AAGGTTACTC
AATGATACGGCGACCACCGAGATCTACACTGTGGAGAGGACACTCTTTCCCTACACGACGCTCT	CAAGCAGAAGACGGCATACGAGATACGGCTAGAGGTGACTGGAGTTCAGACGTGTGCTCTT	TGTGGAGAGG	CTCTAGCCGT

Supplementary References

1. Mo, A. *et al.* Epigenomic Signatures of Neuronal Diversity in the Mammalian Brain. *Neuron* **86,** 1369–1384 (2015).
2. Luo, C. *et al.* Single-cell methylomes identify neuronal subtypes and regulatory elements in mammalian cortex. *Science* **357,** 600–604 (2017).
3. Smallwood, S. A. *et al.* Single-cell genome-wide bisulfite sequencing for assessing epigenetic heterogeneity. *Nat. Methods* **11,** 817–820 (2014).
4. Farlik, M. *et al.* Single-cell DNA methylome sequencing and bioinformatic inference of epigenomic cell-state dynamics. *Cell Rep.* **10,** 1386–1397 (2015).
