# Supplementary material for: Robust single-cell DNA methylome profiling with snmC-seq2
Source: Nat Commun. 2018 Sep 20;9:3824. doi: 10.1038/s41467-018-06355-2 (PMC6147798; doi:10.1038/s41467-018-06355-2)
Supplement: Supplementary file 2 — Description of Additional Supplementary Files [file 41467_2018_6355_MOESM2_ESM.docx]

**Description of Additional Supplementary Files**

File Name: Supplementary Software 1

Description: Robotic scripts for automated snmC-seq2 library preparation.
